# Thermostable Proteins from HaCaT Keratinocytes Identify a Wide Breadth of Intrinsically Disordered Proteins and Candidates for Liquid–Liquid Phase Separation

**DOI:** 10.3390/ijms232214323

**Published:** 2022-11-18

**Authors:** Michael L. Samulevich, Rambon Shamilov, Brian J. Aneskievich

**Affiliations:** 1 Graduate Program in Pharmacology & Toxicology, Department of Pharmaceutical Sciences, University of Connecticut, 69 North Eagleville Road, Storrs, CT 06292-3092, USA; 2 Department of Pharmaceutical Sciences, School of Pharmacy, University of Connecticut, 69 North Eagleville Road, Storrs, CT 06269-3092, USA

**Keywords:** biochemistry, bioinformatics, keratinocyte biology, keratinocyte differentiation

## Abstract

Intrinsically disordered proteins (IDPs) move through an ensemble of conformations which allows multitudinous roles within a cell. Keratinocytes, the predominant cell type in mammalian epidermis, have had only a few individual proteins assessed for intrinsic disorder and its possible contribution to liquid–liquid phase separation (LLPS), especially in regard to what functions or structures these proteins provide. We took a holistic approach to keratinocyte IDPs starting with enrichment via the isolation of thermostable proteins. The keratinocyte protein involucrin, known for its resistance to heat denaturation, served as a marker. It and other thermostable proteins were identified by liquid chromatography tandem mass spectrometry and subjected to extensive bioinformatic analysis covering gene ontology, intrinsic disorder, and potential for LLPS. Numerous proteins unique to keratinocytes and other proteins with shared expression in multiple cell types were identified to have IDP traits (e.g., compositional bias, nucleic acid binding, and repeat motifs). Among keratinocyte-specific proteins, many that co-assemble with involucrin into the cell-specific structure known as the cornified envelope scored highly for intrinsic disorder and potential for LLPS. This suggests intrinsic disorder and LLPS are previously unrecognized traits for assembly of the cornified envelope, echoing the contribution of intrinsic disorder and LLPS to more widely encountered features such as stress granules and PML bodies.

## 1. Introduction

Intrinsically disordered proteins (IDPs) and intrinsically disordered regions (IDRs) in otherwise conformationally structured (ordered) proteins have limited to no fixed secondary or tertiary structure [1]. Rather than constancy in one static structure, IDPs are envisioned to move through an ensemble of conformations. That plasticity often brings with it opportunities to partner with a wide retinue of proteins through low affinity but high specificity interactions [2,3]. This varying and multi-partnered existence defines IDPs in part as hub proteins [4,5] which may be functionally fine-tuned by their transient conformation, post-translational modifications (PTMs), and/or available partners [6,7,8]. Another inherent feature of IDPs is their expected resiliency to potential extremes of intra- and extra-cellular conditions in which they find themselves—summarized previously as the “stability of instability” [9]. In addition to being hub organizing structural proteins, IDPs are also being recognized as vital participants in intracellular signaling [10,11]. In both cases, their conformational flexibility can provide access to often multiple short linear motifs for interaction with the aforementioned partners or to sites of PTMs for regulation of their stability, function, or partner association [8,12]. Thus, the cohort of a cell’s IDPs brings with it numerous advantages to the cell’s own biology and by extension the cell’s performance in a tissue [3]. Better identification of such cohorts could improve understanding of cell and tissue performance given the key roles IDPs play in both health and pathophysiology.

IDPs seem bespoke players in mammalian epidermal keratinocyte (KC) biology. Variable signals and sometimes extreme conditions at the skin surface may be more readily responded to because of IDP accessibility for PTM, polymer proteolytic processing to monomers, participation in organizational and signaling hubs, and resilience of unstructured proteins to changing environments. Skin KCs are functionally charged with forming a physically, chemically, and microbially resilient barrier to topical insults as well as preserving underlying tissue hydration via a typical ~80-micron-thick epidermis [13,14].

Within the epidermis are four histologically recognized KC layers which are morphologically, biochemically, and transcriptionally defined [13,15,16]. Epidermal KCs progress from a mitotically active basal layer of cuboidal to columnar cells directly in contact with underlying dermal tissue to a suprabasal post-mitotic spinous layer notable for numerous cell–cell desmosomal contacts enhancing tissue cohesion. Concurrent with this basal to spinous maturation step is a switch in expression of the keratin 5 and 14 pair to the keratin 1 and 10 pair. Spinous cells also initiate expression of the cornified envelope protein involucrin. These cells next develop into a granular layer distinguished by its histologically characteristic keratohyalin granules which include the filaggrin protein utilized for cytoskeletal organization. There is also new gene expression for other cornified envelope proteins. Nuclear dissolution marks the transition to fully matured KCs in the most superficial cornified layer of flattened cells (squames) filled with cross-linked proteins and surround by extruded lipids providing the classic “bricks and mortar” organization of the tissue.

Failures along this process stem from KC-specific protein mutations, expression insufficiencies, or roles as antigens in autoimmune diseases. Such protein dysfunctions serve as the molecular basis of several cutaneous pathologies including involucrin and loricrin in atopic dermatitis [17], filaggrin in ichthyosis vulgaris [18], and type XVII collagen in bullous pemphigoid [19]. We and others have previously noted each of these proteins are IDPs or have extensive IDRs [20,21,22]. To date, these connections of disorder and possible phase separation to cutaneous biology have been made by fortuitous observations that individual hallmark epidermal KC proteins possess highly prominent IDP features [20,21,22,23]. Cell-wide assessments have yet to be reported.

To comprehensively investigate IDPs in KCs, we took two complementary approaches. First, we used thermostable protein extraction to enrich for IDPs from cultured HaCaT keratinocytes. These proteins were identified by liquid chromatography tandem mass spectrometry and intrinsic disorder analysis by standardized bioinformatic approaches. Second, to broadly investigate KC proteins, we interrogated the UniProt protein database for KC-expressed proteins with follow-up bioinformatic disorder analysis. In both instances, we looked to integrate often distinct features of KC-expressed proteins with those of disorder and liquid–liquid phase separation (LLPS), especially in the KC-specific structure known as the cornified envelope (CE). This study and future analysis of KC-expressed proteins for intrinsic disorder and phase separation have the potential to significantly add to understanding of cutaneous protein function in normal physiology and epidermal disease.

## 2. Results and Discussion

### 2.1. Retention of Involucrin in a Heat Lysate of Keratinocytes

**Figure 1:**
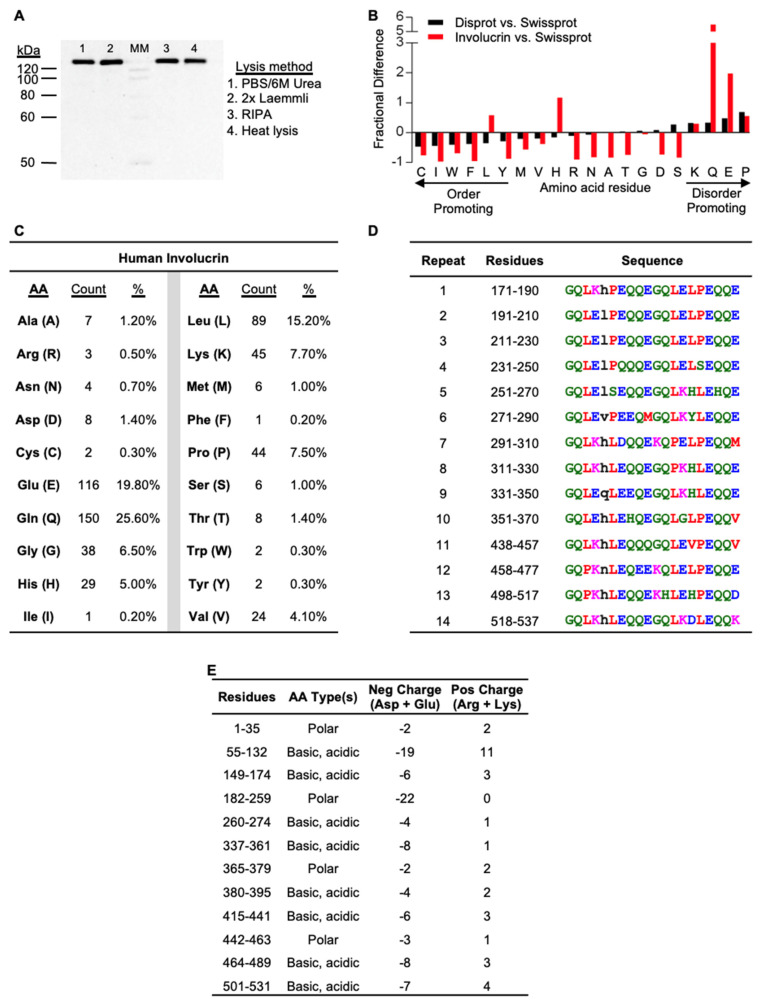
Involucrin enrichment in a thermostable protein extract and computational analysis indicate IDP characteristics. (**A**) Involucrin preferential recovery in a heat lysate; 240 ng protein per lanes 1–3, 50 ng protein lane 4. MM, MagicMark western standards. (**B**) Involucrin compositional bias compared against SwissProt ordered proteins. Note break in y-axis for Q (glutamine, Gln). (**C**) Amino acid (AA) counts and percentages for 585AA full-length human involucrin. (**D**) RADAR Repeat detection. AA color indicates shared properties. A V F P M I L W: red, small and hydrophobic; D E: blue, acidic; R K: magenta, basic; S T Y H C N G Q: green, hydroxyl, sulfhydryl, or amine. Lower case black letter AA are considered unaligned. (**E**) Regional AA enrichment.

Thermostability (TS), i.e., resistance to heat-induced denaturation and precipitation, preferentially enriches IDPs and partially depletes three-dimensionally ordered proteins [24,25,26]. TS methods for isolating IDPs recall a classic and strikingly effective heat lysis enrichment protocol for the KC-specific protein involucrin [27]. Here, involucrin recovery was compared from equal numbers of HaCaT keratinocytes lysed by three typical solubilization reagents, PBS/6M urea, 2x Laemmli sample buffer, or RIPA, versus heat lysis solubilization in buffered saline [27]. HaCaT cells were recently used for IDP studies of a late-differentiation stage KC-specific protein filaggrin [21]. When normalized to protein amount loaded per lane, PBS/6M urea, 2x Laemmli sample buffer, and RIPA extracts had 134 kDa involucrin immunoblot band intensities within ~10% of the average for those three extraction methods. Involucrin was also detected in the supernatant of the heat-treated lysate (Figure 1A) after centrifugation to remove denatured proteins.

In addition to its TS, traits separately reported supporting involucrin’s disordered characteristics include (i) gel electrophoresis and size exclusion chromatography apparent molecular weights far in excess of the 68 kDa predicted from its cDNA [28,29] and (ii) circular dichroism profile of mostly alpha helices and random coils [30]. Our involucrin immunoblot detection demonstrates SDS-PAGE migration significantly slower (Figure 1A) than the 68 kDa predicted from its cDNA. Computational assessments reported here provide new traits furthering involucrin identification as an IDP: i) amino acid content (Figure 1B,C) enriched (G, 6.5%; Q, 25.6%; K, 7.7%; E, 19.8%; P, 7.5%) versus depleted (C, 0.3%; I, 0.2%; W, 0.3%; F, 0.2%; Y, 0.3%) for residues characteristically described [10] respectively, as disorder-promoting versus order-promoting and ii) reduced sequence complexity stemming from fourteen repeats (Figure 1D) of a 20 mer motif (G Q L ^E^/_K_ ^H^/_L_ ^L^/_P_ E Q Q E G Q L ^E^/_K_ ^L^/_H_ ^P^/_L_ E Q Q ^E^/_V_) which includes the 10 mer sequence (Q E G Q L K H L E Q) previously reported [30,31]. Compositional biases are characteristic of disorder [32]; for involucrin, this includes polar and charged amino acids throughout the full-length protein (Figure 1E).

**Figure 2:**
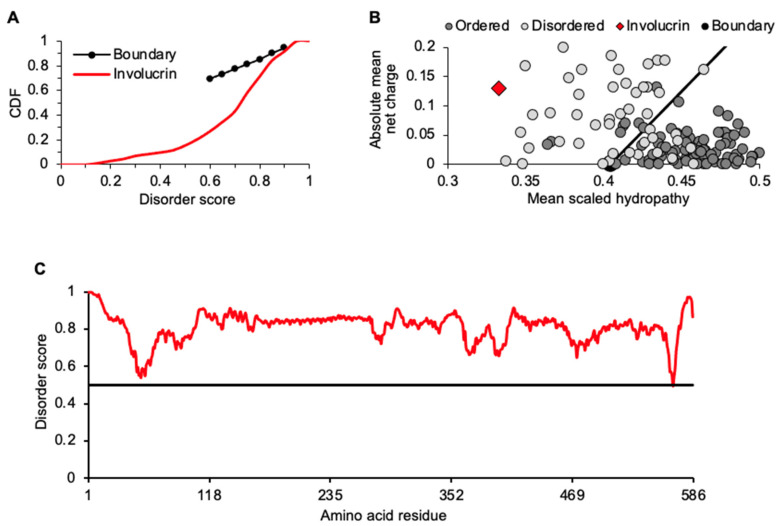
Computational analysis reports involucrin as an IDP. (**A**) Cumulative distribution function (CDF) shows high disorder scores (*x*-axis ≥0.5) reached with limited proportion of residues (*y*-axis ~0.2–0.4) positioning involucrin below a database boundary (black line). (**B**) Charge-hydropathy (CH) boundary (black line) shows separation trend for database disordered proteins (gray circles) mostly to the left and ordered proteins (dark circles) mostly to the right; involucrin (red diamond) is in disordered area. All reference values were used but to maximize distribution of points only a subset is shown. (**C**) PONDR-FIT plotted values (red line) report high disorder scores consistently above 0.5 reference value (black line) along entire involucrin protein.

Amino acid content of involucrin was further assessed with two binary predictors of disorder: a cumulative distribution function (CDF) plot of disorder per amino acid residue and a charge-hydropathy (CH) plot (Figure 2A,B). For each assessment (Figure 2A,B), involucrin clearly performs as an IDP. It has a higher mean net charge and lower mean hydrophobicity relative to ordered (structured), globular protein standards (Figure 2B). Additionally, PONDR-FIT, a meta-predictor of intrinsic disorder combining six individual algorithms [33], scores all but one (L566; 0.494) of involucrin’s 585 residues over a 0.5 with an average score across its entire length of 0.812, indicating a predominantly disordered protein (Figure 2C). Independent analyses with Espritz-D and IUPred-L within Database of Disordered Protein Predictions (D^2^P^2^) were in strong overall agreement with PONDR-FIT for disorder present throughout involucrin (Supplementary Figure S1). Additionally, numerous molecular recognition features (MoRFs) were detected (Supplementary Figure S1). This supports involucrin’s role as an early scaffold protein for oligomeric assembly of itself and binding partners such as envoplakin and periplakin proteins [34,35,36]. These three proteins and others such loricrin, for which we previously detailed intrinsic disorder [20], are assembled and ultimately crosslinked into a KC-unique structure known as the cornified envelope (CE). Intriguingly, liquid–liquid phase separation (LLPS) may be part of this KC maturational process (see below).

### 2.2. Identification and Relationships among Keratinocyte-Expressed Proteins following Thermostability Fractionation

Isolation of thermostable proteins provides for enrichment of IDPs with concurrent depletion of structured, more-globular proteins, enhancing relative recovery of IDPs for detection in downstream analyses [24,25,26,37]. Thus, with involucrin enrichment via thermostable extraction (Figure 1A) and disorder assessment (Figure 1B–E and Figure 2A–C, Supplementary Figure S1) established, other KC-expressed proteins in the thermostable lysate were identified by liquid chromatography tandem mass spectrometry (MS), examined for conceptual groupings (e.g., sequence features, gene ontology), and assessed for traits of intrinsic disorder.

**Figure 3:**
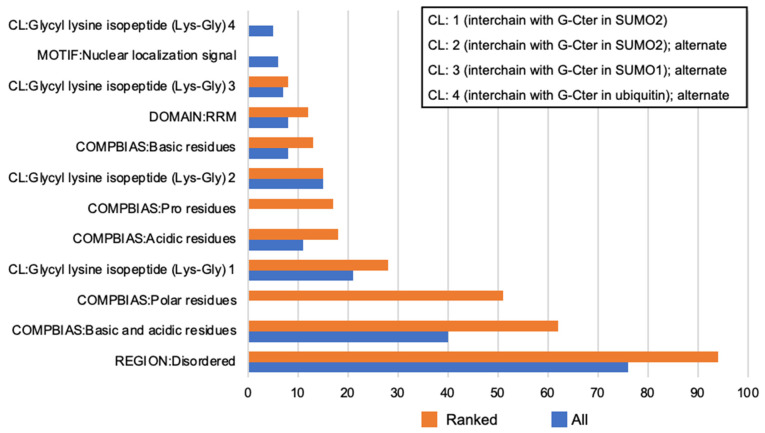
Thermostable protein GO analysis for UniProt Sequence Features. *x*-axis, percent of submitted genes mapping to *y*-axis annotation. Annotation terms are highly shared between all thermostable proteins and a subset ranked by RAPID and SLIDER. Compositional bias for polar residues was represented at 36% for all thermostable proteins at default Modified Fisher Exact (EASE score) *p* < 0.10 but not at *p* < 0.01 used for other terms. Terms returning < 5% of submitted genes are not shown. CL, CROSSLINKED. COMPBIAS: compositional bias. RRM: RNA recognition motif.

The breadth of proteins from our MS peptide identification prompted us to take a two-step approach (Figure 3) to possible ontological relationships among them: first, a “sequence feature” inquiry of the whole thermostable cohort via DAVID (Database for Annotation, Visualization and Integrated Discovery) [38] and second, repeating that inquiry with these proteins first tested for disorder by RAPID and SLIDER (Supplementary Table S1). Even without the ranking provided by RAPID and SLIDER, the thermostable cohort already showed high percentages of its proteins annotated for features in common with IDPs, e.g., disordered regions and amino acid compositional bias (Figure 3). These features increased on a percent basis (Figure 3) after ranking by RAPID and SLIDER. Among the whole thermostable set and the RAPID/SLIDER rankings, it was striking to observe the frequent crosslink annotation denoting proteins with possible post-translational modification (PTM) of sumoylation. Sumoylated targets are often nuclear proteins with that modification affecting a wide range of events involving nucleic acid binding, e.g., transcription, RNA processing, chromatin, and nuclear body assembly [39]. These events can be locally organized through LLPS aided through scaffolding provided by sumoylation [40].

**Figure 4:**
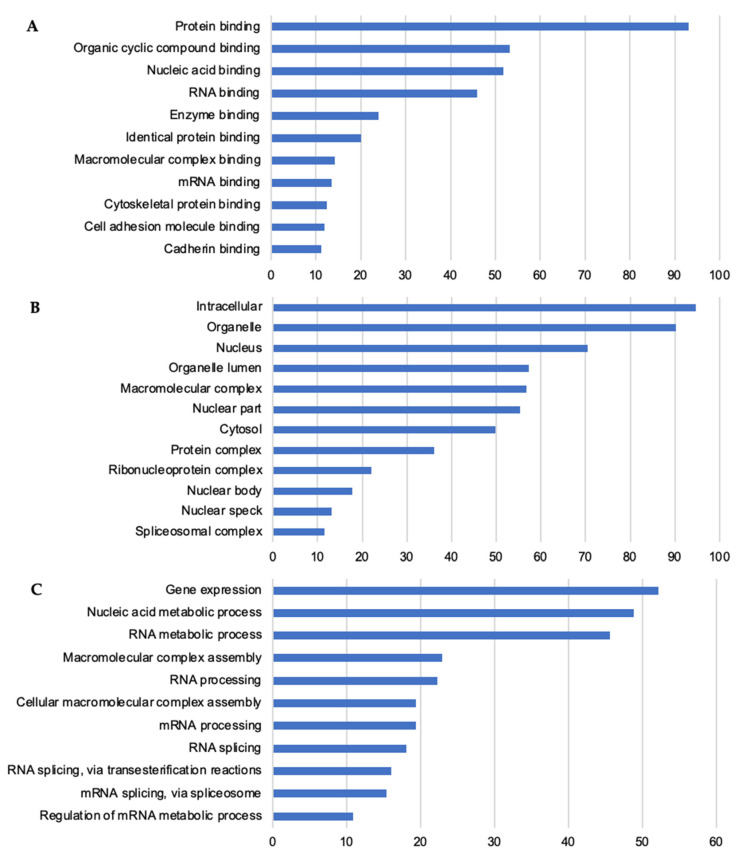
GO analysis of thermostable proteins for (**A**) Molecular Function, (**B**) Cellular Component, and (**C**) Biological Process. *x*-axis, percent of submitted genes mapping to *y*-axis annotation. All annotation terms at a false discovery rate < 0.01. Annotations terms graphed above are top 11–12 lowest Benjamini *p*-values. Overlapping terms and terms returning <10% of submitted genes not shown for space considerations. See Supplementary Table S2 for individual counts, fold-enrichment, and all *p*-values. Among Cellular Components (**B**), cornified envelope showed a 11.88-fold enrichment but number of returned genes was <10%.

Gene ontology (GO) molecular function (MF) annotation for KC thermostable lysate proteins (Figure 4A) reported binding across diverse arenas consistent with established breadth of functions involving IDPs [10]. Significant fold enrichment for numerous broadly inclusive MF terms was present. For instance, universal cell functions, e.g., RNA binding and mRNA binding were increased 4.35-fold each (*p*-value 6.13 × 10^−54^, 9.53 × 10^−13^, respectively). IDPs are frequent RNA binding proteins serving as adaptable hub proteins for assembly of large multiprotein complexes in RNA processing [41]. As to KC biology, cadherin binding was also enriched (6.51-fold; *p*-value 2.95 × 10^−15^). Cadherin proteins, such as KC desmogleins, participate in forming adherens junctions which are critical for epidermal barrier function.

Cornified envelope (GO:0001533) is the one KC-specific term occurring within cellular component GO analysis. This functionally related cohort of diverse proteins was enriched (11.88-fold; *p*-value 2.23 × 10^−6^) in the thermostable lysate (Supplementary Table S2). Other annotations within cellular component are more generic to all cell types and reflect the board distribution of thermostable proteins throughout cellular compartmental organization (Figure 4B). Involvement with nucleic acids, especially RNA, as displayed from biological process annotations (Figure 4C) is an overwhelming relationship among thermostable proteins and consistent with their being IDPs [42]. This is especially notable among inter-related terms such as regulation of mRNA splicing (via spliceosome), mRNA processing, and mRNA metabolic process showing a fold enrichment range of 14.79, 13.24, and 11.86, respectively (*p*-values 9.59 × 10^−18^, 1.04 × 10^−19^, and 3.87 × 10^−22^, respectively).

These results emphasize that KC IDPs recognized by TS are integral to many basic cellular activities as seen in other specific cell types [25,37]. Notable among this were compositional bias, potential for PTM by sumoylation (Figure 3), and frequent annotation for nucleic-acid-related events such as RNA transcription and processing (Figure 4A–C). To further these analyses, we considered individual proteins unique to or highly enriched in KCs as compared to those more broadly expressed in KCs and other cell types.

### 2.3. Intrinsic Disorder Traits of Thermostable Proteins Specific to or Highly Expressed in KCs

**Figure 5:**
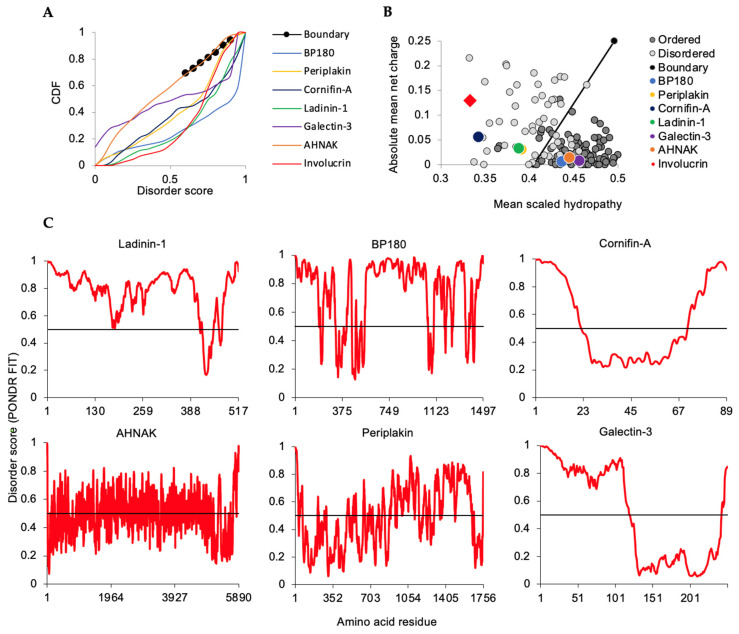
Intrinsic disorder analysis for thermostable proteins preferentially or specifically expressed in KCs. (**A**) Cumulative distribution function (CDF). Disordered proteins plot below database boundary (black line). (**B**) Charge-hydropathy boundary (black line) shows separation trend for database disordered (gray circles), ordered proteins (dark circles), and thermostable proteins. (**C**) PONDR-FIT analysis. Individual amino acids along the protein length scoring ≥0.5 reference value (black line) are in disordered regions. BP180, collagen type XVII alpha chain.

To assess intrinsic disorder and potential phase separation of representative individual thermostable proteins specific to or highly expressed in KCs, we used approaches similar to those above for involucrin: CDF and CH plots along with PONDR-FIT graphing of individual residue disorder values along the protein (Figure 5), with RAPID for percent of disorder, and SLIDER for LDR recognition. Proteins called out here highlight established KC contributions to the epidermal barrier such as cell–cell and cell-extracellular matrix attachments (ladinin-1, BP180, AHNAK, galectin-3), in [43,44,45] cytoskeletal organization (AHNAK, periplakin) [35,44,46] and CE formation (involucrin, cornifins, perplakin) [16,35,46,47,48] (Table 1). Overall, these IDPs or proteins with IDRs are involved in functions critical to KCs as well as participating in more generic roles as revealed by the GO analysis above.

As for involucrin (Figure 2A,B), thermostable lysate proteins cornifin A, ladinin, and periplakin (Figure 5A,B) mapped to disordered regions of CDF and CH plots. Like some disordered CH database standards [49], AHNAK/desmoyokin, galectin, and collagen type XVII alpha chain, alias BP180, were over the trend line (Figure 5B). Nevertheless, each has features which support TS enrichment and are consistent with being an IDP or having extended IDRs (Table 1). AHNAK/desmoyokin possesses structure-breaking proline-richness (12.2%) and extensive motif repeats [50]. BP180 has an unstructured intracellular domain [22] and extensive disorder in its extracellular collagenous domain (Figure 5C, AA565–1050 and AA1104–1355) as we earlier reported [51]. We detected both galectin-3 and -7 in the thermostable KC lysate (Supplementary Table S1). Although not yet directly studied for galectin-7, galectin-3 has been biophysically characterized as intrinsically disordered at its amino terminus [52] and to participate in LLPS [53] encouraging us to carry out an initial assessment of phase separation potential for other proteins in Table 1. While neither galectin-3 nor -7 are wholly unstructured proteins, it may be that their proportional extent of disorder, relatively localized to their amino termini (e.g., galectin-3, Figure 5C), is sufficient for TS retrieval but not localization to extensive disorder on CH plots (Figure 5B).

It is important to note that proteins with negative catGRANULE scores (Table 1) such as the extremely compositionally biased cornifin A and cornifin B (each with glutamine ≥ 18% and proline ≥ 29%) are not by that score necessarily excluded from participation in or regulation of LLPS. Rather, they do not score favorably for phase separation by themselves alone. For instance, granulin 5 is an extremely compositionally biased 54 amino acid peptide, with a catGRANULE propensity score −2.83, proteolytically derived from the 63.5 kDa progranulin protein [54]. Nevertheless, it can initiate phase separation of the neurodegeneration-associated TAR DNA-binding protein 43 (TDP-43) [54].

Representative thermostable proteins specific to or highly expressed in KCs covered a range of PONDR-FIT average scores (Table 1). For involucrin (Figure 2C), AHNAK/desmoyokin, ladinin, periplakin, and BP180 proteins (Figure 5A), PONDR-FIT plots reported scores ≥0.500 for hundreds of contiguous amino acid residues indicating extensive lengths of disorder in these proteins (Table 1, RAPID and SLIDER scores). Several of these proteins (e.g., involucrin, cornifins, and periplakin) are involved in ultimate formation of the KC-unique structure known as the cornified envelope and will be further considered below. Others such as ladinin are as yet largely uncharacterized but implicated in intracellular cytoskeletal roles in epithelial cells [55] and possibly other intra- and extra-cellular roles [56]. The latter includes attachment to the extracellular matrix where conformational flexibility may aid functional diversity [57]. We have previously detailed potential disorder advantages conferred on the integral membrane BP180 [51]. In brief, the combination of its unstructured intracellular [22] and extracellular domains (Figure 5C) may provide flexibility that is at least compatible with interaction of other transmembrane proteins involved in generation of the KC hemidesmosome for attachment to the underlying matrix. Thus, intrinsic disorder, as assessed for these proteins unique to or preferentially expressed in KCs, is likely to add to elements specific to these cells, e.g., the CE, or to KCs performing generalizable functions with specialized proteins, e.g., attachment to the extracellular matrix.

### 2.4. Intrinsic Disorder Traits of Broadly Expressed Proteins Retrieved in the KC Thermostable Lysate

**Figure 6:**
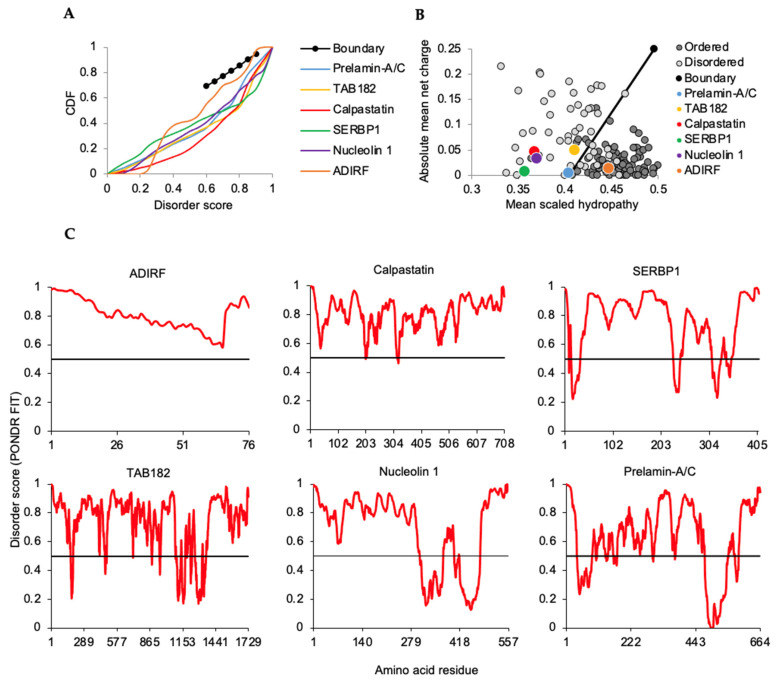
Intrinsic disorder analysis of broadly expressed proteins retrieved in KC thermostable lysate. (**A**) Cumulative distribution function (CDF). (**B**) Charge-hydropathy boundary (black line) shows separation trend for database disordered with thermostable proteins overlapping reference set disorder. (**C**) PONDR-FIT analysis. *y*-axis values ≥0.5 reference value (black line) are in disordered regions. ADIRF, adipogenesis regulatory factor. SERBP1, plasminogen activator inhibitor 1 RNA-binding protein. TAB182, 182 kDa tankyrase-1-binding protein.

Representative thermostable proteins with more-ubiquitous expression were also present in the HaCaT keratinocyte lysate (Figure 6; Table 2). These proteins plotted within regions characteristic of or overlapping disordered reference points for CDF and CH analyses (Figure 6A,B). Adipogenesis regulatory factor (ADIRF, PONDR-FIT score 0.810) detection appears to be novel for expression in KCs and prediction of intrinsic disorder. Calpastatin, plasminogen activator inhibitor 1 RNA-binding protein (SERBP1), 182 kDa tankyrase-1-binding protein (TAB182), nucleolin, and prelamin A/C mouse homologues were previously found in a 3T3 thermostable lysate [24]. PONDR-FIT scores derived from averages across corresponding plots (Figure 6C) generally correlated with those for RAPID and SLIDER for these proteins (Table 2). There was a favorable, although not universal, trend with predicted phase separation which may reflect limitations of using just one LLPS assessor [58,59,60] which we addressed for CE proteins below.

Most of the proteins highlighted here recall the extensive representation of thermostable IDPs associated with gene expression, nucleic acid binding, and RNA processing (Figure 4). In contrast, as a specific inhibitor of calpain proteases, calpastatin highlights a different regulatory role for IDPs. Although broadly expressed, calpastatin loss of function is almost exclusively associated with a skin pathology phenotype (Online Mendelian Inheritance in Man *114090; #616295) with excessive epidermal thickening and peeling [61,62]. Bioinformatic assessment here of calpastatin (Figure 6) provides distinct data to corroborate biophysical studies of its unstructured characteristics where flexibility is considered key to its interaction with calpain enzymes [63,64]. This suggests that without the inhibitory benefit of the IDP calpastatin, the structural organization of stratified KCs is distinctly sensitive to unregulated calpain activity.

### 2.5. Keratinocyte Proteins: Database Intrinsic Disorder Assessment

We extended intrinsic disorder analyses to UniProt database proteins identified from KCs to expand examination to late and fully matured KCs which may have been under-represented in these cultured cells. Some KC-specific proteins well characterized as to differentiation-dependent expression and role in cell maturation from this UniProt set did not meet the standard SLIDER cutoff of 0.538 and yet scored with a relatively high RAPID percent disorder. These typically small proteins (<150 AA) often have regions of disorder approaching, but not meeting, the ≥30 contiguous residues or other compositional expectations of SLIDER [65]. Examples include small proline-rich protein 2E and 2D, (P22531 and P22532, respectively), SLIDER, 0; RAPID, 59.72% each (Supplementary Table S3). Thus, only RAPID was used and proteins ranked ≥25% disordered were subsequently assessed with DAVID (Supplementary Figure S2). GO analysis reported significant enrichment for sequence features such as amino acid compositional bias for polar and basic-acidic residues. Numerous molecular function terms reflecting nucleic acid binding including “transcription regulatory region sequence-specific” were also enriched (Supplementary Figure S2) recalling as with the thermostable proteins that gene expression regulatory factors are highly represented among IDPs [66,67].

UniProt hits (Supplementary Table S3) provided late KC maturation markers such as loricrin and filaggrin. Their RAPID and SLIDER analyses, (loricrin: 62.82%, 0.665; filaggrin: 55.58%, 0.939) agree with other intrinsic disorder traits we recently assessed in silico for these terminal differentiation proteins [20]. Extensive biophysical analysis of filaggrin by Quiroz et al. [21,68] has shown its ability to undergo LLPS and that mutant filaggrin proteins that have lost this trait are associated with skin pathology.

The CE protein involucrin is the prototype for KC-specific thermostable IDPs and has LLPS potential as assessed with catGRANULE. Additionally, “cornified envelope” is the most significantly enriched term (48.90-fold; *p*-value, 4.17 × 10^−26^; FDR, 3.74 × 10^−26^) among the cellular component ontology terms within the UniProt KC hits. Thus, we used CE as a focus for further assessment of KC proteins for disorder and phase separation potential.

### 2.6. KC Cornified Envelope (CE) Components Are Candidates for LLPS

The cornified envelope is a sheath (Latin involucrum) of cross-linked proteins organized under and eventually replacing the cell membrane during late steps of KC differentiation ([16] for review). This ultimately results in a physically resilient insoluble structure [34] at the skin surface. Involucrin, periplakin, and envoplakin are among early CE precursors providing scaffolding and deposition sites for later assembled proteins such as filaggrin, loricrin and elafin [69,70]. Scaffold proteins involved in phase separation may not be enzymatically active but nevertheless can improve the efficiency of events associated with them, such as decreasing response time for assembly of relevant partner proteins, often due to the repeat motifs within them [71,72,73]. Several other non-keratin proteins, e.g., cystatin A and small proline-rich (SPR) proteins, can also contribute to CEs [74,75].

**Figure 7:**
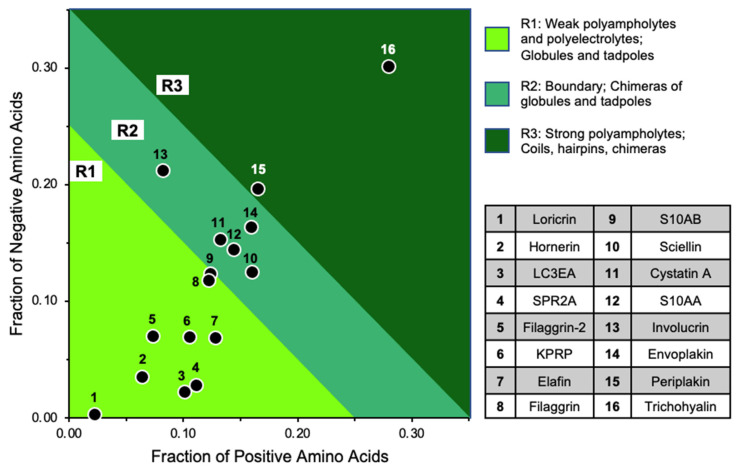
CIDER analysis of CE proteins. Das–Pappu diagram of states depicting proposed structural conformation for each protein based on the fraction of positive and negative charged residues. Plot regions and protein names in legends at right.

Given amount, distribution, and pattern of charged and hydrophobic residues within disordered proteins can affect biophysical behaviors such as compaction, we first examined CE proteins for biophysical traits with CIDER (Classification of Intrinsically Disordered Ensemble Regions) [76,77]. The resulting diagram of states (Figure 7) shows CE proteins almost exclusively populating regions 1 and 2. Notably, among most of these CE proteins and despite their extreme differences in length, there appears to be well balanced increases in positive and negative charges maintaining a relatively low net charge per residue (Table 3). Those charges, especially among earlier components (e.g., involucrin, periplakin, and envoplakin) are relatively well-mixed (lower kappa values). The parameter of charge mixing versus separation in the context of proline distribution (omega) [78] can also affect protein expansion versus compaction especially for instances of low charge and highly localized proline residues. Three CE proteins stand out at omega > 0.5; loricrin, filaggrin 2, and hornerin (Table 3, Figure 7). Although each is under three percent proline content, they are KC proteins with repeat sequences making up a vast extended length of the protein which results in some residues being highly isolated. Repeats in several of these CE proteins [34,79,80] such as involucrin (Figure 1D) are common characteristics of biomolecular condensates featuring multivalency [73,81].

Condensation of CE precursors could provide increases in local concentrations of proteins facilitating their interaction and assembly as is often the case for other IDP with characterized tendencies to phase separate [71,72,73]. As such, we extended CE protein analysis for their potential to undergo LLPS. In this context, intrinsic disorder is not a strict one-to-one assurance of LLPS [42,60]. Other factors such as type and frequency of side chain interaction can influence LLPS. Thus, we analyzed CE proteins for a summary of intrinsic disorder and potential for LLPS, the latter by separate algorithms based on different criteria [58,59,60,82].

Possibly contributing to their CE functional relationship, several of these highly specialized proteins, such as involucrin and loricrin, did show a trend of disorder with potential to phase separate that held across different algorithms (Table 4). Extending previous biochemical analysis [21], filaggrin scored consistently high across the multiple phase separation platforms. The utility of multiple phase separation algorithms became evident for some small and larger CE proteins with extreme compositional bias or motif repeats, e.g., small proline-rich 2A (SPR2A: AA ≥ 10%: cysteine, 15.3; glutamine,16.7; lysine, 11.1; proline, 37.5) and keratinocyte proline-rich protein (KPRP: AA ≥ 10%: glutamine, 11.1; proline, 18.0; serine, 11.1), which may affect their performance on some platforms. CE proteins with lower innate phase separation potential may be “client” proteins [83], compatible and colocalizing with those more inherently likely to drive LLPS either in their native form or after post-translational modifications.

### 2.7. Conclusions

TS is an established means of IDP enrichment [24,25,26,37] but to our knowledge has not been broadly used for KC. One KC-specific protein, involucrin, was previously isolated via its TS [27]. It served as our prompt for investigation of other thermostable KC proteins for disordered characteristics and LLPS potential. Involucrin’s role in CE formation was also an entry point for examination of other CE proteins as to their intrinsic disorder and potential for phase separation.

As previously reported, triple knockout of the CE scaffolding proteins involucrin, periplakin, and envoplakin is required for loss of epidermal barrier function; individual knockouts of these proteins have only very subtle and eventually resolving effects on epidermal KC function [84]. Their apparent operational overlap may stem from functional redundancy. Notably, while these proteins do not share high protein amino acid sequence identity, they have in common scaffolding abilities, intrinsic disorder, and phase separation traits where one might compensate for the other in CE protein association. Notably, coincident with CE protein assembly are dramatic changes in the KC cytoplasmic environment including changes in redox potential, pH, and calcium gradients [68]. Such conditions have been intimately linked to LLPS [85,86,87].

Many KC thermostable proteins sorted to GO cohorts such nucleic acid binding, a common characteristic of IDPs. Additionally, we established a bridge between the GO annotation term “cornified envelope” and intrinsic disorder in that many proteins involved in CE formation are enriched for biophysical traits of conformationally flexible proteins (Table 3 and Table 4). Further, many of these proteins have significant potential for phase separation suggesting a previously unrecognized mechanism for CE protein organization.

Several CE proteins assessed here (including involucrin, loricrin, small proline-rich proteins, and filaggrin 1 and 2) are encoded by a sequential arrangement of these and other KC maturation-related genes referred to as the “epidermal differentiation complex” (human chromosome 1q21). The CE emphasis from this work and the KC differentiation-dependent proteins we previously examined from the cluster [20] suggest intrinsic disorder and phase separation are important aspects of global KC maturation. Traits of intrinsic disorder and potential for phase separation shared among many CE proteins, especially those of unrelated amino acid sequence, greatly extends the impact such biophysical characteristics may have on KC biology. This influence was previously recognized only for specific, individual proteins, e.g., hornerin, BP180, and filaggrin [21,22,23].

The IDPs identified here from the thermostable lysate and database investigations illustrate potential intrinsic disorder and phase separation contributions to incredibly diverse KC functions, e.g., basement membrane attachment (ladinin, BP180) and CE scaffolding (involucrin, loricrin) along with more general cellular events such as enzyme inhibition (calpastatin) and RNA-binding (nucleolin). Together, these results support the broad involvement of IDPs and phase separation in KC biology.

## 3. Materials and Methods

### 3.1. Cell Culture

HaCaT keratinocytes were maintained as described [88]. Cultures for protein extraction were seeded at 1.03 × 10^6^ cells per 10 cm diameter plate, grown until confluent, maintained for three additional days post-confluence, and then changed to serum-free media for one more day before harvesting. These conditions provide for early and mid-differentiation characteristics and assure involucrin expression [89,90]. HaCaT use in diverse KC studies including for filaggrin IDP phase separation has been previously described [21].

### 3.2. Protein Preparation and Analysis

Four buffers were used for lysis of equal numbers of cells: RIPA buffer, 10 mM Tris, 150 mM NaCl, 1 % deoxycholic acid, 1 % Triton and 0.1 % sodium dodecyl sulfate; PBS/6M urea; 2× Laemmli sample buffer, 4% SDS, 20% glycerol, 120 mM TrisHCL (pH 6.8); or Etoh-Green buffer for heat lysis, 1× PBS, 20 mM EDTA, 62.5 mM Tris HCl (pH 6.8) and 10% glycerol [27]. In the cases of RIPA, PBS/6M urea and 2× Laemmli, cells were lysed with continuous rocking for 30 min. All lysates contained protease inhibitor cocktail (Roche Life Science, Indianapolis, IN, USA) and protein phosphatase inhibitors (2 mM sodium orthovanadate and 50 mM sodium fluoride). For heat lysis using the Etoh-Green method, cells were scraped from plates, pelleted, resuspended in Etoh-Green buffer, and heated at 99 °C for 20 min. Heat lysates were then chilled on ice for 10 min. For all lysates, cell debris was pelleted at 10,000× *g* for 30 min. Protein quantification was performed using Pierce 660 reagent (Thermo Fisher Scientific, Waltham, MA, USA). Gel electrophoresis, Western blotting, and band densitometry were performed as previously described [91]. For Western blot detection of involucrin, mouse monoclonal anti-involucrin antibody (SAB4200794, 1:1000 dilution, MilliporeSigma, Burlington, MA, USA) was used. Involucrin apparent molecular weight was determined with immuno-detected Thermo Fisher MagicMark standards.

### 3.3. Liquid Chromatography (LC) Tandem Mass Spectrometry (MS, LC MS/MS)

Thermostable lysate proteins were adjusted to pH 8 using ammonium hydroxide and all Cys residues were subject to reduction and subsequent alkylation using 5 mM dithiothreitol in 0.1 M ammonium bicarbonate and 10 mM iodoacetamide in 0.1 M ammonium bicarbonate, respectively. Proteins were digested with sequencing grade modified trypsin (Promega, Madison, WI, USA) at a 1:20 enzyme:protein ratio for 16 h at 37 °C with constant shaking. The digestion was quenched by adding concentrated formic acid to yield pH 2.5. Proteolyzed peptides were desalted using Pierce C18 Peptide Desalting Spin Columns per manufacturer’s instructions.

Peptides were subjected to mass analysis using a Thermo Scientific Q Exactive HF mass spectrometer directly coupled to a Thermo Scientific Ultimate 3000 RSLCnano ultra-high performance liquid chromatograph. Peptides were initially loaded onto a 25 cm Waters BEH analytical column and gradient-eluted using a 60 min linear, reversed phase separation. Peptides were ionized directly into the Q Exactive HF using nanoelectrospray ionization and mass analyzed using a Top15 data-dependent acquisition method. Peptide and protein identification and quantification was performed using MaxQuant software suite (v1.6.0.1, Max Planck Institute for Biochemistry, Martinsried, Germany) [92] and embedded Andromeda search engine against full Uniprot Homo sapiens reference proteome (UP000005640, database downloaded 17 April 2017). Search parameters included a 4.5 and 20 ppm mass tolerance for precursor and fragment ions, respectively, a minimum of 5 amino acids/peptide, fixed carbamidomethyl Cys, and the following variable modifications: oxidation of Met, protein N-terminal acetylation, and peptide N-terminal Gln to pyro-Glu conversion. Remaining parameters were left at default values. All results were filtered to a 1% false discovery rate at the protein and peptide-spectrum-match levels using a decoy database search and consequently uploaded into Scaffold Q+S Version 4 (Proteome Software, Inc., Portland, OR, USA) for data visualization and further analysis. Within each lysate’s results, we also applied a two-unique peptide/protein threshold as is common for such analyses [93]. Proteins in Supplementary Table S1 passed this threshold for one or both independent lysate samples (See Supplementary Table S1 for Total Spectrum Counts and Average Precursor Intensities). Across the two lysates from independently grown sets of cultures, the thermostable proteome of the second set identified 517 of the 528 proteins found in the first preparation providing for an extensive cohort of shared proteins across the two samples. Protein isolation based on TS enriches for IDP in general; it is not necessarily a strict linear relationship of greater heat resistance and greater disorder [24,94]. Our approach was consistent with such precedents.

### 3.4. Bioinformatics: Intrinsic Disorder, Sequence Retrieval, and Phase Separation Analyses

Disorder assessments, plot standards, and boundary lines for PONDR, PONDR-FIT, CDF, and CH plots have been described [33,49,91,95,96]. PONDR-FIT scores averaged across a protein provided for a global indication of disorder as described in analysis of thermostable proteins [24]. Protparam (Protein Parameters) https://web.expasy.org/protparam/ (accessed on 1 September 2022), D^2^P^2^ (Database of Disordered Protein Predictions) http://d2p2.pro/ (accessed on 15 June 2022), and RADAR (Rapid Automatic Detection and Alignment of Repeats) https://www.ebi.ac.uk/Tools/pfa/radar/ (accessed on 1 July 2022), analyses were conducted with default settings [97,98]. The Das-Pappu diagram of states and accompanying values were derived at http://pappulab.wustl.edu/CIDER/analysis/ (accessed on 31 August 2022), [76].

MS identified 517 proteins in common (see Supplementary Table S1 for MS data) to the repeat sets of lysates which were submitted to Regression-based Accurate Predictor of Intrinsic Disorder (RAPID) and Super-fast predictor of proteins with Long Intrinsically DisordERed regions (SLIDER) [65,99], both available at http://biomine.cs.vcu.edu/ (accessed on 4 November 2022). GO assessment with DAVID at https://david.ncifcrf.gov/ (accessed on 14 September 2022), was performed with all 517 and then 315 ranked proteins that scored working cutoffs of ≥25% for disordered residue content via RAPID and ≥0.538 via SLIDER (see Figure 3 for 517 “All” and 315 “Ranked”) as per previously established biocomputational assessments of thermostable protein disorder [25,26,37]. All *p*-values called out in text are the Benjamini adjusted *p*-value. DAVID v2022q2 with Entrez Gene (3 June 2022) and UniProt (https://www.uniprot.org/; release 2022_02, published on 25 May 2022) resources were accessed on 25 June 2022 [38]. The default *p*-value EASE score, (modified Fisher Exact *p*-value for gene-enrichment analysis) option of 0.10 was made stricter at 0.01. Proteins were retrieved from the UniProt database using search string “(keratinocyte OR keratinocytes) AND reviewed: yes AND organism: ‘Homo sapiens’”. UniProt hits were dependent on the site-reviewed entry having the keratinocyte term. Thus, the thermostable lysate and our subsequent analysis (Supplementary Table S1) contains some candidate IDPs in addition to that database approach. Supplementary Table S1 proteins are identified as per UniProt IDs as returned from MS Scaffold.

Proteins in Table 3 and Table 4 are based on the CE literature reporting individual proteins and CE-wide components [16,29,34]. Multiple phase separator predictors were used given their different criteria and training sets [58,59,60]. Human proteome values for catGRANULE range from a minimum of − 8.434 to a maximum of +7.808 as +/− the number of standard deviations away from the training set proteome mean [58,100]. Scores >0 are favorable for phase separation with scores > 1 identifying proteins with high confidence [60,100]. FuzDrop: human proteome scores range 0.06–1.00 with predicted threshold for droplet formation ≥ 0.60 [101]. For PScore, likelihood of phase separation increases with positive scores from 1.0 standard deviation above the database set to a confidence threshold of ≥4.0 [102]. DeePhase sets criteria of a phase-separating protein at a score of >0.5 output value [103]. PSPredictor thresholds were described previously [82]. Phase separation potential was calculated with online algorithms as follows: catGRANULE [100] http://service.tartaglialab.com/ (accessed on 4 September 2022); FuzDrop [101] https://fuzdrop.bio.unipd.it/predictor; PScore [102] http://abragam.med.utoronto.ca/~JFKlab/Software/psp.htm (accessed on 10 September 2022); DeePhase [104] https://deephase.ch.cam.ac.uk/ (accessed on 10 September 2022); and PSPredictor [82] http://www.pkumdl.cn:8000/PSPredictor/ (accessed on 11 June 2022).

## Figures and Tables

**Table 1:** Computational analysis for a subset of thermostable proteins specific to or highly expressed in HaCaT epidermal keratinocytes (see also Figure 5).

Protein	UniProt #	AA	Protein Role	PONDR-FIT (0–1)	RAPID Disorder %	SLIDER IDR (0–1)	catGRAN ^a^
Involucrin ^b^	P07476	585	CE component, cornification ^c^	0.812	65.81	0.917	0.821
Ladinin-1	O00515	517	ECM and cadherin binding	0.774	68.28	0.929	0.333
BP180	Q9UMD9	1497	Transmembrane attachment to ECM	0.736	40.75	0.913	3.184
Cornifin A ^d^	P35321	89	CE component, cornification	0.551	79.78	0.848	−2.527
AHNAK/Desmoyokin ^d^	Q09666	5890	Scaffolding, nuclear-cytoplasmic shuttling	0.494	48.37	0.851	2.942
Periplakin	O60437	1756	CE component, scaffolding	0.491	30.30	0.903	0.857
Galectin-3 ^d^	P17931	250	Cell–cell, cell–ECM interaction, splicing	0.501	18.00	0.487	1.571

^a^ catGRAN, catGRANULE Propensity, for relative scoring, see Section 3. ^b^ Involucrin: known thermostable protein included for comparison to those newly reported here. ^c^ Cornification: inclusive term for keratinocyte-specific, differentiation-dependent events. ^d^ Cornifin B, AHNAK 2, and galectin-7, are uniquely encoded and were also in TS lysate. See Supplementary Table S1. AA, amino acids. CE, cornified envelope. ECM, extra-cellular matrix.

**Table 2:** Computational analysis for a subset of HaCaT keratinocyte-expressed thermostable proteins also found widely expressed in other cell types (see also Figure 6). Columns as in Table 1.

Protein	UniProt #	AA	Protein Role	PONDR-FIT (0–1)	RAPID Disorder %	SLIDER IDR (0–1)	catGRAN ^a^
Prelamin A/C	P02545	664	Nuclear membrane support, scaffolding	0.620	34.79	0.9015	0.904
TAB182	Q9C0C2	1729	Nuclear and cyto-plasmic RNA-binding	0.734	51.13	0.9281	1.975
Calpastatin	P20810	708	Calpain protease inhibitor	0.804	78.39	0.9565	1.010
SERBP1	Q8NC51	408	mRNA stability, PML formation	0.751	60.54	0.9049	2.829
Nucleolin 1	P19338	710	RNA-binding, pre-rRNA transcription	0.633	62.39	0.9357	2.495
ADIRF	Q15847	76	Transcription factor	0.810	50.00	0.6675	−0.330

^a^ catGRAN, catGRANULE Propensity, for relative scoring, see Section 3. AA, amino acids. TAB182, 182 kDa tankyrase-1-binding protein. SERBP1, SERPINE1 mRNA-binding protein 1 (alias, Plasminogen activator inhibitor 1 RNA-binding protein). ADIRF, adipogenesis regulatory factor.

**Table 3:** Classification of intrinsically disordered ensemble regions (CIDER) physicochemical characterization of CE proteins. Lengths vary greatly among CE components. Proteins sorted for increasing FCR.

Protein	UniProt #	AA	Kappa	Omega	FCR	NCPR	Hydropathy	Dis. Prom.	PR
Loricrin	P23490	312	0.252	1.787	0.026	0.019	4.244	0.827	1
Hornerin	Q86YZ3	2850	0.162	0.823	0.099	0.029	3.136	0.911	1
LC3EA	Q5TA76	89	0.309	0.420	0.124	0.079	3.602	0.775	1
SPR2A ^a^	P35326	72	0.151	0.115	0.139	0.083	3.193	0.764	1
Filaggrin 2	Q5D862	2391	0.227	0.621	0.144	0.003	3.134	0.883	1
KPRP ^a^	Q5T749	579	0.185	0.172	0.174	0.036	3.711	0.710	1
Elafin	P19957	117	0.124	0.227	0.197	0.060	4.664	0.598	1
Filaggrin	P20930	4061	0.164	0.235	0.240	0.005	2.827	0.919	1
S10AB	P31949	105	0.149	0.213	0.248	0	4.160	0.619	1
Sciellin	O95171	688	0.143	0.134	0.285	0.035	3.455	0.657	2
Cystatin A	P01040	98	0.146	0.146	0.286	−0.020	3.822	0.633	2
S10AA	P60903	97	0.098	0.231	0.289	0	4.139	0.598	2
Involucrin	P07476	585	0.144	0.060	0.294	−0.130	2.996	0.776	2
Envoplakin	Q92817	2033	0.129	0.116	0.323	−0.004	3.700	0.716	2
Periplakin	O60437	1756	0.138	0.086	0.362	−0.031	3.518	0.695	3
Trichohyalin	Q07283	1943	0.116	0.110	0.581	−0.021	1.995	0.838	3

^a^ Fraction of proline high at >0.15: SPR2A, 0.38; KPRP, 0.18. CE, cornified envelope. AA, amino acids. FCR, fraction of charged residues. NCPR, net charge per residue. Dis. Prom., disorder promoting. PR, CIDER plot region, see Figure 7. SPR2A, Small proline-rich protein 2A. LC3EA, Late cornified envelope protein 3A. KPRP, Keratinocyte proline-rich protein.

**Table 4:** Computational assessment of CE proteins for intrinsic disorder contrasted to potential to phase separate. Proteins sorted for decreasing PONDR-FIT score.

Protein	UniProt #	AA	PONDR-FIT (0–1)	RAPID Disorder %	catGRAN. ^a^	FuzDrop pLLPS (0–1) ^b^	P Score ^c^	DeePhase ^d^	PSPred. ^d^
Hornerin	Q86YZ3	2850	0.923	57.72	5.572	1.000	11.91	0.88	0.9527
Filaggrin 2	Q5D862	2391	0.896	49.14	4.418	0.9999	12.03	0.87	0.9904
Filaggrin	P20930	4061	0.895	55.58	3.553	1.000	4.73	0.83	0.9908
Loricrin	P23490	312	0.840	62.82	7.808	0.9985	12.31	0.81	0.9958
Involucrin	P07476	585	0.812	65.81	0.821	0.9921	2.18	0.78	0.4945
Trichohyalin	Q07283	1943	0.753	73.60	1.312	0.9947	0.66	0.72	0.2190
LCE3A	Q5TA76	89	0.625	18.18	−0.779	0.9978	NA	0.59	0.9596
Sciellin	O95171	688	0.620	41.57	1.306	0.9527	2.10	0.78	0.9546
KPRP	Q5T749	579	0.571	29.02	−0.591	0.9867	5.72	0.82	0.9096
SPR2A	P35326	72	0.530	65.28	−3.342	0.9868	NA	0.58	0.7971
Periplakin	O60437	1756	0.491	30.30	0.857	0.6168	0.38	0.59	0.2852
Envoplakin	Q92817	2033	0.478	26.41	0.780	0.8022	2.00	0.81	0.6487
S100-A11	P31949	105	0.374	17.14	−0.481	0.1275	NA	0.11	0.0125
Cystatin A	P01040	98	0.363	27.55	0.410	0.1673	NA	0.10	0.0050
Elafin	P19957	117	0.350	30.77	−0.180	0.2376	NA	0.17	0.0574
S100-A10	P60903	97	0.336	16.49	−0.373	0.2124	NA	0.08	0.0033

^a^ catGRAN, catGRANULE Propensity. ^b^ pLLPS, probability of spontaneous liquid–liquid phase separation. Human proteome ranges 0.06–1.00 with predicted threshold for droplet formation ≥ 0.60. ^c^ PScore only accepts sequences > 140 AA. NA, scoring not available. ^d^ DeePhase and PSPredictor (PSPred.), also output Yes or No binary descriptor (not shown) for values ≥ 0.5 or smaller, respectively. ^a–d^ For relative scoring, see Section 3. Different algorithms report scores on different number ranges; the extent of numerical difference between scores from different evaluators are not directly comparable. Abbreviations as in Table 3.

## Data Availability

Data are contained within the article or supplementary material.

## Supplementary Materials

The following supporting information can be downloaded at: https://www.mdpi.com/article/10.3390/ijms232214323/s1.
