# Supplementary material for: Thermostable Proteins from HaCaT Keratinocytes Identify a Wide Breadth of Intrinsically Disordered Proteins and Candidates for Liquid–Liquid Phase Separation
Source: Int J Mol Sci. 2022 Nov 18;23(22):14323. doi: 10.3390/ijms232214323 (PMC9692912; doi:10.3390/ijms232214323)
Supplement: Supplementary file 1 [file ijms-23-14323-s001.zip › Supplementary Figure S1.pdf]

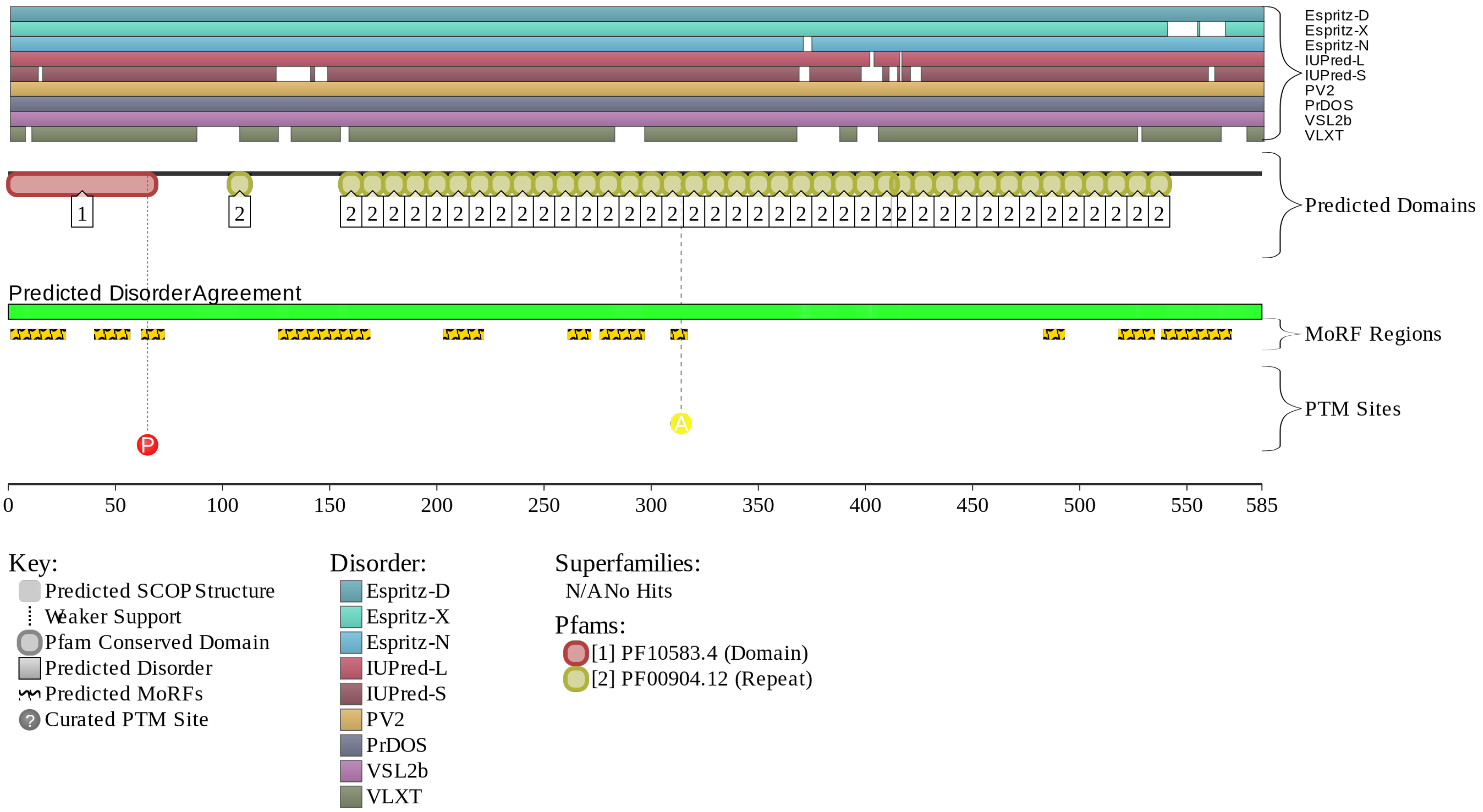

Supplementary Figure S1. D2P2 computational analysis of involucrin amino acid sequence. Results of nine disorder predictors (colored bars, see Disorder key) are aligned. Predicted domains: PF10583.4, involucrin N-terminus, residues 1-69 conserved across species and without glutamine-rich repeats; PF00904.12, characteristic of the rest of protein. Consensus across disorder algorithms is summarized by intensity of green in Predicted Disorder Agreement bar representing entire protein. Stippled yellow bars under Protein Disorder Agreement show location of predicted binding sites, molecular recognition features (MoRFs). PTM sites for phosphorylation (red) and acetylation (yellow) are indicated with colored circles extending from predicted domain line.
