# Supplementary material for: Thermostable Proteins from HaCaT Keratinocytes Identify a Wide Breadth of Intrinsically Disordered Proteins and Candidates for Liquid–Liquid Phase Separation
Source: Int J Mol Sci. 2022 Nov 18;23(22):14323. doi: 10.3390/ijms232214323 (PMC9692912; doi:10.3390/ijms232214323)
Supplement: Supplementary file 1 [file ijms-23-14323-s001.zip › Supplementary Figure S2.pdf]

Supplementary Figure S2. GO analysis of UniProt KC hits post RAPID ranking for Biological Process, Cellular Component, and Molecular Function. All terms are as per DAVID GO. See main text Materials and Methods for threshold levels.

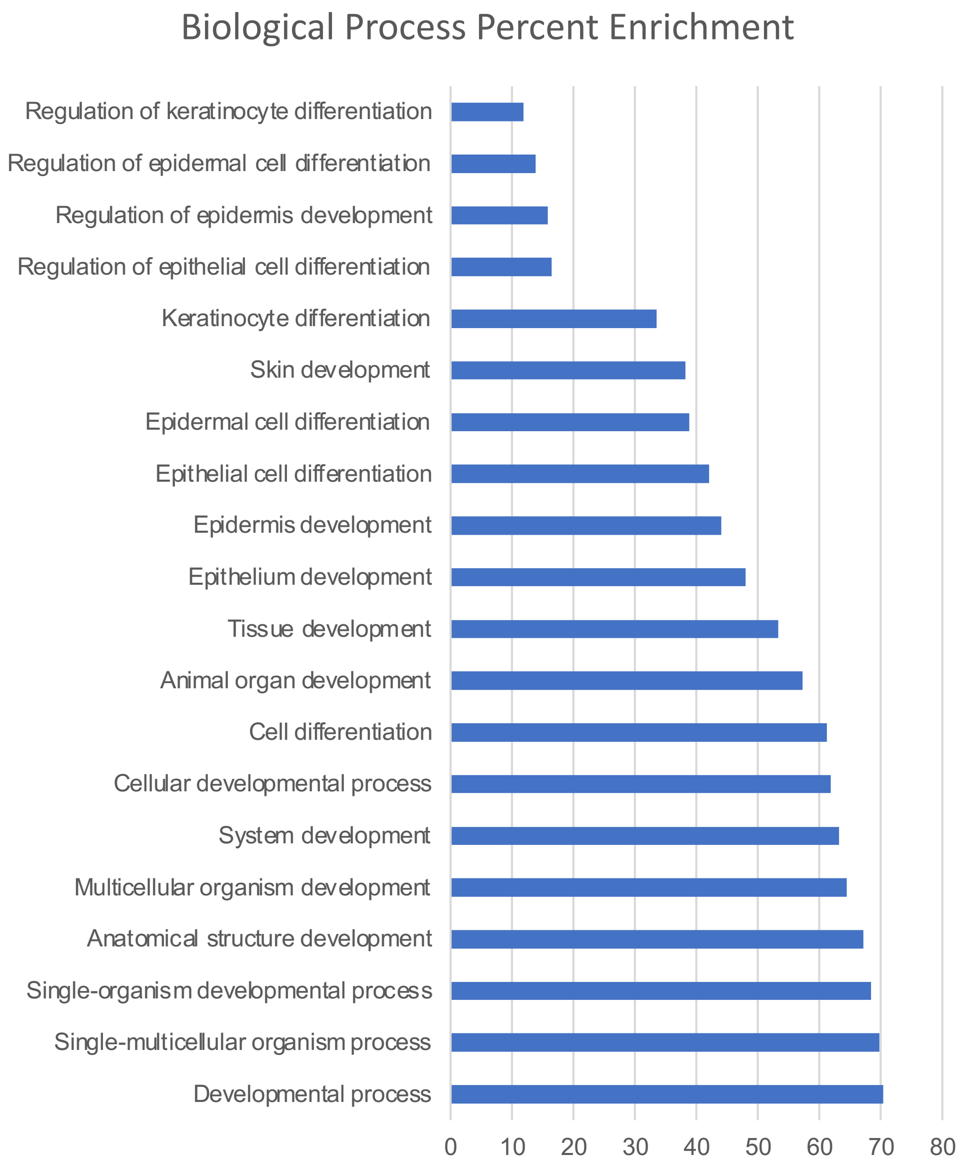

Biological Process Percent Enrichment
Regulation of keratinocyte differentiation
Regulation of epidermal cell differentiation
Regulation of epidermis development
Regulation of epithelial cell differentiation
Keratinocyte differentiation
Skin development
Epidermal cell differentiation
Epithelial cell differentiation
Epidermis development
Epithelium development
Tissue development
Animal organ development
Cell differentiation
Cellular developmental process
System development
Multicellular organism development
Anatomical structure development
Single-organism developmental process
Single-multicellular organism process
Developmental process
0
10
20
30
40
50
60
70
80

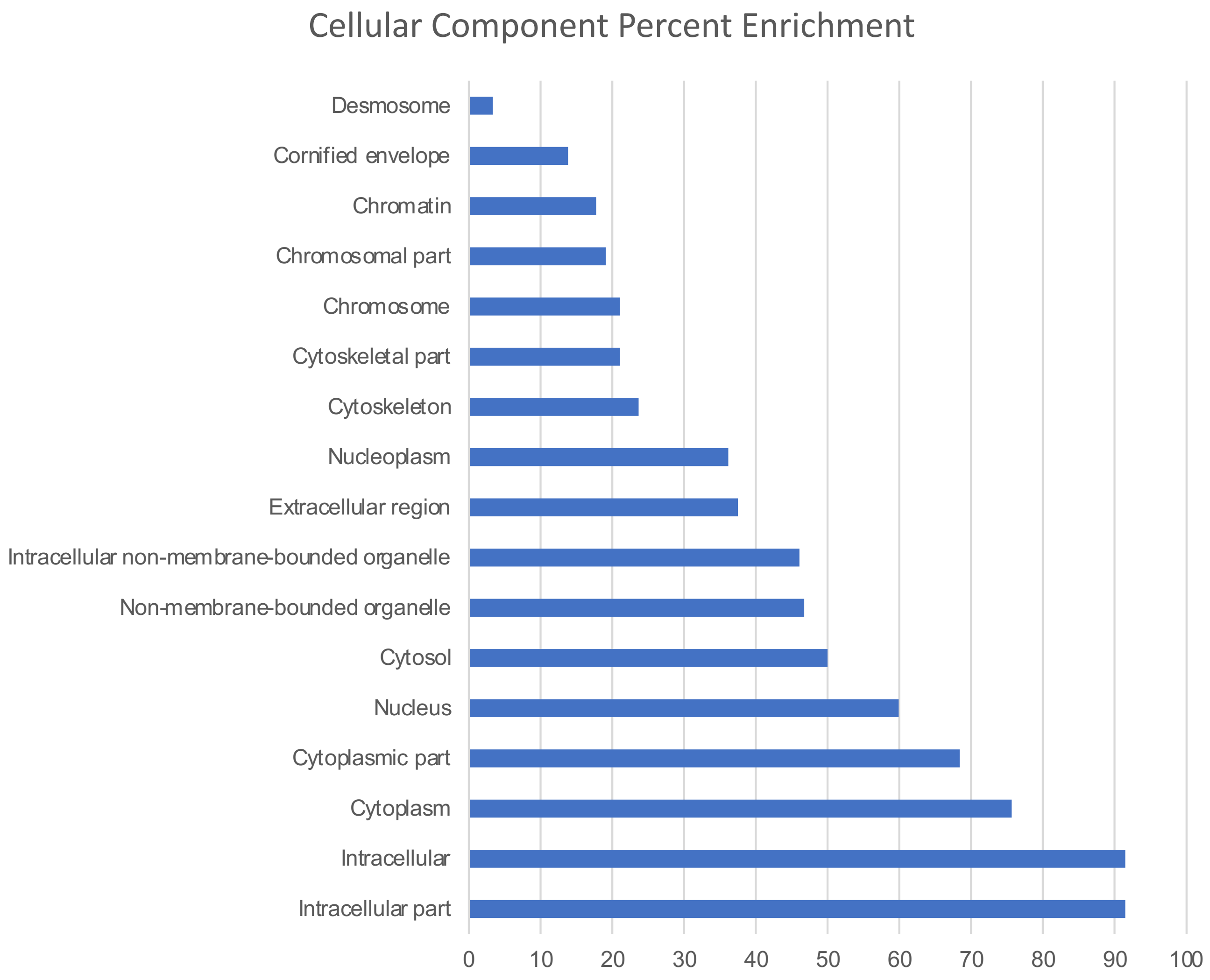
Cellular Component Percent Enrichment
Desmosome
Cornified envelope
Chromatin
Chromosomal part
Chromosome
Cytoskeletal part
Cytoskeleton
Nucleoplasm
Extracellular region
Intracellular non-membrane-bounded organelle
Non-membrane-bounded organelle
Cytosol
Nucleus
Cytoplasmic part
Cytoplasm
Intracellular
Intracellular part
0
10
20
30
40
50
60
70
80
90
100

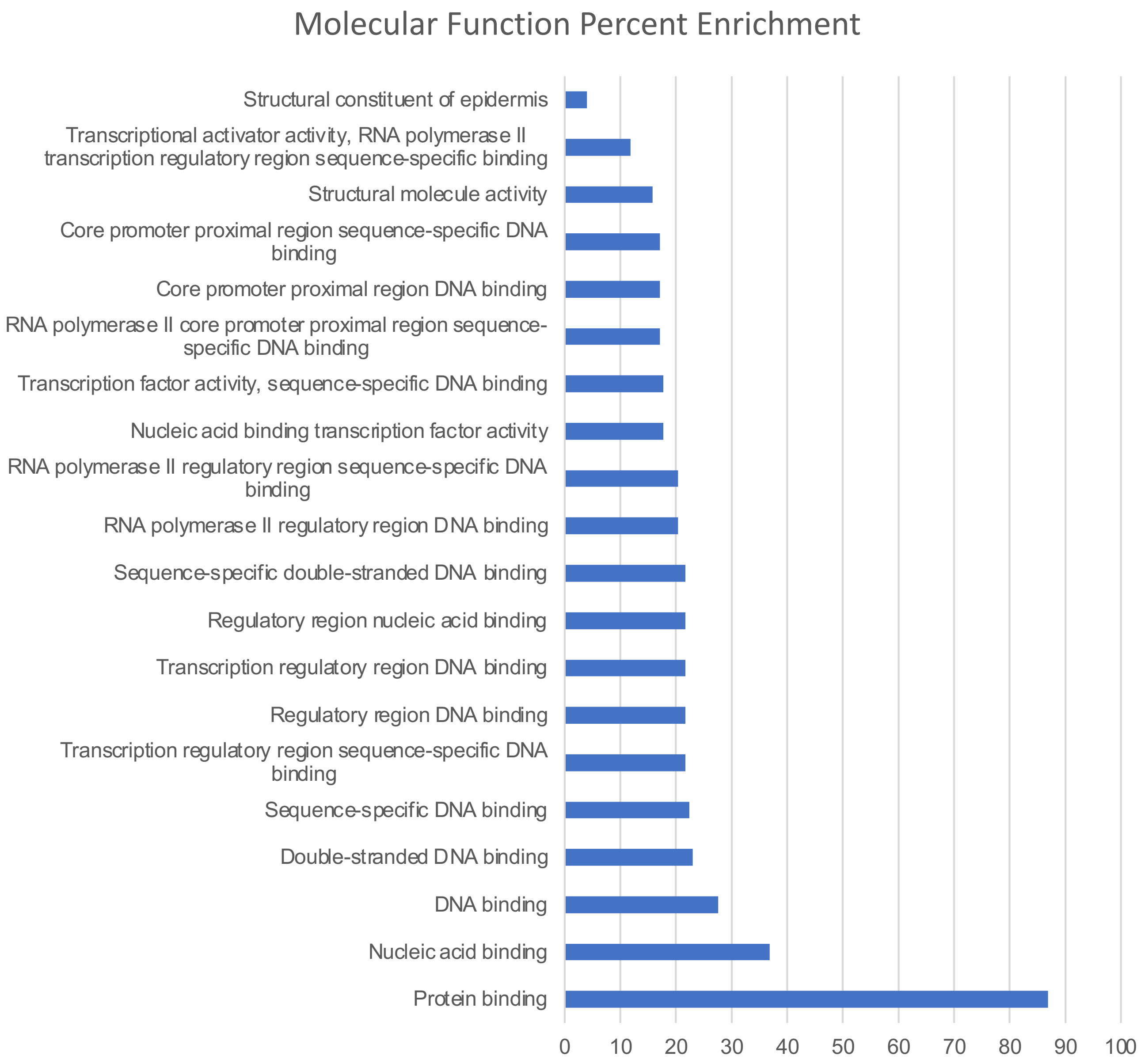
Molecular Function Percent Enrichment
Structural constituent of epidermis
Transcriptional activator activity, RNA polymerase II transcription regulatory region sequence-specific binding
Structural molecule activity
Core promoter proximal region sequence-specific DNA binding
Core promoter proximal region DNA binding
RNA polymerase II core promoter proximal region sequence-specific DNA binding
Transcription factor activity, sequence-specific DNA binding
Nucleic acid binding transcription factor activity
RNA polymerase II regulatory region sequence-specific DNA binding
RNA polymerase II regulatory region DNA binding
Sequence-specific double-stranded DNA binding
Regulatory region nucleic acid binding
Transcription regulatory region DNA binding
Regulatory region DNA binding
Transcription regulatory region sequence-specific DNA binding
Sequence-specific DNA binding
Double-stranded DNA binding
DNA binding
Nucleic acid binding
Protein binding
0
10
20
30
40
50
60
70
80
90
100
