# Supplementary material for: Thermostable Proteins from HaCaT Keratinocytes Identify a Wide Breadth of Intrinsically Disordered Proteins and Candidates for Liquid–Liquid Phase Separation
Source: Int J Mol Sci. 2022 Nov 18;23(22):14323. doi: 10.3390/ijms232214323 (PMC9692912; doi:10.3390/ijms232214323)
Supplement: Supplementary file 1 [file ijms-23-14323-s001.zip › Supplementary Table S1 NOV08.pdf]

Supplementary Table S1. Proteins identified by LC-MS/MS from heat lysate of HaCaT keratinocytes.

Accession	Protein Name	RAPID % Disorder	SLIDER IDR propensity score (0-1)	Total Spectrum Count Sample 1	Average Precursor Intensity Sample 1	Total Spectrum Count Sample 2	Average Precursor Intensity Sample 2
Q9UII2	ATPase inhibitor, mitochondrial	100	0.727	3	4.67E+07	17	7.49E+07
O00479	High mobility group nucleosome-binding domain-containing protein 4	100	0.947	2	4.96E+07	3	6.58E+07
P26583	High mobility group protein B2	100	0.957	50	1.02E+08	15	4.60E+07
P17096	High mobility group protein HMG-I/HMG-Y	100	0.932	6	8.77E+07	3	2.20E+07
F5H2A4	High mobility group protein HMGI-C	100	0.914	2	1	2	1.40E+07
P16403	Histone H1.2	100	0.810	14	8.89E+07	12	7.36E+07
P10412	Histone H1.4	100	0.796	13	9.55E+07	13	6.58E+07
P16401	Histone H1.5	100	0.760	8	3.12E+07	13	2.81E+07
Q9GZP8	Immortalization up-regulated protein	100	0.876	2	6.90E+07	3	1.46E+07
P05204	Non-histone chromosomal protein HMG-17	100	0.957	5	7.30E+07	2	4.12E+08
Q9H1E3	Nuclear ubiquitous casein and cyclin-dependent kinase substrate 1	100	0.974	11	1.24E+08	13	1.02E+08
P20962	Parathymosin	100	0.987	4	9.59E+07	5	6.52E+07
B8ZZQ6	Prothymosin alpha	100	0.972	12	7.06E+07	6	1.69E+08
P16949	Stathmin	100	0.867	14	6.44E+07	13	3.52E+07
P63313	Thymosin beta-10	100	0.767	8	1.57E+08	3	1.23E+07
P62328	Thymosin beta-4	100	0.846	26	1.53E+08	6	4.18E+07
Q5H9L2	Transcription elongation factor A protein-like 5	100	0.936	4	1.56E+07	1	4.27E+07
Q9Y2S6	Translation machinery-associated protein 7	100	0.915	2	1.19E+07	1	2.21E+07
Q9UK76	Jupiter microtubule associated homolog 1	97.4	0.776	9	3.89E+07	14	3.28E+07
P29966	Myristoylated alanine-rich C-kinase substrate	97.29	0.961	6	2.95E+07	4	2.63E+07
P61244	Protein max	94.38	0.858	4	2.56E+07	1	1.28E+07
Q8IV56	Proline-rich protein 15	93.8	0.870	2	2.26E+07	1	1.65E+07

Accession	Protein Name	RAPID % Disorder	SLIDER IDR propensity score (0-1)	Total Spectrum Count Sample 1	Average Precursor Intensity Sample 1	Total Spectrum Count Sample 2	Average Precursor Intensity Sample 2
Q13442	28 kDa heat- and acid-stable phosphoprotein	93.37	0.919	10	3.91E+07	7	2.07E+07
Q14978	Nucleolar and coiled-body phosphoprotein 1	86.98	0.952	14	2.13E+07	9	1.58E+07
P47914	60S ribosomal protein L29	86.16	0.878	2	5.64E+07	3	1.66E+07
F8W7E0	Calpain inhibitor (Fragment)	84.48	0.939	27	9.90E+07	29	3.71E+07
Q8WW12	PEST proteolytic signal-containing nuclear protein	84.27	0.873	7	6.20E+07	15	3.37E+07
H3BMD8	cAMP-regulated phosphoprotein 19	83.97	0.843	4	4.04E+07	2	1.98E+07
Q96CT7	Coiled-coil domain-containing protein 124	81.17	0.909	6	1.17E+07	5	3.75E+07
H3BMM9	RNA-binding protein with serine-rich domain 1 (Fragment)	80.63	0.951	8	4.30E+07	1	6142400
P09429	High mobility group protein B1	80.47	0.965	87	2.23E+08	32	9.24E+07
Q8N6N3	UPF0690 protein C1orf52	80.22	0.893	1	1.57E+07	2	7808200
P35321	Cornifin-A	79.78	0.849	10	7.07E+07	2	1
P51858	Hepatoma-derived growth factor	79.17	0.903	46	1.91E+08	15	2.83E+07
O60869	Endothelial differentiation-related factor 1	79.05	0.619	6	3.58E+07	3	4.90E+07
P20810	Calpastatin	78.39	0.957	74	7.10E+07	21	3.82E+07
A0A0A0MRI1	GPALPP motifs-containing protein 1	77.51	0.942	5	2.38E+07	4	1.05E+07
A0A0C4DGB5	Calpain inhibitor	77.46	0.951	75	6.76E+07	31	3.38E+07
J3KR35	Coiled-coil domain containing 12, isoform CRA_a	76.54	0.878	5	1.80E+07	6	3.25E+07
Q9H444	Charged multivesicular body protein 4b	76.34	0.871	13	3.89E+07	12	2.14E+07
Q9Y3B9	RRP15-like protein	75.89	0.909	4	2.79E+07	2	7102000
Q13428	Treacle protein	75.67	0.979	17	1.61E+07	8	1.20E+07
Q9BQ61	Telomerase RNA component interacting Rnase	75.57	0.843	5	2.63E+07	6	1.57E+07
Q9BRP8	Partner of Y14 and mago	75.49	0.902	16	2.48E+07	11	1.24E+07
P53999	Activated RNA polymerase II transcriptional coactivator p15	74.8	0.816	12	2.34E+07	4	1.91E+07
E9PR30	40S ribosomal protein S30	74.49	0.540	3	5.20E+07	1	6.46E+07

Accession	Protein Name	RAPID % Disorder	SLIDER IDR propensity score (0-1)	Total Spectrum Count Sample 1	Average Precursor Intensity Sample 1	Total Spectrum Count Sample 2	Average Precursor Intensity Sample 2
Q9NX55	Huntingtin-interacting protein K	74.38	0.836	3	8392100	1	3119100
Q96NB3	Zinc finger protein 830	74.19	0.898	4	8953200	2	6620700
P22528	Cornifin-B	74.16	0.816	11	5.99E+07	2	1.02E+07
Q9NWB6	Arginine and glutamate-rich protein 1	73.63	0.980	3	3.41E+07	1	2.84E+07
Q07283	Trichohyalin	73.6	0.960	1	1	2	1
Q9UEE9	Craniofacial development protein 1	73.24	0.939	10	2.83E+07	6	2.59E+07
P41236	Protein phosphatase inhibitor 2	72.2	0.926	6	2.43E+07	2	1.33E+07
A6NMQ3	Alpha-endosulfine	72.14	0.869	3	3.79E+07	1	8910000
Q9H910	Jupiter microtubule associated homolog 2	72.11	0.768	10	4.66E+07	7	2.09E+07
Q9UKY7	Protein CDV3 homolog	71.71	0.917	11	7.21E+07	10	2.01E+07
Q9Y2W2	WW domain-binding protein 11	71.29	0.962	7	2.10E+07	1	4965300
Q9BW04	Specifically androgen-regulated gene protein	71.21	0.929	34	7.72E+07	19	3.05E+07
E9PP13	DNA-directed RNA polymerase II subunit GRINL1A, isoforms 4/5	71.05	0.606	2	3.78E+07	4	1.72E+07
Q9H0G5	Nuclear speckle splicing regulatory protein 1	70.97	0.956	4	9385800	1	1.09E+07
P62316	Small nuclear ribonucleoprotein Sm D2	70.34	0.650	4	1.72E+07	3	6645400
P07919	Cytochrome b-c1 complex subunit 6, mitochondrial	69.23	0.778	4	2.46E+08	11	9.61E+07
Q8TF74	WAS/WASL-interacting protein family member 2	69.09	0.912	9	3.01E+07	1	1
O60927	E3 ubiquitin-protein ligase PPP1R11	69.05	0.826	2	1	2	1.20E+07
O75475	PC4 and SFRS1-interacting protein	68.3	0.956	9	1.23E+08	3	1.73E+07
O00515	Ladinin-1	68.28	0.931	39	6.39E+07	26	2.82E+07
Q96IZ0	PRKC apoptosis WT1 regulator protein	68.24	0.926	6	2.91E+07	6	1.43E+07
Q9BXV9	EKC/KEOPS complex subunit GON7	68	0.688	3	1.14E+07	1	5668800
Q7Z4V5	Hepatoma-derived growth factor-related protein 2	67.81	0.968	16	7.13E+07	6	1.51E+07
I3L1Y9	FLYWCH family member 2	67.36	0.841	4	2.00E+07	1	6674800

Accession	Protein Name	RAPID % Disorder	SLIDER IDR propensity score (0-1)	Total Spectrum Count Sample 1	Average Precursor Intensity Sample 1	Total Spectrum Count Sample 2	Average Precursor Intensity Sample 2
P82979	SAP domain-containing ribonucleoprotein	67.14	0.871	13	7.57E+07	8	1.26E+08
O95218	Zinc finger Ran-binding domain-containing protein 2	66.97	0.937	6	6.29E+07	8	3.79E+07
Q9P013	Spliceosome-associated protein CWC15 homolog	66.81	0.925	5	2.15E+07	7	4.59E+07
A0A0D9SEM4	Serine/arginine-rich-splicing factor 4 (Fragment)	66.67	0.888	14	4.71E+07	5	1.70E+07
A0A0G2JHC2	PPP1R18	66.23	0.927	4	9960800	1	1
P07476	Involucrin	65.81	0.917	198	2.08E+08	19	3.41E+07
Q9Y2W1	Thyroid hormone receptor-associated protein 3	65.65	0.963	10	1.85E+07	5	1.63E+07
Q9NRF9	DNA polymerase epsilon subunit 3	65.31	0.905	7	1.23E+07	2	1.18E+07
Q9BRD0	BUD13 homolog	64.94	0.944	2	1.12E+07	2	1
P35269	General transcription factor IIF subunit 1	64.6	0.953	17	2.82E+07	11	1.24E+07
J3KT73	60S ribosomal protein L38	64.06	0.722	2	2.46E+07	2	1
C9JSZ1	Far upstream element-binding protein 1 (Fragment)	63.79	0.656	18	6.72E+07	10	1.96E+07
E7ERS3	Zinc finger CCCH domain-containing protein 18	63.77	0.981	5	1.63E+07	1	1.88E+07
E9PL71	Elongation factor 1-delta (Fragment)	63.64	0.853	27	6.05E+07	7	2.92E+07
Q8IYL3	UPF0688 protein C1orf174	63.37	0.889	2	1.79E+07	1	2.31E+07
F5H7W8	Protein CUSTOS	62.8	0.856	4	8238500	1	8042000
Q96B36	Proline-rich AKT1 substrate 1	62.5	0.875	4	1.07E+07	3	4233900
P38159	RNA-binding motif protein, X chromosome	62.4	0.878	6	1.22E+07	1	1.15E+07
P19338	Nucleolin	62.39	0.938	66	5.66E+07	19	2.98E+07
Q9UQ35	Serine/arginine repetitive matrix protein 2	62.28	0.990	9	2.16E+07	3	1.88E+07
P82909	28S ribosomal protein S36, mitochondrial	62.14	0.641	3	1.55E+07	5	1.29E+08
J3KRP6	Protein SSXT	62.07	0.460	2	1.24E+07	2	1.30E+07
O60814	Histone H2B type 1-K	61.9	0.641	11	1.63E+08	25	3.65E+07
P67809	Y-box-binding protein 1	61.42	0.883	23	6.94E+07	2	1.35E+07

Accession	Protein Name	RAPID % Disorder	SLIDER IDR propensity score (0-1)	Total Spectrum Count Sample 1	Average Precursor Intensity Sample 1	Total Spectrum Count Sample 2	Average Precursor Intensity Sample 2
Q13247	Serine/arginine-rich splicing factor 6	61.34	0.880	15	1.00E+08	6	4.25E+07
P23527	Histone H2B type 1-O	61.11	0.642	9	1.12E+08	27	3.51E+07
H0YJV7	Transcriptional repressor protein YY1 (Fragment)	61.05	0.703	2	2.37E+07	1	6344500
O75822	Eukaryotic translation initiation factor 3 subunit J	60.85	0.822	20	2.54E+07	1	8793700
P45973	Chromobox protein homolog 5	60.73	0.846	11	4.76E+07	2	2.07E+07
Q8NC51	Plasminogen activator inhibitor 1 RNA-binding protein	60.54	0.905	29	4.47E+07	20	3.71E+07
Q92733	Proline-rich protein PRCC	60.49	0.909	5	3.04E+07	3	1.45E+07
Q04323	UBX domain-containing protein 1	60.27	0.881	13	1.72E+07	2	7808900
H3BMX9	PSME3-interacting protein (Fragment)	60	0.864	8	1.50E+07	1	1.81E+07
G8JLD3	ELKS/Rab6-interacting/CAST family member 1	59.76	0.936	7	1.08E+07	1	7384500
A0A1W2PQ43	Bcl-2-associated transcription factor 1	59.74	0.954	5	1.11E+07	1	1
Q01105	Protein SET	59.66	0.903	36	1.45E+08	12	2.67E+07
S4R3H4	Apoptotic chromatin condensation inducer in the nucleus	59.31	0.985	32	2.23E+07	14	1.27E+07
E7EVA0	Microtubule-associated protein	59.16	0.941	45	3.86E+07	7	3.03E+07
C9JFK9	BAG family molecular chaperone regulator 3 (Fragment)	58.77	0.894	17	5.55E+07	7	2.45E+07
Q13435	Splicing factor 3B subunit 2	57.99	0.958	16	1.91E+07	3	1.58E+07
F8W1I5	Myosin light chain 6B	57.71	0.690	7	2.94E+07	3	4.36E+07
O94992	Protein HEXIM1	57.66	0.928	18	4.21E+07	1	9198900
Q8WVC0	RNA polymerase-associated protein LEO1	57.51	0.962	7	1.97E+07	1	8745300
Q9UHB6	LIM domain and actin-binding protein 1	57.44	0.892	29	2.10E+07	1	1.62E+07
P37108	Signal recognition particle 14 kDa protein	57.35	0.787	9	5.34E+07	1	1.07E+07
P06753	Tropomyosin alpha-3 chain	57.19	0.894	66	3.49E+08	25	1.03E+08
Q15370	Elongin-B	56.78	0.541	8	1.78E+07	1	1.04E+07

Accession	Protein Name	RAPID % Disorder	SLIDER IDR propensity score (0-1)	Total Spectrum Count Sample 1	Average Precursor Intensity Sample 1	Total Spectrum Count Sample 2	Average Precursor Intensity Sample 2
P62979	Ubiquitin-40S ribosomal protein S27a	56.41	0.724	11	3.71E+07	1	1.25E+07
Q9BTT0	Acidic leucine-rich nuclear phosphoprotein 32 family member E	56.34	0.888	7	1.00E+08	2	1
O60828	Polyglutamine-binding protein 1	56.23	0.900	7	3.11E+07	3	3.26E+07
O75937	DnaJ homolog subfamily C member 8	56.13	0.882	12	4.17E+07	1	8259300
O75494	Serine/arginine-rich splicing factor 10	56.11	0.898	17	3.39E+07	6	1.04E+07
O75391	Sperm-associated antigen 7	55.95	0.837	4	1.74E+07	1	1859000
P41208	Centrin-2	55.81	0.829	7	1.91E+07	2	1.57E+07
Q13185	Chromobox protein homolog 3	55.74	0.779	18	7.07E+07	4	2.37E+07
P05386	60S acidic ribosomal protein P1	55.26	0.442	10	1.01E+08	3	4.96E+07
Q9P2E9	Ribosome-binding protein 1	55.25	0.956	13	1.36E+07	1	1
H7BYY1	Tropomyosin 1 (Alpha), isoform CRA_m	55.24	0.883	41	1.30E+08	17	6.13E+07
P43487	Ran-specific GTPase-activating protein	55.22	0.856	19	1.46E+08	7	4.17E+07
P55081	Microfibrillar-associated protein 1	54.9	0.929	5	2.77E+07	5	1.47E+07
E7EPN9	Protein PRRC2C	54.84	0.974	8	8488100	4	7531300
P48634	Protein PRRC2A	54.47	0.962	3	9937600	1	1
Q32MZ4	Leucine-rich repeat flightless-interacting protein 1	54.33	0.949	28	2.83E+07	6	1.66E+07
O75348	V-type proton ATPase subunit G 1	54.24	0.831	11	2.93E+07	7	1.68E+07
H3BPE7	RNA-binding protein FUS	53.89	0.800	8	3.31E+07	1	1.98E+07
O76070	Gamma-synuclein	53.54	0.663	16	1.09E+08	8	3.09E+07
P06748	Nucleophosmin	53.4	0.936	58	1.49E+08	29	3.59E+07
F2Z2K0	NSFL1 cofactor p47	53.28	0.764	20	1.29E+08	4	2.41E+07
O15234	Protein CASC3	53.2	0.932	5	3.17E+07	2	6590600
P05387	60S acidic ribosomal protein P2	53.04	0.638	26	1.28E+08	5	6991500
B5MBW9	Coiled-coil-helix-coiled-coil-helix domain-containing protein 10, mitochondrial	53.02	0.675	1	2.47E+07	3	8618200

Accession	Protein Name	RAPID % Disorder	SLIDER IDR propensity score (0-1)	Total Spectrum Count Sample 1	Average Precursor Intensity Sample 1	Total Spectrum Count Sample 2	Average Precursor Intensity Sample 2
P09497	Clathrin light chain B	52.84	0.834	11	8.31E+07	8	3.43E+07
P67936	Tropomyosin alpha-4 chain	52.82	0.891	83	1.58E+08	27	7.01E+07
Q96CF2	Charged multivesicular body protein 4c	52.79	0.869	2	1.06E+08	2	3.33E+07
P16989	Y-box-binding protein 3	52.69	0.847	19	6.47E+07	1	1
E7EX17	Eukaryotic translation initiation factor 4B	52.44	0.957	19	5.64E+07	18	1.95E+07
J3QQJ0	SAP30-binding protein (Fragment)	52.31	0.907	6	1.73E+07	1	1.39E+07
H7C2K6	Band 4.1-like protein 1 (Fragment)	52.24	0.873	4	1.27E+07	2	1
E7EMZ9	Transforming acidic coiled-coil-containing protein 2	52.02	0.953	49	2.23E+07	5	1.68E+07
Q8IVM0	Coiled-coil domain-containing protein 50	51.96	0.910	3	1.32E+07	3	1.13E+07
P20290	Transcription factor BTF3	51.94	0.824	19	3.55E+07	12	1.74E+07
Q96K17	Transcription factor BTF3 homolog 4	51.9	0.761	3	2.63E+07	7	2.55E+07
O95817	BAG family molecular chaperone regulator 3	51.83	0.921	22	4.83E+07	7	2.45E+07
P46937	Transcriptional coactivator YAP1	51.59	0.892	27	3.85E+07	8	2.77E+07
I3L2R9	Nuclear distribution protein nudE homolog 1 (Fragment)	51.45	0.819	1	3504900	2	1133200
Q9C0C2	182 kDa tankyrase-1-binding protein	51.13	0.928	78	3.90E+07	43	2.53E+07
Q9H3Q1	Cdc42 effector protein 4	51.12	0.847	5	9762400	1	8939000
Q6ZUT6	Coiled-coil domain-containing protein 9B	50.94	0.905	5	1.66E+07	2	1.04E+07
K7EL20	Eukaryotic translation initiation factor 3 subunit G (Fragment)	50.76	0.841	9	5.64E+07	2	2.33E+07
Q92945	Far upstream element-binding protein 2	50.63	0.768	71	9.71E+07	25	3.83E+07
Q15637	Splicing factor 1	50.39	0.890	24	2.47E+07	8	1.17E+07
J3QTA6	MICOS complex subunit MIC25	50.39	0.787	1	2.44E+07	2	1.25E+07
Q15847	Adipogenesis regulatory factor	50	0.668	22	1.20E+08	30	8.92E+07
C9J0D1	Histone H2A	50	0.401	2	5.31E+07	7	3.51E+07

Accession	Protein Name	RAPID % Disorder	SLIDER IDR propensity score (0-1)	Total Spectrum Count Sample 1	Average Precursor Intensity Sample 1	Total Spectrum Count Sample 2	Average Precursor Intensity Sample 2
Q8IVF2	Protein AHNAK2	49.75	0.906	61	1.73E+07	1	1
Q13526	Peptidyl-prolyl cis-trans isomerase NIMA-interacting 1	49.69	0.711	7	3.82E+07	2	1
E9PM92	Small acidic protein	49.68	0.646	7	4.71E+07	4	2.43E+07
Q13573	SNW domain-containing protein 1	49.63	0.901	14	1.03E+07	6	1.83E+07
O15212	Prefoldin subunit 6	49.61	0.715	17	4.34E+07	10	2.41E+07
P50552	Vasodilator-stimulated phosphoprotein	49.47	0.927	7	1.88E+07	2	1
Q9UHV9	Prefoldin subunit 2	49.35	0.681	20	6.16E+07	3	2.49E+07
C9JSU1	Leucine-rich repeat flightless-interacting protein 2 (Fragment)	49.33	0.928	3	2.11E+07	1	1
P98179	RNA-binding protein 3	49.04	0.472	8	1.77E+07	4	2.01E+07
P18615	Negative elongation factor E	48.95	0.928	11	2.18E+07	4	2.16E+07
P23246	Splicing factor, proline- and glutamine-rich	48.94	0.917	56	4.52E+07	16	4.20E+07
P49321	Nuclear autoantigenic sperm protein	48.86	0.926	28	3.79E+07	1	2.56E+07
E5RGX5	Stathmin	48.81	0.789	5	1.09E+08	3	3.23E+07
M0QY97	Zinc finger CCCH domain-containing protein 4 (Fragment)	48.79	0.941	6	8856300	2	3821500
Q9ULR0	Pre-mRNA-splicing factor ISY1 homolog	48.77	0.857	7	1.97E+07	1	1.42E+07
P08621	U1 small nuclear ribonucleoprotein 70 kDa	48.51	0.940	3	6789400	1	1.32E+07
Q96AE4	Far upstream element-binding protein 1	48.45	0.775	43	6.86E+07	25	2.13E+07
Q09666	Neuroblast differentiation-associated protein AHNAK	48.37	0.851	647	8.06E+07	228	3.52E+07
Q9Y5S9	RNA-binding protein 8A	48.28	0.672	12	2.33E+07	2	1.03E+07
Q14011	Cold-inducible RNA-binding protein	48.26	0.597	12	2.38E+07	7	8570100
O75347	Tubulin-specific chaperone A	48.15	0.815	16	9.68E+07	7	5.31E+07
Q86U42	Polyadenylate-binding protein 2	48.04	0.887	9	4.77E+07	2	1.59E+07

Accession	Protein Name	RAPID % Disorder	SLIDER IDR propensity score (0-1)	Total Spectrum Count Sample 1	Average Precursor Intensity Sample 1	Total Spectrum Count Sample 2	Average Precursor Intensity Sample 2
Q9BW30	Tubulin polymerization-promoting protein family member 3	47.73	0.540	8	3.34E+07	8	1.96E+07
F8VVL1	Density-regulated protein	47.5	0.768	9	5.07E+07	1	1
Q14151	Scaffold attachment factor B2	47.43	0.951	13	2.88E+07	8	2.76E+07
Q9Y5U2	Protein TSSC4	47.11	0.750	5	7.13E+07	5	1.69E+07
O75940	Survival of motor neuron-related-splicing factor 30	46.64	0.835	2	4.43E+07	1	1
P55327	Tumor protein D52	46.43	0.761	14	6.29E+07	8	3.13E+07
Q6NZI2	Caveolae-associated protein 1	46.41	0.900	9	3.20E+07	5	2.99E+07
A0A087WYT3	Prostaglandin E synthase 3	46.34	0.669	19	4.87E+07	3	3.23E+07
Q13243	Serine/arginine-rich splicing factor 5	46.32	0.836	12	6.91E+07	6	2.87E+07
Q8WU90	Zinc finger CCCH domain-containing protein 15	46.24	0.858	5	1.77E+07	1	621120
Q6UN15	Pre-mRNA 3'-end-processing factor FIP1	46.13	0.901	6	1.50E+07	4	6018900
K7ENT6	Tropomyosin alpha-4 chain	46.12	0.874	50	1.87E+08	16	8.40E+07
Q15424	Scaffold attachment factor B1	46.12	0.961	14	3.96E+07	23	2.68E+07
O43399	Tumor protein D54	46.12	0.780	22	4.78E+07	9	1.88E+07
P17535	Transcription factor jun-D	46.11	0.872	3	2.34E+07	1	7690900
O95926	Pre-mRNA-splicing factor SYF2	46.09	0.834	3	1.64E+07	1	3746600
D6R9P3	Heterogeneous nuclear ribonucleoprotein A/B	46.07	0.817	24	6.21E+07	10	2.04E+07
Q07666	KH domain-containing, RNA-binding, signal transduction-associated protein 1	46.05	0.856	7	3.34E+07	2	3.82E+07
B2R5W2	Heterogeneous nuclear ribonucleoproteins C1/C2	45.86	0.846	13	4.45E+07	4	2.35E+07
Q86WV7	Coiled-coil domain-containing protein 43	45.81	0.844	3	1.94E+07	1	7113900
Q9Y2V2	Calcium-regulated heat-stable protein 1	45.58	0.503	9	5.83E+07	7	2.17E+07
Q14157	Ubiquitin-associated protein 2-like	45.17	0.888	25	1.85E+07	4	6284300
Q8WXX5	DnaJ homolog subfamily C member 9	45	0.728	11	1.69E+07	3	6191700
E9PK01	Elongation factor 1-delta (Fragment)	44.83	0.807	35	7.87E+07	11	3.98E+07

Accession	Protein Name	RAPID % Disorder	SLIDER IDR propensity score (0-1)	Total Spectrum Count Sample 1	Average Precursor Intensity Sample 1	Total Spectrum Count Sample 2	Average Precursor Intensity Sample 2
A0A087WZX0	B-cell CLL/lymphoma 9-like protein	44.53	0.953	3	1.87E+07	3	1.22E+07
Q8WWM7	Ataxin-2-like protein	44.47	0.896	9	1.85E+07	2	8267900
Q5HYB6	Q5HYB6	44.4	0.876	106	3.71E+08	44	7.03E+07
Q15075	Early endosome antigen 1	44.29	0.898	16	1.34E+07	1	1
P61956	Small ubiquitin-related modifier 2	44.21	0.549	5	3.05E+07	3	2.02E+07
P84103	Serine/arginine-rich splicing factor 3	43.9	0.767	17	1.30E+08	15	7.05E+07
Q16204	Coiled-coil domain-containing protein 6	43.67	0.946	30	3.20E+07	3	1.00E+07
Q9C005	Protein dpy-30 homolog	43.43	0.529	9	5.48E+07	4	1.26E+07
I3L1Q2	B-cell CLL/lymphoma 7 protein family member C (Fragment)	43.34	0.857	2	2.33E+07	1	1
F8W6I7	Helix-destabilizing protein	43.32	0.645	46	7.77E+07	15	4.08E+07
O14745	Na(+)/H(+) exchange regulatory cofactor NHE-RF1	43.3	0.844	35	9.87E+07	14	2.36E+07
O14737	Programmed cell death protein 5	43.2	0.778	6	4.08E+07	1	6.25E+07
O43290	U4/U6.U5 tri-snRNP-associated protein 1	43.12	0.934	10	9976100	2	8675000
Q99614	Tetratricopeptide repeat protein 1	42.81	0.810	11	2.48E+07	2	4.67E+07
Q86V81	THO complex subunit 4	42.8	0.762	15	3.47E+07	7	3.33E+07
C9JFR7	Cytochrome c (Fragment)	42.57	0.341	11	3.31E+07	1	1.46E+07
Q13595	Transformer-2 protein homolog alpha	42.55	0.820	13	3.95E+07	4	7453500
Q96C19	EF-hand domain-containing protein D2	42.5	0.825	21	4.67E+07	4	1.04E+07
O15541	E3 ubiquitin-protein ligase RNF113A	42.27	0.755	4	2.24E+07	1	9531900
A0A087WUK2	Heterogeneous nuclear ribonucleoprotein D-like	42.15	0.838	25	5.12E+07	15	3.63E+07
J3KP15	Serine/arginine-rich splicing factor 2 (Fragment)	42.11	0.691	19	6.58E+07	12	5.83E+07
Q9P2M7	Cingulin	42.06	0.927	6	1.18E+07	4	5799300
O14974	Protein phosphatase 1 regulatory subunit 12A	42.04	0.946	17	1.36E+07	3	9335400
P56385	ATP synthase subunit e, mitochondrial	42.03	0.483	1	6221400	5	3.75E+07

Accession	Protein Name	RAPID % Disorder	SLIDER IDR propensity score (0-1)	Total Spectrum Count Sample 1	Average Precursor Intensity Sample 1	Total Spectrum Count Sample 2	Average Precursor Intensity Sample 2
Q8N129	Protein canopy homolog 4	41.94	0.765	1	1.79E+07	2	4539200
Q06323	Proteasome activator complex subunit 1	41.77	0.714	19	7.76E+07	2	1205100
Q9HB71	Calcyclin-binding protein	41.67	0.639	18	2.71E+07	2	6117300
O95171	Sciellin	41.57	0.861	7	1.93E+07	1	5642300
Q15056	Eukaryotic translation initiation factor 4H	41.53	0.716	22	5.50E+07	13	4.12E+07
Q96JY6	PDZ and LIM domain protein 2	41.48	0.777	6	1.37E+07	2	1.12E+07
Q7Z5L9	Interferon regulatory factor 2-binding protein 2	41.4	0.847	8	1.84E+07	2	1.97E+07
P12270	Nucleoprotein TPR	41.18	0.952	37	1.51E+07	3	938120
P62263	40S ribosomal protein S14	41.06	0.546	4	3.48E+07	1	2.45E+07
Q15233	Non-POU domain-containing octamer-binding protein	40.98	0.857	16	3.49E+07	7	3.55E+07
C9JRZ6	MICOS complex subunit MIC19	40.95	0.843	2	2.32E+07	3	2.05E+07
B0QYK0	RNA-binding protein EWS	40.94	0.847	11	5.98E+07	5	4.86E+07
Q99584	Protein S100-A13	40.82	0.458	22	3.34E+07	2	1.82E+07
Q9UMD9	Collagen alpha-1(XVII) chain	40.75	0.913	1	7793200	2	8375700
P62995	Transformer-2 protein homolog beta	40.62	0.822	19	3.40E+07	3	6.24E+07
P50502	Hsc70-interacting protein	40.38	0.898	30	7.97E+07	8	1.07E+07
J3KRE2	Rho GDP-dissociation inhibitor 1	40.31	0.634	13	1.28E+08	4	2.13E+07
P50479	PDZ and LIM domain protein 4	40.3	0.704	11	3.09E+07	5	1.08E+07
P52566	Rho GDP-dissociation inhibitor 2	40.3	0.659	13	2.98E+07	1	1
Q14847	LIM and SH3 domain protein 1	40.23	0.717	47	1.13E+08	23	6.14E+07
Q15942	Zyxin	40.21	0.842	26	2.51E+07	6	1.39E+07
O95400	CD2 antigen cytoplasmic tail-binding protein 2	40.18	0.832	7	2.09E+07	1	1.03E+07
Q14247	Src substrate cortactin	39.82	0.801	72	7.40E+07	33	2.41E+07
O14602	Eukaryotic translation initiation factor 1A, Y-chromosomal	39.58	0.614	10	3.25E+07	1	1

Accession	Protein Name	RAPID % Disorder	SLIDER IDR propensity score (0-1)	Total Spectrum Count Sample 1	Average Precursor Intensity Sample 1	Total Spectrum Count Sample 2	Average Precursor Intensity Sample 2
Q99961	Endophilin-A2	39.13	0.798	12	2.30E+07	1	3.05E+07
K7ELL7	Glucosidase 2 subunit beta	39.07	0.939	30	7.69E+07	4	2.32E+07
P62805	Histone H4	38.83	0.320	2	9496300	2	2.10E+07
Q8WXF1	Paraspeckle component 1	38.81	0.890	10	1.36E+07	1	1684200
Q16777	Histone H2A type 2-C	38.76	0.538	10	8.47E+07	8	1.20E+08
P22626	Heterogeneous nuclear ribonucleoproteins A2/B1	38.53	0.608	71	8.89E+07	35	3.87E+07
F8WF69	Clathrin light chain	38.46	0.724	10	6.58E+07	2	6.16E+07
Q9BUH6	Protein PAXX	38.24	0.671	6	1.26E+07	1	1.69E+07
P62318	Small nuclear ribonucleoprotein Sm D3	38.1	0.548	4	1.32E+07	3	1.26E+07
O95721	Synaptosomal-associated protein 29	37.98	0.832	7	2.82E+07	1	8961300
P07108	Acyl-CoA-binding protein	37.93	0.418	5	3.53E+07	6	2.35E+07
Q12904	Aminoacyl tRNA synthase complex-interacting multifunctional protein 1	37.82	0.813	12	3.38E+07	1	7647000
Q13151	Heterogeneous nuclear ribonucleoprotein A0	37.7	0.566	4	2.66E+07	3	9220700
P04908	Histone H2A type 1-B/E	37.69	0.503	8	8.54E+07	10	3.39E+07
Q02818	Nucleobindin-1	37.53	0.865	21	1.75E+07	2	1.68E+07
E7EWR4	Cleavage stimulation factor subunit 2	37.19	0.875	21	2.36E+07	7	2.09E+07
P04792	Heat shock protein beta-1	37.07	0.641	82	2.44E+08	41	1.10E+08
P31948	Stress-induced-phosphoprotein 1	37.02	0.856	95	8.08E+07	13	1.87E+07
Q3BDU5	Prelamin-A/C	36.96	0.926	90	8.39E+07	33	3.47E+07
P51991	Heterogeneous nuclear ribonucleoprotein A3	36.51	0.674	27	5.99E+07	8	1.55E+07
P0DP23	Calmodulin-1	36.24	0.516	26	5.32E+07	5	5.45E+07
P60866	40S ribosomal protein S20	36.13	0.477	7	7.62E+07	2	1.66E+07
O60220	Mitochondrial import inner membrane translocase subunit Tim8 A	36.08	0.458	8	5.75E+07	4	1.86E+07

Accession	Protein Name	RAPID % Disorder	SLIDER IDR propensity score (0-1)	Total Spectrum Count Sample 1	Average Precursor Intensity Sample 1	Total Spectrum Count Sample 2	Average Precursor Intensity Sample 2
P62072	Mitochondrial import inner membrane translocase subunit Tim10	35.56	0.604	3	5.96E+07	2	1.92E+07
A0A1W2PPZ5	Transcription elongation factor A protein 1	35.55	0.837	14	3.79E+07	2	1.61E+07
O60925	Prefoldin subunit 1	35.25	0.669	11	3.58E+07	4	2.04E+07
Q13283	Ras GTPase-activating protein-binding protein 1	35.19	0.844	19	3.07E+07	2	1
P16220	Cyclic AMP-responsive element-binding protein 1	35.17	0.837	2	3.83E+07	1	2.60E+07
P27797	Calreticulin	35.01	0.863	30	4.77E+07	1	3383700
Q03252	Lamin-B2	34.84	0.907	6	6.70E+07	2	3.13E+07
Q9UMX0	Ubiquilin-1	34.8	0.911	15	6.15E+07	2	9.91E+07
P02545	Prelamin-A/C	34.79	0.902	97	8.62E+07	35	3.66E+07
Q9UJU6	Drebrin-like protein	34.42	0.852	44	5.55E+07	12	1.75E+07
P61981	14-3-3 protein gamma	34.41	0.650	15	7.95E+07	2	7.49E+07
Q14103	Heterogeneous nuclear ribonucleoprotein D0	34.37	0.831	42	8.21E+07	10	3.76E+07
E7EQR4	Ezrin	34.3	0.839	4	6922900	1	1
Q9UNF0	Protein kinase C and casein kinase substrate in neurons protein 2	34.16	0.874	11	3.03E+07	1	4151300
P35579	Myosin-9	34.08	0.914	19	1.17E+07	3	9364600
Q00688	Peptidyl-prolyl cis-trans isomerase FKBP3	33.93	0.584	29	6.03E+07	12	1.85E+07
O00151	PDZ and LIM domain protein 1	33.43	0.578	75	2.26E+08	30	5.87E+07
P10606	Cytochrome c oxidase subunit 5B, mitochondrial	33.33	0.599	12	9.78E+07	7	1.76E+08
A0A1W2PPS1	Heterogeneous nuclear ribonucleoprotein U	33.21	0.912	4	1.41E+07	2	5986600
Q9NRR5	Ubiquilin-4	32.95	0.900	9	2.72E+07	2	9.91E+07
P55036	26S proteasome non-ATPase regulatory subunit 4	32.89	0.823	22	3.43E+07	1	1
J3KTL2	Serine/arginine-rich splicing factor 1	32.81	0.760	47	1.06E+08	21	4.01E+07
J3QRS3	Myosin regulatory light chain 12A	32.77	0.589	10	1.14E+08	1	5.49E+07
Q96I24	Far upstream element-binding protein 3	32.69	0.674	28	3.23E+07	3	3.29E+07

Accession	Protein Name	RAPID % Disorder	SLIDER IDR propensity score (0-1)	Total Spectrum Count Sample 1	Average Precursor Intensity Sample 1	Total Spectrum Count Sample 2	Average Precursor Intensity Sample 2
P18859	ATP synthase-coupling factor 6, mitochondrial	32.41	0.506	8	9.36E+07	7	5.69E+07
P25685	DnaJ homolog subfamily B member 1	32.35	0.691	8	1.96E+07	1	1
P31947	14-3-3 protein sigma	32.26	0.754	74	1.87E+08	22	4.08E+07
Q9HCY8	Protein S100-A14	31.73	0.398	9	3.08E+07	2	3545800
P39019	40S ribosomal protein S19	31.72	0.500	6	4.24E+07	10	4.89E+07
P24534	Elongation factor 1-beta	31.56	0.728	21	9.55E+07	8	1.06E+08
P35268	60S ribosomal protein L22	31.25	0.525	4	4.98E+07	2	4758500
Q7Z7K0	COX assembly mitochondrial protein homolog	31.13	0.551	2	1.55E+07	3	1.57E+07
E7EQY1	Protein FAM136A	31.02	0.729	4	1.09E+07	4	1.24E+07
P11684	Uteroglobin	30.77	0.607	10	4.79E+07	7	2.11E+07
P14927	Cytochrome b-c1 complex subunit 7	30.63	0.456	11	6.14E+07	5	1.02E+08
P13645	Keratin, type I cytoskeletal 10	30.48	0.754	17	4.86E+07	7	2.19E+07
P00441	Superoxide dismutase [Cu-Zn]	29.87	0.272	21	1.50E+08	6	6.78E+07
O75607	Nucleoplasmin-3	29.78	0.696	5	2.47E+07	1	4628300
K7EKI8	Periplakin	29.7	0.904	84	2.52E+07	8	6638600
P04264	Keratin, type II cytoskeletal 1	29.5	0.813	8	1.20E+07	3	2.37E+07
P31431	Syndecan-4	29.29	0.645	3	2.57E+07	1	6405300
Q13404	Ubiquitin-conjugating enzyme E2 variant 1	29.25	0.636	5	2.58E+07	1	2.01E+07
P18669	Phosphoglycerate mutase 1	29.13	0.546	10	2.42E+07	1	2389900
O14545	TRAF-type zinc finger domain-containing protein 1	28.87	0.860	10	2.08E+07	1	1.50E+07
P0DN76	Splicing factor U2AF 35 kDa subunit-like protein	28.75	0.800	3	8680400	1	1862400
P63104	14-3-3 protein zeta/delta	28.57	0.712	53	6.43E+07	7	2.35E+07
O60664	Perilipin-3	28.57	0.731	45	4.06E+07	14	1.27E+07
B7Z4C8	60S ribosomal protein L31	28.46	0.554	3	4.32E+07	2	4.47E+07
D6RDG3	Transcription factor BTF3 (Fragment)	28.44	0.497	11	2.51E+07	8	1.30E+07

Accession	Protein Name	RAPID % Disorder	SLIDER IDR propensity score (0-1)	Total Spectrum Count Sample 1	Average Precursor Intensity Sample 1	Total Spectrum Count Sample 2	Average Precursor Intensity Sample 2
O43765	Small glutamine-rich tetratricopeptide repeat-containing protein alpha	27.8	0.769	9	7.30E+07	2	8709500
Q14512	Fibroblast growth factor-binding protein 1	27.78	0.674	6	3.96E+07	3	1.44E+07
Q9NPA8	Transcription and mRNA export factor ENY2	27.72	0.506	5	3.96E+07	5	1.86E+07
Q9H299	SH3 domain-binding glutamic acid-rich-like protein 3	26.88	0.384	5	3.03E+07	1	5.19E+07
O43852	Calumenin	26.67	0.517	10	3.30E+07	3	4481500
Q9H773	dCTP pyrophosphatase 1	26.47	0.640	7	1.76E+07	1	1
Q13242	Serine/arginine-rich splicing factor 9	26.24	0.527	16	2.75E+07	5	5.74E+07
O00625	Pirin	26.21	0.494	46	7.58E+07	3	1.97E+07
P62857	40S ribosomal protein S28	26.09	0.482	16	8.10E+07	12	3.28E+07
P08238	Heat shock protein HSP 90-beta	25.83	0.850	13	3.47E+07	2	3955100
O75947	ATP synthase subunit d, mitochondrial	25.47	0.496	14	7.23E+07	9	2.30E+07
H0YN26	Acidic leucine-rich nuclear phosphoprotein 32 family member A	25.42	0.664	29	1.77E+08	14	3.50E+07
Q5T6W2	Heterogeneous nuclear ribonucleoprotein K (Fragment)	25.33	0.802	52	1.22E+08	10	1.54E+07
P00568	Adenylate kinase isoenzyme 1	25.26	0.479	28	7.78E+07	4	1.40E+07
Q9GZT3	SRA stem-loop-interacting RNA-binding protein, mitochondrial	24.77	0.427	2	1.02E+07	5	2.81E+07
O75531	Barrier-to-autointegration factor	24.72	0.282	20	2.76E+07	3	1.23E+07
Q9Y5L4	Mitochondrial import inner membrane translocase subunit Tim13	24.21	0.513	6	3.28E+07	2	1.18E+07
Q14118	Dystroglycan	23.46	0.810	8	3.05E+07	2	3.45E+07
J3KN29	26S proteasome non-ATPase regulatory subunit 9	23.42	0.632	10	2.99E+07	1	1
Q9NUP9	Protein lin-7 homolog C	23.35	0.674	2	1.14E+08	2	1.78E+07
P33316	Deoxyuridine 5'-triphosphate nucleotidohydrolase, mitochondrial	22.62	0.571	12	1.82E+07	3	1

Accession	Protein Name	RAPID % Disorder	SLIDER IDR propensity score (0-1)	Total Spectrum Count Sample 1	Average Precursor Intensity Sample 1	Total Spectrum Count Sample 2	Average Precursor Intensity Sample 2
P61604	10 kDa heat shock protein, mitochondrial	22.55	0.254	27	1.54E+08	29	5.45E+07
Q9Y5J7	Mitochondrial import inner membrane translocase subunit Tim9	22.47	0.422	11	2.97E+07	1	3.75E+07
A8MWD9	Putative small nuclear ribonucleoprotein G-like protein 15	22.37	0.296	5	9257000	2	1.51E+07
P62942	Peptidyl-prolyl cis-trans isomerase FKBP1A	22.22	0.298	9	5.46E+07	4	3.68E+07
P50238	Cysteine-rich protein 1	22.08	0.260	9	2.07E+08	6	7.69E+07
Q15819	Ubiquitin-conjugating enzyme E2 variant 2	22.07	0.547	5	2.40E+07	1	3.82E+07
P27348	14-3-3 protein theta	22.04	0.670	24	4.29E+07	1	1.81E+08
P61978	Heterogeneous nuclear ribonucleoprotein K	22.03	0.801	60	1.05E+08	10	1.54E+07
P21291	Cysteine and glycine-rich protein 1	21.76	0.396	4	4.64E+07	1	1.15E+07
F8W1A4	Adenylate kinase 2, mitochondrial	21.55	0.594	16	1.65E+07	3	3477400
E9PN81	Ribonuclease H2 subunit C	21.46	0.564	3	9735000	1	1
P31946	14-3-3 protein beta/alpha	21.14	0.664	45	4.32E+07	3	2.13E+07
Q9Y2Z0	Protein SGT1 homolog	21.1	0.683	8	3.79E+07	1	1.82E+07
Q8NFU3	Thiosulfate:glutathione sulfurtransferase	20.87	0.542	8	1.28E+08	8	5.23E+07
Q14134	Tripartite motif-containing protein 29	20.75	0.864	21	4.04E+07	2	3.05E+07
A0A0B4J1Z1	Serine/arginine-rich-splicing factor 7	20.44	0.340	12	6.83E+07	4	1.93E+07
A0A0A0MRL6	Actin-binding LIM protein 1	20.38	0.806	15	2.21E+07	3	1.38E+07
E9PMI6	Chloride channel, nucleotide sensitive 1A	20.36	0.456	9	3.72E+07	1	1744500
J3KS15	Peptidyl-tRNA hydrolase ICT1, mitochondrial (Fragment)	20.31	0.560	2	8655200	1	6644600
O43678	NADH dehydrogenase [ubiquinone] 1 alpha subcomplex subunit 2	20.2	0.461	3	1.37E+07	7	2.68E+07
P52943	Cysteine-rich protein 2	20.19	0.512	13	1.02E+08	7	3.21E+07
G3V4P8	Glia maturation factor beta (Fragment)	20	0.482	11	2.99E+07	2	1513600
P14854	Cytochrome c oxidase subunit 6B1	19.77	0.261	12	7.88E+07	10	8.64E+07

Accession	Protein Name	RAPID % Disorder	SLIDER IDR propensity score (0-1)	Total Spectrum Count Sample 1	Average Precursor Intensity Sample 1	Total Spectrum Count Sample 2	Average Precursor Intensity Sample 2
Q9BWJ5	Splicing factor 3B subunit 5	19.77	0.351	6	1.58E+07	1	6719700
A0A0A0MTS7	Titin	19.74	0.937	2	5.75E+07	1	813140
Q15293	Reticulocalbin-1	19.64	0.676	12	8.49E+07	3	2.38E+07
P62273	40S ribosomal protein S29	19.64	0.154	3	3.70E+07	2	7.46E+07
Q92882	Osteoclast-stimulating factor 1	19.63	0.514	18	4.29E+07	2	1225500
P37802	Transgelin-2	19.6	0.482	48	6.51E+07	13	8563200
P30050	60S ribosomal protein L12	19.39	0.479	8	2.41E+07	3	9722000
P05787	Keratin, type II cytoskeletal 8	19.25	0.827	10	2.22E+07	3	4.23E+07
Q8WVJ2	NudC domain-containing protein 2	19.11	0.360	5	1.83E+07	1	1.07E+07
A0A1W2PQV2	Glycine cleavage system H protein, mitochondrial (Fragment)	19	0.251	4	2.02E+07	1	1.21E+07
P61758	Prefoldin subunit 3	18.78	0.604	11	2.62E+07	1	1
Q4VC31	Coiled-coil domain-containing protein 58	18.75	0.538	11	3.89E+07	4	1042000
H0Y5E4	CD44 antigen (Fragment)	18.71	0.546	4	9.15E+07	4	3.57E+07
Q5JTJ3	Cytochrome c oxidase assembly factor 6 homolog	18.4	0.481	7	4.33E+07	6	2.91E+07
O43598	2'-deoxynucleoside 5'-phosphate N-hydrolase 1	18.39	0.534	5	3.12E+07	1	9577700
P47929	Galectin-7	18.38	0.277	28	1.46E+08	16	3.40E+07
P52815	39S ribosomal protein L12, mitochondrial	18.18	0.631	11	1.90E+07	2	5294100
P23528	Cofilin-1	18.07	0.459	81	1.31E+08	26	2.61E+07
P17931	Galectin-3	18	0.515	14	6.27E+07	8	2.84E+07
Q9NRV9	Heme-binding protein 1	17.99	0.400	6	1.49E+07	1	1.55E+07
Q13308	Inactive tyrosine-protein kinase 7	17.66	0.737	2	5994000	1	1
Q96FQ6	Protein S100-A16	17.48	0.369	25	9.98E+07	4	4177500
Q04917	14-3-3 protein eta	17.48	0.608	8	1.15E+08	2	6.68E+07
Q9UK45	U6 snRNA-associated Sm-like protein LSm7	17.48	0.407	3	1.68E+07	2	1.56E+07

Accession	Protein Name	RAPID % Disorder	SLIDER IDR propensity score (0-1)	Total Spectrum Count Sample 1	Average Precursor Intensity Sample 1	Total Spectrum Count Sample 2	Average Precursor Intensity Sample 2
Q99757	Thioredoxin, mitochondrial	17.47	0.371	3	2.33E+07	2	4.31E+07
P38646	Stress-70 protein, mitochondrial	17.38	0.773	16	2.24E+07	1	1
P16188	P16188	17.26	0.626	4	1.00E+07	1	4.55E+07
P31949	Protein S100-A11	17.14	0.340	92	1.17E+08	22	1.62E+07
P30101	Protein disulfide-isomerase A3	17.03	0.629	52	5.83E+07	7	2.31E+07
A0A087X0R6	Sorting nexin-12	16.86	0.432	10	1.72E+07	3	8999600
P41567	Eukaryotic translation initiation factor 1	16.81	0.347	6	2.92E+07	2	4714300
P60903	Protein S100-A10	16.49	0.302	19	5.72E+07	1	4.07E+07
P84090	Enhancer of rudimentary homolog	16.35	0.323	12	9.45E+07	7	7.60E+07
A0A075B7D9	TATA-binding protein-associated factor 2N	16.14	0.545	8	3.39E+07	2	4.81E+07
Q9NVS9	Pyridoxine-5'-phosphate oxidase	16.09	0.463	8	1.65E+07	1	1273200
P13984	General transcription factor IIF subunit 2	16.06	0.522	5	3.65E+07	1	1
O43181	NADH dehydrogenase [ubiquinone] iron-sulfur protein 4, mitochondrial	16	0.619	2	2.06E+07	2	1.77E+07
O43504	Ragulator complex protein LAMTOR5	15.38	0.265	2	1.65E+07	1	3.09E+07
P08729	Keratin, type II cytoskeletal 7	15.35	0.837	8	1.35E+07	3	4.10E+07
P20674	Cytochrome c oxidase subunit 5A, mitochondrial	15.33	0.424	12	1.26E+08	12	9.14E+07
P29373	Cellular retinoic acid-binding protein 2	15.22	0.412	12	4.05E+07	1	5964100
P11021	Endoplasmic reticulum chaperone BiP	15.14	0.750	39	5.01E+07	2	7320000
Q9Y281	Cofilin-2	15.06	0.415	25	9.83E+07	10	2.00E+07
P02533	Keratin, type I cytoskeletal 14	14.83	0.767	77	9.15E+07	34	4.49E+07
P06703	Protein S100-A6	14.44	0.453	13	3.27E+07	2	1.30E+07
P40121	Macrophage-capping protein	14.37	0.496	22	3.88E+07	2	3670400
P10599	Thioredoxin	14.29	0.235	99	4.47E+08	7	2.32E+07
P30085	UMP-CMP kinase	14.29	0.467	24	6.62E+07	5	3853900

Accession	Protein Name	RAPID % Disorder	SLIDER IDR propensity score (0-1)	Total Spectrum Count Sample 1	Average Precursor Intensity Sample 1	Total Spectrum Count Sample 2	Average Precursor Intensity Sample 2
P12830	Cadherin-1	14.29	0.697	24	5.80E+07	2	9746300
Q14126	Desmoglein-2	14.13	0.737	21	2.42E+07	3	1.11E+07
P55769	NHP2-like protein 1	14.06	0.465	4	3.25E+07	2	1.56E+07
P60981	Destrin	13.94	0.376	20	4.55E+07	4	2.40E+07
P30086	Phosphatidylethanolamine-binding protein 1	13.9	0.335	32	1.05E+08	10	3.64E+07
P32320	Cytidine deaminase	13.7	0.302	20	8.27E+07	4	3.34E+07
A0A0A0MR02	Outer mitochondrial membrane protein porin 2 (Fragment)	13.48	0.346	1	1.49E+07	2	7848300
Q04695	Keratin, type I cytoskeletal 17	13.43	0.768	61	8.07E+07	27	4.05E+07
P55072	Transitional endoplasmic reticulum ATPase	13.4	0.757	4	6141400	1	1
A0A087WXM8	Basal cell adhesion molecule	13.27	0.664	30	5.13E+07	10	1.08E+07
P63220	40S ribosomal protein S21	13.25	0.223	17	4.61E+07	10	3.67E+07
O60493	Sorting nexin-3	12.96	0.394	7	4.49E+07	2	6.41E+07
P25398	40S ribosomal protein S12	12.88	0.304	32	1.62E+08	18	1.34E+07
P28799	Progranulin	12.82	0.423	17	7.17E+07	1	4.80E+07
Q01469	Fatty acid-binding protein 5	12.59	0.350	13	4.29E+07	1	6505900
H0YKS4	Annexin (Fragment)	12.5	0.529	12	2.39E+07	1	1.11E+07
F8VZY9	Keratin, type I cytoskeletal 18	12.28	0.752	9	3.48E+07	1	1.42E+08
Q9Y4Y9	U6 snRNA-associated Sm-like protein LSm5	12.09	0.205	3	7555600	1	4124500
A0A1B0GU03	Peptidase A1 domain-containing protein	12.03	0.650	2	2.20E+07	5	1.12E+07
P54652	Heat shock-related 70 kDa protein 2	11.89	0.710	17	3.19E+07	2	5883000
P22307	Non-specific lipid-transfer protein	11.88	0.593	12	3.08E+07	1	4.49E+07
P63241	Eukaryotic translation initiation factor 5A-1	11.69	0.458	47	7.53E+07	5	1.06E+07
P13647	Keratin, type II cytoskeletal 5	11.69	0.792	15	3.22E+07	13	5.18E+07
P68104	Elongation factor 1-alpha 1	11.47	0.528	12	4.40E+07	3	4.56E+07

Accession	Protein Name	RAPID % Disorder	SLIDER IDR propensity score (0-1)	Total Spectrum Count Sample 1	Average Precursor Intensity Sample 1	Total Spectrum Count Sample 2	Average Precursor Intensity Sample 2
Q15223	Nectin-1	11.41	0.668	3	2.20E+07	1	6175000
P62633	Cellular nucleic acid-binding protein	11.3	0.436	17	8.30E+07	9	3.27E+07
Q99497	Parkinson disease protein 7	11.11	0.377	41	8.29E+07	5	1.10E+07
P49773	Histidine triad nucleotide-binding protein 1	11.11	0.253	22	1.72E+08	15	5.43E+07
P11142	Heat shock cognate 71 kDa protein	10.99	0.741	29	6.06E+07	2	5883000
Q96NY8	Nectin-4	10.98	0.734	7	1.91E+07	1	4997600
P62937	Peptidyl-prolyl cis-trans isomerase A	10.91	0.234	95	3.88E+08	45	1.24E+08
P28072	Proteasome subunit beta type-6	10.88	0.463	2	1.55E+07	4	2.93E+07
P17066	Heat shock 70 kDa protein 6	10.73	0.735	9	7.62E+07	1	1
P07602	Prosaposin	10.69	0.579	30	6.69E+07	2	9363900
D3YTI2	Acid phosphatase	10.59	0.191	3	4.00E+07	1	2.06E+07
Q06830	Peroxiredoxin-1	10.55	0.263	74	4.24E+08	19	4.29E+07
G8JLA2	Myosin light polypeptide 6	10.53	0.311	22	1.08E+08	11	1.98E+07
P02538	Keratin, type II cytoskeletal 6A	10.46	0.762	8	2.08E+07	3	3.30E+07
P28066	Proteasome subunit alpha type-5	10.37	0.414	3	1685000	1	2.96E+07
P52907	F-actin-capping protein subunit alpha-1	10.14	0.551	10	3.57E+07	1	5.07E+07
B1AKR6	Dynein light chain roadblock-type 1	10.14	0.376	8	3.90E+07	1	9.32E+07
Q99436	Proteasome subunit beta type-7	10.11	0.522	6	1.57E+07	1	6771000
Q9BUF5	Tubulin beta-6 chain	9.87	0.588	2	6426800	1	1.13E+07
P30044	Peroxiredoxin-5, mitochondrial	9.81	0.402	40	1.59E+08	10	1.95E+07
Q9BRA2	Thioredoxin domain-containing protein 17	9.76	0.231	16	1.31E+08	8	3.77E+07
P62993	Growth factor receptor-bound protein 2	9.68	0.363	5	4.06E+07	1	1.11E+07
P14174	Macrophage migration inhibitory factor	9.57	0.260	8	6.03E+07	4	3.99E+07
P21796	Voltage-dependent anion-selective channel protein 1	9.54	0.360	3	1.64E+07	1	2.77E+07

Accession	Protein Name	RAPID % Disorder	SLIDER IDR propensity score (0-1)	Total Spectrum Count Sample 1	Average Precursor Intensity Sample 1	Total Spectrum Count Sample 2	Average Precursor Intensity Sample 2
A0A0G2JIW1	Heat shock 70 kDa protein 1B	9.5	0.720	27	3.35E+07	1	4463400
O60888	Protein CutA	9.5	0.449	4	7770200	2	3911600
P60660	Myosin light polypeptide 6	9.27	0.288	27	8.59E+07	14	1.77E+07
Q15365	Poly(rC)-binding protein 1	9.27	0.467	20	5.55E+07	1	4969900
K7ENE5	Deoxyribonuclease II (Fragment)	9.17	0.359	1	2.36E+07	2	5.71E+07
Q9NRX4	14 kDa phosphohistidine phosphatase	8.8	0.290	13	4.16E+07	10	2.16E+07
P10809	60 kDa heat shock protein, mitochondrial	8.73	0.585	17	1.61E+07	1	1.42E+07
P18510	Interleukin-1 receptor antagonist protein	8.47	0.382	11	4.31E+07	2	1.30E+07
Q9Y2B0	Protein canopy homolog 2	8.24	0.479	15	4.41E+07	5	1.47E+07
H3BQ34	Pyruvate kinase	8.19	0.423	6	1.22E+07	2	1
E7EUT5	Glyceraldehyde-3-phosphate dehydrogenase	8.08	0.341	7	2.98E+07	5	1.00E+07
P04075	Fructose-bisphosphate aldolase A	7.97	0.557	31	4.02E+07	2	3.31E+07
C9J0K6	Sorcin	7.74	0.276	17	5.50E+07	4	1.49E+07
P60174	Triosephosphate isomerase	7.63	0.269	79	2.89E+08	40	5.64E+07
P24666	Low molecular weight phosphotyrosine protein phosphatase	7.59	0.400	10	3.99E+07	1	2.06E+07
P35754	Glutaredoxin-1	7.55	0.192	4	2.25E+07	1	1.62E+07
Q04837	Single-stranded DNA-binding protein, mitochondrial	7.43	0.311	8	1.35E+07	4	8474000
Q9Y536	Peptidyl-prolyl cis-trans isomerase A-like 4A	7.32	0.253	14	1.26E+09	8	7.31E+07
Q15181	Inorganic pyrophosphatase	7.27	0.403	32	5.82E+07	2	8640600
Q7RTV0	PHD finger-like domain-containing protein 5A	7.27	0.187	4	2.75E+07	2	1.69E+07
P52597	Heterogeneous nuclear ribonucleoprotein F	7.23	0.541	22	6.19E+07	1	2034600
Q13740	CD166 antigen	7.03	0.627	7	1.00E+07	2	8711800
P05187	Alkaline phosphatase, placental type	6.92	0.632	8	9980700	1	3.92E+07
P61769	Beta-2-microglobulin	6.72	0.323	8	6.30E+07	2	1.04E+08

Accession	Protein Name	RAPID % Disorder	SLIDER IDR propensity score (0-1)	Total Spectrum Count Sample 1	Average Precursor Intensity Sample 1	Total Spectrum Count Sample 2	Average Precursor Intensity Sample 2
P68032	Actin, alpha cardiac muscle 1	6.63	0.428	10	4.99E+07	7	3.65E+07
P60709	Actin, cytoplasmic 1	6.4	0.420	11	5.81E+07	7	3.65E+07
Q562R1	Beta-actin-like protein 2	6.12	0.400	4	3.76E+07	2	1.80E+07
P32119	Peroxiredoxin-2	6.06	0.256	43	2.34E+08	11	1.50E+07
P09382	Galectin-1	5.93	0.247	21	1.29E+08	9	3.79E+07
P06733	Alpha-enolase	5.76	0.438	23	2.29E+07	2	1.32E+07
P07737	Profilin-1	5.71	0.298	58	2.07E+08	14	1.58E+07
P04179	Superoxide dismutase [Mn], mitochondrial	5.41	0.312	15	3.38E+07	1	3381500
A6NNI4	Tetraspanin	5.03	0.244	4	7.52E+07	1	1.74E+07
P0C7P4	Putative cytochrome b-c1 complex subunit Rieske-like protein 1	4.95	0.541	2	1.80E+07	1	7.28E+07
O43278	Kunitz-type protease inhibitor 1	4.73	0.527	23	5.11E+07	5	1.87E+07
H7BZJ3	Protein disulfide-isomerase A3 (Fragment)	4.08	0.442	10	1.62E+08	4	4.21E+07
P13639	Elongation factor 2	3.85	0.645	3	2754600	1	894130
P55290	Cadherin-13	3.79	0.545	9	1.75E+07	4	3.73E+07
P09758	Tumor-associated calcium signal transducer 2	3.72	0.645	16	9.66E+07	13	3.29E+07
O15031	Plexin-B2	3.54	0.727	32	1.89E+07	4	6525600
Q9Y624	Junctional adhesion molecule A	3.34	0.506	5	9185400	1	9431900
H7BZ14	Peptidyl-prolyl cis-trans isomerase (Fragment)	3.33	0.236	4	2.04E+07	1	2788900
P26006	Integrin alpha-3	3.33	0.662	4	1.37E+07	1	5966100
P07858	Cathepsin B	2.65	0.313	8	4.81E+07	5	2.77E+07
P23229	Integrin alpha-6	2.57	0.643	12	2.05E+07	2	2.70E+07
O75380	NADH dehydrogenase [ubiquinone] iron-sulfur protein 6, mitochondrial	2.42	0.291	4	4.51E+07	7	5.36E+07
P05556	Integrin beta-1	1.63	0.653	9	1.24E+07	4	9826700
