# Supplementary material for: Thermostable Proteins from HaCaT Keratinocytes Identify a Wide Breadth of Intrinsically Disordered Proteins and Candidates for Liquid–Liquid Phase Separation
Source: Int J Mol Sci. 2022 Nov 18;23(22):14323. doi: 10.3390/ijms232214323 (PMC9692912; doi:10.3390/ijms232214323)
Supplement: Supplementary file 1 [file ijms-23-14323-s001.zip › Supplementary Table S2 NOV08.pdf]

Supplementary Table S2. Additional and overlapping terms ≥10 genes returned for thermostable lysate proteins from DAVID GO. Ordered by protein count per term based on the number of identified genes corresponding to a term.

Molecular Function								
**GO #**	**Term**	**Count**	**%**	**PValue**	**Fold Enrichment**	**Bonferroni**	**Benjamini**	**FDR**
GO:0005488	Binding	293	96.07	4.76E-11	1.10	2.50E-08	2.77E-09	2.50E-09
GO:0005515	Protein binding*	284	93.11	1.02E-22	1.27	5.37E-20	1.79E-20	1.62E-20
GO:0097159	Organic cyclic compound binding*	162	53.11	1.12E-12	1.58	5.88E-10	7.35E-11	6.63E-11
GO:1901363	Heterocyclic compound binding	161	52.79	7.98E-13	1.59	4.18E-10	6.40E-11	5.78E-11
GO:0003676	Nucleic acid binding*	158	51.80	1.02E-28	2.27	5.36E-26	2.68E-26	2.42E-26
GO:0003723	RNA binding*	140	45.90	1.17E-56	4.35	6.13E-54	6.13E-54	5.53E-54
GO:0019899	Enzyme binding*	73	23.93	3.65E-10	2.14	1.91E-07	1.91E-08	1.72E-08
GO:0042802	Identical protein binding*	61	20.00	8.41E-06	1.78	0.004398882	2.11E-04	1.90E-04
GO:0044877	Macromolecular complex binding*	43	14.10	2.17E-05	2.00	0.011292114	4.94E-04	4.46E-04
GO:0003729	mRNA binding*	41	13.44	9.10E-15	4.35	4.77E-12	9.53E-13	8.61E-13
GO:0008092	Cytoskeletal protein binding*	38	12.46	2.46E-06	2.33	0.001287012	8.59E-05	7.75E-05
GO:0050839	Cell adhesion molecule binding*	36	11.80	8.55E-13	4.28	4.48E-10	6.40E-11	5.78E-11
GO:0045296	Cadherin binding*	34	11.15	2.25E-17	6.51	1.18E-14	2.95E-15	2.67E-15
GO:0005198	Structural molecule activity	34	11.15	4.59E-06	2.42	0.002401809	1.41E-04	1.28E-04
GO:0003682	Chromatin binding	30	9.84	1.39E-07	3.11	7.27E-05	5.37E-06	4.85E-06
GO:0019904	Protein domain specific binding	30	9.84	4.87E-06	2.60	0.002548007	1.42E-04	1.28E-04
GO:0003712	Transcription cofactor activity	28	9.18	5.80E-08	3.42	3.04E-05	2.63E-06	2.38E-06
GO:0000989	Transcription factor activity, transcription factor binding	28	9.18	6.03E-08	3.41	3.16E-05	2.63E-06	2.38E-06
GO:0000988	Transcription factor activity, protein binding	28	9.18	1.43E-07	3.27	7.52E-05	5.37E-06	4.85E-06
GO:0003779	Actin binding	22	7.21	1.38E-05	3.03	0.007200006	3.28E-04	2.96E-04
GO:0003713	Transcription coactivator activity	16	5.25	5.11E-05	3.54	0.026444565	0.001116659	0.001007977
GO:0003730	mRNA 3'-UTR binding	13	4.26	3.56E-04	3.51	0.170293261	0.006221657	0.005616114
GO:0003697	Single-stranded DNA binding	12	3.93	3.69E-06	6.22	0.001929092	1.21E-04	1.09E-04
GO:0051082	Unfolded protein binding	12	3.93	6.79E-06	5.84	0.003553482	1.87E-04	1.69E-04
GO:0031072	Heat shock protein binding	12	3.93	8.45E-06	5.71	0.004415504	2.11E-04	1.90E-04
GO:0043021	Ribonucleoprotein complex binding	11	3.61	1.95E-04	4.44	0.097196386	0.003651438	0.00329605
GO:0051087	Chaperone binding	10	3.28	5.75E-05	5.80	0.029702384	0.001159676	0.001046806
GO:0008135	Translation factor activity, RNA binding	10	3.28	5.75E-05	5.80	0.029702384	0.001159676	0.001046806

Cellular Component								
**GO #**	**Term**	**Count**	**%**	**PValue**	**Fold Enrichment**	**Bonferroni**	**Benjamini**	**FDR**
GO:0044464	Cell part	291	95.41	1.19E-09	1.08	7.85E-07	2.53E-08	2.18E-08
GO:0005623	Cell	291	95.41	1.19E-09	1.08	7.85E-07	2.53E-08	2.18E-08
GO:0044424	Intracellular part	289	94.75	1.46E-24	1.24	9.68E-22	1.08E-22	9.25E-23
GO:0005622	Intracellular*	289	94.75	1.65E-24	1.24	1.09E-21	1.09E-22	9.37E-23
GO:0043226	Organelle*	275	90.16	7.67E-20	1.28	5.08E-17	3.38E-18	2.91E-18
GO:0043229	Intracellular organelle	271	88.85	3.05E-23	1.34	2.02E-20	1.84E-21	1.58E-21
GO:0043227	Membrane-bounded organelle	259	84.92	8.11E-17	1.30	7.35E-14	2.24E-15	1.92E-15
GO:0043231	Intracellular membrane-bounded organelle	238	78.03	3.64E-15	1.35	2.43E-12	9.27E-14	7.97E-14
GO:0044422	Organelle part	232	76.07	5.89E-19	1.45	3.90E-16	2.17E-17	1.86E-17
GO:0005737	Cytoplasm	232	76.07	4.55E-12	1.31	3.01E-09	1.06E-10	9.08E-11
GO:0044446	Intracellular organelle part	231	75.74	1.73E-20	1.49	1.15E-17	8.82E-19	7.58E-19
GO:0005634	Nucleus*	215	70.49	2.04E-29	1.78	1.35E-26	6.76E-27	5.81E-27
GO:0044444	Cytoplasmic part	193	63.28	9.78E-08	1.30	6.47E-05	1.58E-06	1.36E-06
GO:0070013	Intracellular organelle lumen	175	57.38	2.57E-25	1.96	1.70E-22	2.12E-23	1.83E-23
GO:0043233	Organelle lumen*	175	57.38	2.57E-25	1.96	1.70E-22	2.12E-23	1.83E-23
GO:0031974	Membrane-enclosed lumen	175	57.38	2.57E-25	1.96	1.70E-22	2.12E-23	1.83E-23
GO:0032991	Macromolecular complex*	173	56.72	9.11E-22	1.85	6.03E-19	5.03E-20	4.32E-20
GO:0044428	Nuclear part*	169	55.41	1.53E-28	2.14	1.01E-25	3.37E-26	2.90E-26
GO:0031981	Nuclear lumen	160	52.46	1.53E-27	2.19	1.01E-24	2.53E-25	2.17E-25
GO:0005654	Nucleoplasm	156	51.15	2.02E-34	2.55	1.34E-31	1.34E-31	1.15E-31
GO:0005829	Cytosol*	152	49.84	1.09E-19	1.92	7.21E-17	4.51E-18	3.88E-18
GO:0043232	Intracellular non-membrane-bounded organelle	151	49.51	3.78E-17	1.82	2.50E-14	1.14E-15	9.78E-16
GO:0043228	Non-membrane-bounded organelle	151	49.51	4.38E-17	1.82	2.90E-14	1.26E-15	1.08E-15
GO:0043234	Protein complex*	110	36.07	9.47E-19	2.32	6.27E-16	3.30E-17	2.84E-17
GO:0005856	Cytoskeleton	71	23.28	6.18E-09	2.03	4.09E-06	1.20E-07	1.03E-07
GO:1990904	Ribonucleoprotein complex*	67	21.97	1.62E-18	3.36	1.07E-15	5.37E-17	4.61E-17
GO:0044451	Nucleoplasm part	63	20.66	1.00E-17	3.42	6.64E-15	3.16E-16	2.72E-16
GO:0030529	Intracellular ribonucleoprotein complex	62	20.33	1.21E-16	3.29	7.35E-14	3.22E-15	2.77E-15
GO:0070062	Extracellular exosome	61	20.00	6.70E-07	1.93	4.43E-04	1.01E-05	8.66E-06
GO:1903561	Extracellular vesicle	61	20.00	2.73E-06	1.85	0.00180435	4.01E-05	3.45E-05
GO:0043230	Extracellular organelle	61	20.00	2.80E-06	1.85	0.001855036	4.04E-05	3.47E-05

GO:0016604	Nuclear body*	54	17.70	1.72E-19	4.33	1.14E-16	6.68E-18	5.74E-18
GO:0044430	Cytoskeletal part	52	17.05	5.83E-06	1.93	0.00385044	8.04E-05	6.91E-05
GO:0005694	Chromosome	50	16.39	4.00E-05	1.83	0.026164363	5.00E-04	4.30E-04
GO:0044427	Chromosomal part	43	14.10	4.62E-04	1.74	0.263447535	0.00449565	0.003864086
GO:1902494	Catalytic complex	41	13.44	1.88E-04	1.85	0.116821853	0.002036339	0.001750267
GO:0016607	Nuclear speck*	40	13.11	2.41E-20	6.49	1.59E-17	1.14E-18	9.78E-19
GO:0030054	Cell junction	39	12.79	1.73E-04	1.90	0.108307171	0.001910394	0.001642015
GO:0005681	Spliceosomal complex*	35	11.48	7.76E-27	12.27	5.14E-24	1.03E-24	8.83E-25
GO:0070161	Anchoring junction	33	10.82	5.61E-05	2.17	0.036440574	6.87E-04	5.91E-04
GO:0099512	Supramolecular fiber	31	10.16	2.30E-04	2.07	0.141190008	0.002415716	0.002076348
GO:0099081	Supramolecular polymer	31	10.16	2.60E-04	2.05	0.158379235	0.002652359	0.002279746
GO:0099080	Supramolecular complex	31	10.16	2.71E-04	2.05	0.164148962	0.002680597	0.002304017
GO:0015629	Actin cytoskeleton	29	9.51	3.10E-09	3.81	2.05E-06	6.41E-08	5.51E-08
GO:0005912	Adherens junction	29	9.51	1.28E-08	3.57	8.47E-06	2.29E-07	1.97E-07
GO:0030055	Cell-substrate junction	26	8.52	3.60E-09	4.20	2.38E-06	7.21E-08	6.20E-08
GO:0005925	Focal adhesion	25	8.20	1.06E-08	4.13	6.99E-06	2.00E-07	1.72E-07
GO:0005924	Cell-substrate adherens junction	25	8.20	1.26E-08	4.10	8.36E-06	2.29E-07	1.97E-07
GO:0071013	Catalytic step 2 spliceosome	18	5.90	9.16E-15	14.02	6.03E-12	2.25E-13	1.93E-13
GO:0005635	Nuclear envelope	18	5.90	7.75E-04	2.55	0.401497988	0.007227133	0.006211841
GO:0099568	Cytoplasmic region	17	5.57	2.71E-04	2.91	0.164415951	0.002680597	0.002304017
GO:0005684	U2-type spliceosomal complex	16	5.25	4.63E-12	11.93	3.06E-09	1.06E-10	9.08E-11
GO:0035770	Ribonucleoprotein granule	15	4.92	3.63E-05	3.88	0.023775247	4.63E-04	3.98E-04
GO:0036464	Cytoplasmic ribonucleoprotein granule	14	4.59	7.26E-05	3.88	0.046913979	8.28E-04	7.12E-04
GO:0044449	Contractile fiber part	13	4.26	1.33E-04	3.91	0.084570859	0.001497566	0.001287183
GO:0043292	Contractile fiber	13	4.26	2.39E-04	3.68	0.146415388	0.002473307	0.002125849
GO:0032432	Actin filament bundle	12	3.93	2.27E-08	10.26	1.50E-05	3.95E-07	3.40E-07
GO:0030016	Myofibril	12	3.93	6.56E-04	3.52	0.352230668	0.006201118	0.005329964
GO:0097517	Contractile actin filament bundle	11	3.61	8.14E-08	10.56	5.39E-05	1.35E-06	1.16E-06
GO:0001725	Stress fiber	11	3.61	8.14E-08	10.56	5.39E-05	1.35E-06	1.16E-06
GO:0042641	Actomyosin	11	3.61	2.81E-07	9.29	1.86E-04	4.33E-06	3.72E-06
GO:0034399	Nuclear periphery	11	3.61	6.93E-05	5.04	0.04485102	8.05E-04	6.92E-04
GO:0001533	Cornified envelope	10	3.28	1.41E-07	11.88	9.35E-05	2.23E-06	1.91E-06
GO:0010494	Cytoplasmic stress granule	10	3.28	4.07E-06	8.06	0.002692227	5.74E-05	4.93E-05
GO:0005884	Actin filament	10	3.28	6.28E-05	5.75	0.040732116	7.43E-04	6.38E-04

Biological Process								
GO #	Term	Count	%	PValue	Fold Enrichment	Bonferroni	Benjamini	FDR
GO:0009987	Cellular process	274	89.84	5.40E-06	1.07	0.019681368	1.84E-04	1.73E-04
GO:0071704	Organic substance metabolic process	208	68.20	2.76E-04	1.15	0.63769521	0.00634456	0.005962233
GO:0044238	Primary metabolic process	206	67.54	3.23E-05	1.19	0.112327493	9.03E-04	8.48E-04
GO:0044237	Cellular metabolic process	204	66.89	1.21E-04	1.17	0.359961105	0.003098606	0.002911882
GO:0043170	Macromolecule metabolic process	203	66.56	4.00E-09	1.30	1.47E-05	3.20E-07	3.01E-07
GO:0044260	Cellular macromolecule metabolic process	196	64.26	1.61E-10	1.36	5.93E-07	1.74E-08	1.64E-08
GO:0034641	Cellular nitrogen compound metabolic process	171	56.07	7.19E-12	1.50	2.65E-08	9.81E-10	9.22E-10
GO:0006807	Nitrogen compound metabolic process	171	56.07	1.07E-09	1.42	3.94E-06	9.50E-08	8.92E-08
GO:0071840	Cellular component organization or biogenesis	161	52.79	2.04E-12	1.56	7.52E-09	3.13E-10	2.94E-10
GO:0010467	Gene expression*	159	52.13	9.60E-14	1.62	3.54E-10	1.97E-11	1.85E-11
GO:0016043	Cellular component organization	159	52.13	9.04E-13	1.58	3.33E-09	1.51E-10	1.42E-10
GO:1901360	Organic cyclic compound metabolic process	154	50.49	1.24E-10	1.52	4.58E-07	1.43E-08	1.35E-08
GO:0006139	Nucleobase-containing compound metabolic process	153	50.16	6.46E-13	1.62	2.38E-09	1.19E-10	1.12E-10
GO:0046483	Heterocycle metabolic process	153	50.16	6.76E-12	1.58	2.49E-08	9.58E-10	9.01E-10
GO:0006725	Cellular aromatic compound metabolic process	153	50.16	1.45E-11	1.56	5.34E-08	1.78E-09	1.67E-09
GO:0090304	Nucleic acid metabolic process*	149	48.85	2.68E-15	1.74	9.82E-12	8.24E-13	7.74E-13
GO:0019222	Regulation of metabolic process	148	48.52	2.68E-06	1.35	0.009816358	1.01E-04	9.46E-05
GO:0060255	Regulation of macromolecule metabolic process	145	47.54	1.08E-07	1.43	3.98E-04	6.12E-06	5.75E-06
GO:0080090	Regulation of primary metabolic process	141	46.23	5.65E-07	1.40	0.002078609	2.67E-05	2.51E-05
GO:0031323	Regulation of cellular metabolic process	141	46.23	1.99E-06	1.38	0.007321247	8.07E-05	7.59E-05
GO:0016070	RNA metabolic process*	139	45.57	5.03E-15	1.80	1.84E-11	1.32E-12	1.24E-12
GO:0044249	Cellular biosynthetic process	126	41.31	4.27E-04	1.28	0.792318976	0.009353671	0.008790013
GO:1901576	Organic substance biosynthetic process	126	41.31	6.25E-04	1.27	0.900091169	0.012793207	0.012022281
GO:0034645	Cellular macromolecule biosynthetic process	124	40.66	9.83E-08	1.51	3.62E-04	5.66E-06	5.32E-06

GO:0009059	Macromolecule biosynthetic process	124	40.66	1.97E-07	1.49	7.27E-04	1.02E-05	9.62E-06
GO:0048519	Negative regulation of biological process	123	40.33	1.01E-05	1.40	0.036481038	3.23E-04	3.03E-04
GO:0044271	Cellular nitrogen compound biosynthetic process	120	39.34	6.56E-07	1.48	0.002414805	3.06E-05	2.88E-05
GO:0051171	Regulation of nitrogen compound metabolic process	118	38.69	3.57E-08	1.56	1.32E-04	2.31E-06	2.17E-06
GO:0010468	Regulation of gene expression	118	38.69	8.31E-08	1.54	3.06E-04	4.88E-06	4.59E-06
GO:0006996	Organelle organization	115	37.70	9.89E-13	1.86	3.64E-09	1.58E-10	1.49E-10
GO:0048523	Negative regulation of cellular process	114	37.38	3.18E-05	1.39	0.110634233	8.95E-04	8.41E-04
GO:0019219	Regulation of nucleobase-containing compound metabolic process	112	36.72	3.52E-11	1.79	1.30E-07	4.19E-09	3.93E-09
GO:0051252	Regulation of RNA metabolic process	103	33.77	1.98E-10	1.81	7.28E-07	2.08E-08	1.95E-08
GO:0031326	Regulation of cellular biosynthetic process	103	33.77	5.12E-05	1.42	0.171768455	0.001375604	0.001292709
GO:0009889	Regulation of biosynthetic process	103	33.77	8.73E-05	1.40	0.275029169	0.002280921	0.002143471
GO:2000112	Regulation of cellular macromolecule biosynthetic process	102	33.44	1.02E-05	1.48	0.036729954	3.23E-04	3.03E-04
GO:0010556	Regulation of macromolecule biosynthetic process	102	33.44	1.52E-05	1.46	0.054396097	4.51E-04	4.24E-04
GO:0018130	Heterocycle biosynthetic process	99	32.46	1.60E-06	1.55	0.00588447	6.78E-05	6.37E-05
GO:0019438	Aromatic compound biosynthetic process	99	32.46	1.71E-06	1.55	0.006270537	7.07E-05	6.64E-05
GO:1901362	Organic cyclic compound biosynthetic process	99	32.46	8.01E-06	1.50	0.02908238	2.64E-04	2.48E-04
GO:0044085	Cellular component biogenesis	98	32.13	8.02E-12	1.96	2.96E-08	1.06E-09	9.92E-10
GO:0034654	Nucleobase-containing compound biosynthetic process	98	32.13	1.29E-06	1.56	0.004740331	5.72E-05	5.38E-05
GO:0022607	Cellular component assembly	94	30.82	2.61E-12	2.04	9.61E-09	3.84E-10	3.61E-10
GO:0097659	Nucleic acid-templated transcription	93	30.49	1.45E-07	1.67	5.34E-04	7.85E-06	7.38E-06
GO:0032774	RNA biosynthetic process	93	30.49	2.05E-07	1.66	7.53E-04	1.03E-05	9.72E-06
GO:0006351	Transcription, DNA-templated	87	28.52	1.23E-06	1.64	0.004508554	5.51E-05	5.18E-05
GO:0006355	Regulation of transcription, DNA-templated	86	28.20	3.49E-06	1.60	0.012782617	1.25E-04	1.17E-04
GO:1903506	Regulation of nucleic acid-templated transcription	86	28.20	3.57E-06	1.60	0.013066859	1.26E-04	1.19E-04

GO:2001141	Regulation of RNA biosynthetic process	86	28.20	4.26E-06	1.59	0.01556316	1.49E-04	1.40E-04
GO:0009892	Negative regulation of metabolic process	85	27.87	1.59E-06	1.64	0.005858155	6.78E-05	6.37E-05
GO:0009893	Positive regulation of metabolic process	85	27.87	6.56E-05	1.49	0.214768135	0.001726916	0.001622851
GO:0010605	Negative regulation of macromolecule metabolic process	84	27.54	1.81E-07	1.73	6.65E-04	9.51E-06	8.93E-06
GO:0031325	Positive regulation of cellular metabolic process	83	27.21	1.36E-05	1.57	0.048948598	4.11E-04	3.87E-04
GO:0010604	Positive regulation of macromolecule metabolic process	82	26.89	1.64E-05	1.57	0.05856826	4.83E-04	4.54E-04
GO:0031324	Negative regulation of cellular metabolic process	77	25.25	1.28E-05	1.61	0.046209342	3.94E-04	3.70E-04
GO:0043933	Macromolecular complex subunit organization	76	24.92	5.87E-14	2.49	2.16E-10	1.27E-11	1.19E-11
GO:0065003	Macromolecular complex assembly*	70	22.95	1.09E-13	2.60	4.01E-10	2.11E-11	1.98E-11
GO:0016071	mRNA metabolic process	68	22.30	2.92E-35	6.47	1.08E-31	1.08E-31	1.01E-31
GO:0006396	RNA processing*	68	22.30	6.58E-18	3.21	2.42E-14	2.20E-15	2.07E-15
GO:0051173	Positive regulation of nitrogen compound metabolic process	68	22.30	8.36E-09	2.05	3.08E-05	5.92E-07	5.57E-07
GO:0051128	Regulation of cellular component organization	68	22.30	4.49E-05	1.62	0.152491298	0.001243988	0.001169025
GO:0045935	Positive regulation of nucleobase-containing compound metabolic process	65	21.31	4.40E-09	2.14	1.62E-05	3.45E-07	3.24E-07
GO:0010628	Positive regulation of gene expression	65	21.31	8.35E-08	1.98	3.08E-04	4.88E-06	4.59E-06
GO:0051172	Negative regulation of nitrogen compound metabolic process	63	20.66	2.98E-06	1.81	0.010933021	1.10E-04	1.03E-04
GO:0010629	Negative regulation of gene expression	63	20.66	1.08E-05	1.74	0.03910887	3.38E-04	3.18E-04
GO:0031328	Positive regulation of cellular biosynthetic process	62	20.33	2.31E-07	1.97	8.52E-04	1.15E-05	1.08E-05
GO:0009891	Positive regulation of biosynthetic process	62	20.33	4.63E-07	1.93	0.001703077	2.21E-05	2.08E-05
GO:0006357	Regulation of transcription from RNA polymerase II promoter	62	20.33	4.47E-04	1.54	0.807301885	0.009639947	0.009059038
GO:0010557	Positive regulation of macromolecule biosynthetic process	60	19.67	1.24E-07	2.04	4.57E-04	6.92E-06	6.50E-06
GO:0006397	mRNA processing*	59	19.34	3.70E-34	7.61	1.36E-30	4.55E-31	4.27E-31
GO:0034622	Cellular macromolecular complex assembly*	59	19.34	1.01E-14	3.11	3.72E-11	2.47E-12	2.32E-12

GO:0006366	Transcription from RNA polymerase II promoter	59	19.34	1.19E-06	1.92	0.004368255	5.40E-05	5.08E-05
GO:0045934	Negative regulation of nucleobase-containing compound metabolic process	57	18.69	1.26E-09	2.38	4.64E-06	1.05E-07	9.91E-08
GO:0051254	Positive regulation of RNA metabolic process	57	18.69	4.98E-08	2.15	1.83E-04	3.11E-06	2.92E-06
GO:0008380	RNA splicing*	55	18.03	3.39E-34	8.49	1.25E-30	4.55E-31	4.27E-31
GO:0008219	Cell death	54	17.70	2.36E-04	1.65	0.581568748	0.005620256	0.005281576
GO:0051253	Negative regulation of RNA metabolic process	53	17.38	2.17E-09	2.45	7.98E-06	1.77E-07	1.67E-07
GO:1902589	Single-organism organelle organization	52	17.05	1.46E-05	1.86	0.052361882	4.37E-04	4.11E-04
GO:0033554	Cellular response to stress	52	17.05	1.04E-04	1.73	0.318493981	0.002700213	0.002537497
GO:0045893	Positive regulation of transcription, DNA-templated	50	16.39	4.98E-06	1.97	0.018176339	1.71E-04	1.61E-04
GO:1903508	Positive regulation of nucleic acid-templated transcription	50	16.39	4.98E-06	1.97	0.018176339	1.71E-04	1.61E-04
GO:1902680	Positive regulation of RNA biosynthetic process	50	16.39	5.73E-06	1.96	0.020892668	1.92E-04	1.80E-04
GO:0012501	Programmed cell death	50	16.39	4.31E-04	1.65	0.795716723	0.009395889	0.008829688
GO:0000375	RNA splicing, via transesterification reactions*	49	16.07	1.29E-33	9.98	4.77E-30	1.19E-30	1.12E-30
GO:0010941	Regulation of cell death	48	15.74	6.00E-05	1.82	0.198372145	0.001590677	0.001494822
GO:0000398	mRNA splicing, via spliceosome*	47	15.41	1.30E-31	9.68	4.79E-28	7.98E-29	7.50E-29
GO:0000377	RNA splicing, via transesterification reactions with bulged adenosine as nucleophile	47	15.41	1.30E-31	9.68	4.79E-28	7.98E-29	7.50E-29
GO:0051276	Chromosome organization	47	15.41	3.57E-08	2.42	1.31E-04	2.31E-06	2.17E-06
GO:0033043	Regulation of organelle organization	46	15.08	1.67E-06	2.14	0.006121024	6.98E-05	6.56E-05
GO:0007010	Cytoskeleton organization	46	15.08	2.84E-06	2.09	0.01039379	1.06E-04	9.92E-05
GO:0045892	Negative regulation of transcription, DNA-templated	44	14.43	2.11E-06	2.17	0.007742282	8.45E-05	7.94E-05
GO:1903507	Negative regulation of nucleic acid-templated transcription	44	14.43	2.19E-06	2.16	0.008041908	8.68E-05	8.16E-05
GO:1902679	Negative regulation of RNA biosynthetic process	44	14.43	3.06E-06	2.14	0.01120167	1.12E-04	1.05E-04
GO:0043603	Cellular amide metabolic process	44	14.43	2.12E-04	1.79	0.541748981	0.00513326	0.004823927
GO:0046907	Intracellular transport	44	14.43	5.86E-04	1.70	0.884583947	0.012195363	0.011460463
GO:0042981	Regulation of apoptotic process	43	14.10	1.60E-04	1.83	0.444463786	0.00397145	0.003732128

GO:0043067	Regulation of programmed cell death	43	14.10	2.24E-04	1.80	0.562361879	0.005400459	0.005075024
GO:0006259	DNA metabolic process	38	12.46	1.85E-06	2.36	0.00680018	7.58E-05	7.12E-05
GO:0045944	Positive regulation of transcription from RNA polymerase II promoter	36	11.80	2.57E-04	1.92	0.612143071	0.005993652	0.005632471
GO:0009057	Macromolecule catabolic process	36	11.80	3.62E-04	1.88	0.736919019	0.008140557	0.007650003
GO:0006325	Chromatin organization	35	11.48	3.14E-07	2.68	0.001156945	1.54E-05	1.45E-05
GO:0006412	Translation	34	11.15	6.15E-04	1.87	0.896346471	0.012659241	0.011896388
GO:1903311	Regulation of mRNA metabolic process*	33	10.82	7.35E-25	11.86	2.71E-21	3.87E-22	3.64E-22
GO:0022613	Ribonucleoprotein complex biogenesis	33	10.82	1.13E-11	4.25	4.18E-08	1.44E-09	1.35E-09
GO:0000122	Negative regulation of transcription from RNA polymerase II promoter	33	10.82	1.86E-05	2.29	0.066266672	5.44E-04	5.11E-04
GO:0044265	Cellular macromolecule catabolic process	32	10.49	2.49E-04	2.03	0.600038695	0.005873548	0.005519605
GO:0010608	Posttranscriptional regulation of gene expression	32	10.49	2.78E-04	2.01	0.640321243	0.006350324	0.005967649
GO:0006886	Intracellular protein transport	31	10.16	5.20E-04	1.97	0.853027175	0.011017291	0.010353383
GO:0043484	Regulation of RNA splicing	30	9.84	7.68E-22	11.19	2.83E-18	3.14E-19	2.95E-19
GO:0022618	Ribonucleoprotein complex assembly	29	9.51	4.35E-15	6.69	1.60E-11	1.23E-12	1.16E-12
GO:0071826	Ribonucleoprotein complex subunit organization	29	9.51	3.07E-14	6.20	1.13E-10	7.08E-12	6.65E-12
GO:0006974	Cellular response to DNA damage stimulus	29	9.51	1.71E-04	2.17	0.466824661	0.004192342	0.003939709
GO:0050684	Regulation of mRNA processing	28	9.18	2.25E-22	13.24	8.30E-19	1.04E-19	9.75E-20
GO:0030029	Actin filament-based process	27	8.85	2.31E-04	2.21	0.572615441	0.005519305	0.005186709
GO:0051052	Regulation of DNA metabolic process	26	8.52	1.56E-06	3.05	0.005735984	6.77E-05	6.36E-05
GO:0010638	Positive regulation of organelle organization	25	8.20	1.24E-04	2.40	0.367251094	0.003156229	0.002966033
GO:0030036	Actin cytoskeleton organization	25	8.20	1.45E-04	2.38	0.414560796	0.003641864	0.003422403
GO:0031329	Regulation of cellular catabolic process	25	8.20	2.55E-04	2.29	0.609399461	0.005986942	0.005626166
GO:0048024	Regulation of mRNA splicing, via spliceosome	24	7.87	2.60E-20	14.79	9.59E-17	9.59E-18	9.01E-18
GO:0071103	DNA conformation change	22	7.21	1.63E-08	4.59	5.99E-05	1.13E-06	1.06E-06
GO:0051493	Regulation of cytoskeleton organization	21	6.89	5.57E-04	2.39	0.871431278	0.011718404	0.011012246
GO:0006403	RNA localization	20	6.56	1.26E-09	5.95	4.62E-06	1.05E-07	9.91E-08
GO:0006913	Nucleocytoplasmic transport	20	6.56	2.54E-06	3.67	0.009313083	9.85E-05	9.26E-05
GO:0051169	Nuclear transport	20	6.56	2.65E-06	3.66	0.009705883	1.01E-04	9.45E-05

GO:0010639	Negative regulation of organelle organization	20	6.56	5.90E-05	2.93	0.195396942	0.001575361	0.001480429
GO:0050657	Nucleic acid transport	19	6.23	5.03E-10	6.72	1.85E-06	5.01E-08	4.71E-08
GO:0050658	RNA transport	19	6.23	5.03E-10	6.72	1.85E-06	5.01E-08	4.71E-08
GO:0051236	Establishment of RNA localization	19	6.23	6.45E-10	6.62	2.38E-06	6.25E-08	5.88E-08
GO:0006323	DNA packaging	19	6.23	7.12E-09	5.70	2.62E-05	5.14E-07	4.83E-07
GO:0015931	Nucleobase-containing compound transport	19	6.23	2.60E-08	5.25	9.58E-05	1.74E-06	1.64E-06
GO:0002181	Cytoplasmic translation	18	5.90	1.55E-10	7.81	5.70E-07	1.73E-08	1.62E-08
GO:0031497	Chromatin assembly	18	5.90	1.08E-09	6.90	3.99E-06	9.50E-08	8.92E-08
GO:0006333	Chromatin assembly or disassembly	18	5.90	6.81E-09	6.12	2.51E-05	5.02E-07	4.72E-07
GO:0006457	Protein folding	16	5.25	1.36E-06	4.80	0.005000359	5.97E-05	5.61E-05
GO:0006338	Chromatin remodeling	15	4.92	8.28E-06	4.42	0.030035626	2.70E-04	2.54E-04
GO:0071824	Protein-DNA complex subunit organization	15	4.92	1.65E-04	3.36	0.456277705	0.004089035	0.003842628
GO:0034655	Nucleobase-containing compound catabolic process	15	4.92	3.78E-04	3.10	0.75207259	0.008349433	0.007846291
GO:1903312	Negative regulation of mRNA metabolic process	14	4.59	7.56E-13	17.57	2.78E-09	1.33E-10	1.25E-10
GO:0043487	Regulation of RNA stability	14	4.59	8.15E-08	7.16	3.00E-04	4.88E-06	4.59E-06
GO:0051028	mRNA transport	14	4.59	1.36E-07	6.85	5.02E-04	7.50E-06	7.05E-06
GO:0051168	Nuclear export	14	4.59	7.87E-07	5.89	0.002894891	3.62E-05	3.41E-05
GO:0006401	RNA catabolic process	14	4.59	1.04E-05	4.67	0.037603447	3.28E-04	3.08E-04
GO:0043488	Regulation of mRNA stability	13	4.26	2.05E-07	7.35	7.55E-04	1.03E-05	9.72E-06
GO:0034728	Nucleosome organization	13	4.26	1.32E-05	4.96	0.047549401	4.03E-04	3.78E-04
GO:0065004	Protein-DNA complex assembly	13	4.26	4.47E-04	3.42	0.807719464	0.009639947	0.009059038
GO:0000245	Spliceosomal complex assembly	12	3.93	1.55E-07	8.54	5.69E-04	8.25E-06	7.75E-06
GO:0006611	Protein export from nucleus	12	3.93	5.69E-06	5.96	0.02073451	1.92E-04	1.80E-04
GO:0006402	mRNA catabolic process	12	3.93	2.87E-05	5.02	0.100281001	8.16E-04	7.67E-04
GO:0050821	Protein stabilization	12	3.93	3.71E-04	3.76	0.745150598	0.008250961	0.007753753
GO:2001252	Positive regulation of chromosome organization	12	3.93	5.79E-04	3.57	0.881783349	0.012128509	0.011397638
GO:0009913	Epidermal cell differentiation	12	3.93	7.68E-04	3.45	0.940974157	0.01562814	0.014686379
GO:0000381	Regulation of alternative mRNA splicing, via spliceosome	11	3.61	2.18E-08	12.05	8.03E-05	1.49E-06	1.40E-06
GO:0006405	RNA export from nucleus	11	3.61	3.15E-06	7.16	0.011539259	1.14E-04	1.07E-04
GO:0000956	Nuclear-transcribed mRNA catabolic process	11	3.61	2.53E-05	5.67	0.089047767	7.34E-04	6.90E-04

GO:0006334	Nucleosome assembly	11	3.61	2.88E-05	5.58	0.100584452	8.16E-04	7.67E-04
GO:0030216	Keratinocyte differentiation	11	3.61	1.26E-04	4.69	0.371887539	0.003184977	0.002993049
GO:0050686	Negative regulation of mRNA processing	10	3.28	6.82E-10	20.91	2.51E-06	6.44E-08	6.05E-08
GO:0033120	Positive regulation of RNA splicing	10	3.28	5.60E-09	16.83	2.06E-05	4.23E-07	3.98E-07
GO:1903313	Positive regulation of mRNA metabolic process	10	3.28	3.77E-07	10.62	0.001389616	1.83E-05	1.72E-05
GO:0006997	Nucleus organization	10	3.28	2.62E-04	4.76	0.619740912	0.00608035	0.005713944

* Plotted in Figure 4

The yellow highlight in the table means emphasize on the GO term "Cornified Envelope"
