# Supplementary material for: Thermostable Proteins from HaCaT Keratinocytes Identify a Wide Breadth of Intrinsically Disordered Proteins and Candidates for Liquid–Liquid Phase Separation
Source: Int J Mol Sci. 2022 Nov 18;23(22):14323. doi: 10.3390/ijms232214323 (PMC9692912; doi:10.3390/ijms232214323)
Supplement: Supplementary file 1 [file ijms-23-14323-s001.zip › Supplementary Table S3.pdf]

Supplementary Table S3. Investigation of keratinocyte protein intrinsic disorder in proteome database.

Entry	Protein names	RAPID % Disorder	SLIDER IDR propensity score (0-1)
P35321	Cornifin-A (19 kDa pancornulin) (SPRK) (Small proline-rich protein IA) (SPR-IA)	79.78	0.849
Q9UBC9	Small proline-rich protein 3 (22 kDa pancornulin) (Cornifin beta) (Esophagin)	78.7	0.615
P22528	Cornifin-B (14.9 kDa pancornulin) (Small proline-rich protein IB) (SPR-IB)	74.16	0.816
Q96PI1	Small proline-rich protein 4	73.42	0.877
O75475	PC4 and SFRS1-interacting protein (CLL-associated antigen KW-7) (Dense fine speckles 70 kDa protein) (DFS 70) (Lens epithelium-derived growth factor) (Transcriptional coactivator p75/p52)	68.3	0.956
P35325	Small proline-rich protein 2B (SPR-2B)	66.67	0.001
Q8TAE8	Growth arrest and DNA damage-inducible proteins-interacting protein 1 (39S ribosomal protein L59, mitochondrial) (MRP-L59) (CKII beta-associating protein) (CR6-interacting factor 1) (CRIF1) (Mitochondrial large ribosomal subunit protein mL64) (Papillomavirus L2-interacting nuclear protein 1) (PLINP) (PLINP-1) (p53-responsive gene 6 protein)	66.67	0.912
P07476	Involucrin	65.81	0.917
P35326	Small proline-rich protein 2A (SPR-2A) (2-1)	65.28	0.837
P23490	Loricrin	62.82	0.665
Q6F5E7	Protein TXNRD3NB (Thioredoxin reductase 2 intronic transcript 1) (Thioredoxin reductase 3 intronic transcript 1) (Thioredoxin reductase 3 neighbor gene protein) (TXNRD3 neighbor gene protein) (Thioredoxin reductase 3 new transcript 1)	62.41	0.742
P38159	RNA-binding motif protein, X chromosome (Glycoprotein p43) (Heterogeneous nuclear ribonucleoprotein G) (hnRNP G) [Cleaved into: RNA-binding motif protein, X chromosome, N-terminally processed]	62.4	0.878
Q14244	Ensconsin (Epithelial microtubule-associated protein of 115 kDa) (E-MAP-115) (Microtubule-associated protein 7) (MAP-7)	61.55	0.951
Q13247	Serine/arginine-rich splicing factor 6 (Pre-mRNA-splicing factor SRP55) (Splicing factor, arginine/serine-rich 6)	61.34	0.880
Q9H307	Pinin (140 kDa nuclear and cell adhesion-related phosphoprotein) (Desmosome-associated protein) (Domain-rich serine protein) (DRS protein) (DRSP) (Melanoma metastasis clone A protein) (Nuclear protein SDK3) (SR-like protein)	61.09	0.976
P22531	Small proline-rich protein 2E (SPR-2E) (Small proline-rich protein II) (SPR-II)	59.72	0.001
P22532	Small proline-rich protein 2D (SPR-2D) (Small proline-rich protein II) (SPR-II)	59.72	0.001
Q32MQ0	Zinc finger protein 750	58.64	0.881
Q96RM1	Small proline-rich protein 2F (SPR-2F)	58.33	0.001

Supplementary Table S3. Investigation of keratinocyte protein intrinsic disorder in proteome database.

Entry	Protein names	RAPID % Disorder	SLIDER IDR propensity score (0-1)
Q9BYE4	Small proline-rich protein 2G (SPR-2G)	57.53	0.001
Q9UHB6	LIM domain and actin-binding protein 1 (Epithelial protein lost in neoplasm)	57.44	0.892
P06753	Tropomyosin alpha-3 chain (Gamma-tropomyosin) (Tropomyosin-3) (Tropomyosin-5) (hTM5)	57.19	0.894
P20930	Filaggrin	55.58	0.939
Q8NEY8	Periphilin-1 (CDC7 expression repressor) (CR) (Gastric cancer antigen Ga50)	54.8	0.915
P20396	Pro-thyrotropin-releasing hormone (Pro-TRH) (Prothyroliberin) [Cleaved into: Thyrotropin-releasing hormone (TRH) (Protirelin) (TSH-releasing factor) (Thyroliberin) (Thyrotropin-releasing factor) (TRF)]	53.31	0.873
P67936	Tropomyosin alpha-4 chain (TM30p1) (Tropomyosin-4)	52.82	0.891
Q15517	Corneodesmosin (S protein)	52.36	0.807
P46937	Transcriptional coactivator YAP1 (Yes-associated protein 1) (Protein yorkie homolog) (Yes-associated protein YAP65 homolog)	51.59	0.892
Q14980	Nuclear mitotic apparatus protein 1 (Nuclear matrix protein-22) (NMP-22) (Nuclear mitotic apparatus protein) (NuMA protein) (SP-H antigen)	50.02	0.949
Q9UBG3	Cornulin (53 kDa putative calcium-binding protein) (53 kDa squamous epithelial-induced stress protein) (58 kDa heat shock protein) (Squamous epithelial heat shock protein 53) (Tumor-related protein)	49.49	0.845
Q5D862	Filaggrin-2 (FLG-2) (Intermediate filament-associated and psoriasis-susceptibility protein) (Ifapsoriasin)	49.14	0.846
Q07325	C-X-C motif chemokine 9 (Gamma-interferon-induced monokine) (Monokine induced by interferon-gamma) (HuMIG) (MIG) (Small-inducible cytokine B9)	47.2	0.723
P26651	mRNA decay activator protein ZFP36 (G0/G1 switch regulatory protein 24) (Growth factor-inducible nuclear protein NUP475) (Tristetraprolin) (Zinc finger protein 36) (Zfp-36)	46.63	0.718
Q13886	Krueppel-like factor 9 (Basic transcription element-binding protein 1) (BTE-binding protein 1) (GC-box-binding protein 1) (Transcription factor BTEB1)	46.31	0.795
P31151	Protein S100-A7 (Psoriasin) (S100 calcium-binding protein A7)	45.54	0.528
Q14157	Ubiquitin-associated protein 2-like (Protein NICE-4)	45.17	0.888
P0C864	Putative uncharacterized protein DANCR (Anti-differentiation ncRNA protein) (Differentiation antagonizing non-protein coding RNA) (Small nucleolar RNA host gene protein 13)	44.79	0.675
P10914	Interferon regulatory factor 1 (IRF-1)	44.62	0.803
Q9HCS4	Transcription factor 7-like 1 (HMG box transcription factor 3) (TCF-3)	44.56	0.931
O94885	SAM and SH3 domain-containing protein 1 (Proline-glutamate repeat-containing protein)	44.19	0.947

Supplementary Table S3. Investigation of keratinocyte protein intrinsic disorder in proteome database.

Entry	Protein names	RAPID % Disorder	SLIDER IDR propensity score (0-1)
Q8NEV8	Exophilin-5 (Synaptotagmin-like protein homolog lacking C2 domains b) (SlaC2-b) (Slp homolog lacking C2 domains b)	43.89	0.901
Q9NX09	DNA damage-inducible transcript 4 protein (HIF-1 responsive protein RTP801) (Protein regulated in development and DNA damage response 1) (REDD-1)	43.53	0.677
Q6UXA7	Uncharacterized protein C6orf15 (Protein STG)	42.15	0.724
O14974	Protein phosphatase 1 regulatory subunit 12A (Myosin phosphatase-targeting subunit 1) (Myosin phosphatase target subunit 1) (Protein phosphatase myosin-binding subunit)	42.04	0.946
P15408	Fos-related antigen 2 (FRA-2)	42.02	0.883
Q96F24	Nuclear receptor-binding factor 2 (NRBF-2) (Comodulator of PPAR and RXR)	41.81	0.773
Q06323	Proteasome activator complex subunit 1 (11S regulator complex subunit alpha) (REG-alpha) (Activator of multicatalytic protease subunit 1) (Interferon gamma up-regulated I-5111 protein) (IGUP I-5111) (Proteasome activator 28 subunit alpha) (PA28a) (PA28alpha)	41.77	0.714
O95999	B-cell lymphoma/leukemia 10 (B-cell CLL/lymphoma 10) (Bcl-10) (CARD-containing molecule enhancing NF-kappa-B) (CARD-like apoptotic protein) (hCLAP) (CED-3/ICH-1 prodomain homologous E10-like regulator) (CIPER) (Cellular homolog of vCARMEN) (cCARMEN) (Cellular-E10) (c-E10) (Mammalian CARD-containing adapter molecule E10) (mE10)	41.63	0.775
O95171	Sciellin	41.57	0.861
Q9UKI9	POU domain, class 2, transcription factor 3 (Octamer-binding protein 11) (Oct-11) (Octamer-binding transcription factor 11) (OTF-11) (Transcription factor PLA-1) (Transcription factor Skn-1)	41.51	0.821
Q8N131	Porimin (Keratinocytes-associated transmembrane protein 3) (KCT-3) (Pro-oncosis receptor inducing membrane injury) (Transmembrane protein 123)	41.35	0.741
Q8NAX2	Keratinocyte differentiation factor 1	41.21	0.815
Q6UWP8	Suprabasin	41.02	0.493
Q9UMD9	Collagen alpha-1(XVII) chain (180 kDa bullous pemphigoid antigen 2) (Bullous pemphigoid antigen 2) [Cleaved into: 120 kDa linear IgA disease antigen (120 kDa linear IgA dermatosis antigen) (Linear IgA disease antigen 1) (LAD-1); 97 kDa linear IgA disease antigen (97 kDa linear IgA bullous dermatosis antigen) (97 kDa LAD antigen) (97-LAD) (Linear IgA bullous disease antigen of 97 kDa) (LABD97)]	40.75	0.913
Q9BTP6	Zinc finger BED domain-containing protein 2	39.91	0.816
P04637	Cellular tumor antigen p53 (Antigen NY-CO-13) (Phosphoprotein p53) (Tumor suppressor p53)	39.69	0.866
Q9NQ38	Serine protease inhibitor Kazal-type 5 (Lympho-epithelial Kazal-type-related inhibitor) (LEKTI) [Cleaved into: Hemofiltrate peptide HF6478; Hemofiltrate peptide HF7665]	39.47	0.897

Supplementary Table S3. Investigation of keratinocyte protein intrinsic disorder in proteome database.

Entry	Protein names	RAPID % Disorder	SLIDER IDR propensity score (0-1)
Q15648	Mediator of RNA polymerase II transcription subunit 1 (Activator-recruited cofactor 205 kDa component) (ARC205) (Mediator complex subunit 1) (Peroxisome proliferator-activated receptor-binding protein) (PBP) (PPAR-binding protein) (Thyroid hormone receptor-associated protein complex 220 kDa component) (Trap220) (Thyroid receptor-interacting protein 2) (TR-interacting protein 2) (TRIP-2) (Vitamin D receptor-interacting protein complex component DRIP205) (p53 regulatory protein RB18A)	39.47	0.931
Q9P0M6	Core histone macro-H2A.2 (Histone macroH2A2) (mH2A2)	39.25	0.807
P35527	Keratin, type I cytoskeletal 9 (Cytokeratin-9) (CK-9) (Keratin-9) (K9)	38.84	0.793
Q03052	POU domain, class 3, transcription factor 1 (Octamer-binding protein 6) (Oct-6) (Octamer-binding transcription factor 6) (OTF-6) (POU domain transcription factor SCIP)	38.8	0.773
Q674X7	Kazrin	38.71	0.877
P41271	Neuroblastoma suppressor of tumorigenicity 1 (DAN domain family member 1) (Protein N03) (Zinc finger protein DAN)	38.67	0.528
Q6E0U4	Dermokine (Epidermis-specific secreted protein SK30/SK89)	38.66	0.755
Q96KS9	Protein FAM167A	38.32	0.817
Q9H201	Epsin-3 (EPS-15-interacting protein 3)	38.29	0.865
P27824	Calnexin (IP90) (Major histocompatibility complex class I antigen-binding protein p88) (p90)	38.01	0.884
P10070	Zinc finger protein GLI2 (GLI family zinc finger protein 2) (Tax helper protein)	37.26	0.904
Q14152	Eukaryotic translation initiation factor 3 subunit A (eIF3a) (Eukaryotic translation initiation factor 3 subunit 10) (eIF-3-theta) (eIF3 p167) (eIF3 p180) (eIF3 p185)	37.05	0.946
P31948	Stress-induced-phosphoprotein 1 (STI1) (Hsc70/Hsp90-organizing protein) (Hop) (Renal carcinoma antigen NY-REN-11) (Transformation-sensitive protein IEF SSP 3521)	37.02	0.856
Q02388	Collagen alpha-1(VII) chain (Long-chain collagen) (LC collagen)	37.02	0.874
Q9Y6Q9	Nuclear receptor coactivator 3 (NCoA-3) (EC 2.3.1.48) (ACTR) (Amplified in breast cancer 1 protein) (AIB-1) (CBP-interacting protein) (pCIP) (Class E basic helix-loop-helix protein 42) (bHLHe42) (Receptor-associated coactivator 3) (RAC-3) (Steroid receptor coactivator protein 3) (SRC-3) (Thyroid hormone receptor activator molecule 1) (TRAM-1)	36.94	0.921
Q9BXF6	Rab11 family-interacting protein 5 (Rab11-FIP5) (Gamma-SNAP-associated factor 1) (Gaf-1) (Phosphoprotein pp75) (Rab11-interacting protein Rip11)	36.6	0.867
O75367	Core histone macro-H2A.1 (Histone macroH2A1) (mH2A1) (Histone H2A.y) (H2A/y) (Medulloblastoma antigen MU-MB-50.205)	36.29	0.713
O60870	DNA/RNA-binding protein KIN17 (Binding to curved DNA) (KIN, antigenic determinant of recA protein homolog)	36.13	0.807

Supplementary Table S3. Investigation of keratinocyte protein intrinsic disorder in proteome database.

Entry	Protein names	RAPID % Disorder	SLIDER IDR propensity score (0-1)
Q8WX93	Palladin (SIH002) (Sarcoma antigen NY-SAR-77)	35.21	0.888
Q13951	Core-binding factor subunit beta (CBF-beta) (Polyomavirus enhancer-binding protein 2 beta subunit) (PEA2-beta) (PEBP2-beta) (SL3-3 enhancer factor 1 subunit beta) (SL3/AKV core-binding factor beta subunit)	35.16	0.675
Q92838	Ectodysplasin-A (Ectodermal dysplasia protein) (EDA protein) [Cleaved into: Ectodysplasin-A, membrane form; Ectodysplasin-A, secreted form]	35.04	0.819
O15353	Forkhead box protein N1 (Winged-helix transcription factor nude)	35.03	0.796
P27797	Calreticulin (CRP55) (Calregulin) (Endoplasmic reticulum resident protein 60) (ERp60) (HACBP) (grp60)	35.01	0.863
Q8TD31	Coiled-coil alpha-helical rod protein 1 (Alpha-helical coiled-coil rod protein) (Putative gene 8 protein) (Pg8)	34.91	0.919
P55089	Urocortin	34.68	0.838
Q07352	mRNA decay activator protein ZFP36L1 (Butyrate response factor 1) (EGF-response factor 1) (ERF-1) (TPA-induced sequence 11b) (Zinc finger protein 36, C3H1 type-like 1) (ZFP36-like 1)	34.62	0.728
Q86YL7	Podoplanin (Aggrus) (Glycoprotein 36) (Gp36) (PA2.26 antigen) (T1-alpha) (T1A) [Cleaved into: 29kDa cytosolic podoplanin intracellular domain (PICD)]	33.95	0.541
Q15555	Microtubule-associated protein RP/EB family member 2 (APC-binding protein EB2) (End-binding protein 2) (EB2)	33.94	0.817
Q9UJM3	ERBB receptor feedback inhibitor 1 (Mitogen-inducible gene 6 protein) (MIG-6)	33.77	0.808
P28698	Myeloid zinc finger 1 (MZF-1) (Zinc finger and SCAN domain-containing protein 6) (Zinc finger protein 42)	33.65	0.784
O95835	Serine/threonine-protein kinase LATS1 (EC 2.7.11.1) (Large tumor suppressor homolog 1) (WARTS protein kinase) (h-warts)	33.63	0.907
P30047	GTP cyclohydrolase 1 feedback regulatory protein (GFRP) (GTP cyclohydrolase I feedback regulatory protein) (p35)	33.33	0.303
Q4ZHG4	Fibronectin type III domain-containing protein 1 (Activation-associated cDNA protein) (Expressed in synovial lining protein)	32.95	0.930
Q969S3	Zinc finger protein 622 (Zinc finger-like protein 9)	32.91	0.867
P21741	Midkine (MK) (Amphiregulin-associated protein) (ARAP) (Midgestation and kidney protein) (Neurite outgrowth-promoting factor 2) (Neurite outgrowth-promoting protein)	32.87	0.538
P15692	Vascular endothelial growth factor A (VEGF-A) (Vascular permeability factor) (VPF)	32.76	0.720
Q9UN37	Vacuolar protein sorting-associated protein 4A (EC 3.6.4.6) (Protein SKD2) (VPS4-1) (hVPS4)	32.72	0.768
O14543	Suppressor of cytokine signaling 3 (SOCS-3) (Cytokine-inducible SH2 protein 3) (CIS-3) (STAT-induced STAT inhibitor 3) (SSI-3)	32.44	0.632
P31947	14-3-3 protein sigma (Epithelial cell marker protein 1) (Stratifin)	32.26	0.754

Supplementary Table S3. Investigation of keratinocyte protein intrinsic disorder in proteome database.

Entry	Protein names	RAPID % Disorder	SLIDER IDR propensity score (0-1)
Q13043	Serine/threonine-protein kinase 4 (EC 2.7.11.1) (Mammalian STE20-like protein kinase 1) (MST-1) (STE20-like kinase MST1) (Serine/threonine-protein kinase Krs-2) [Cleaved into: Serine/threonine-protein kinase 4 37kDa subunit (MST1/N); Serine/threonine-protein kinase 4 18kDa subunit (MST1/C)]	31.62	0.805
P24534	Elongation factor 1-beta (EF-1-beta)	31.56	0.728
Q9NXR8	Inhibitor of growth protein 3 (p47ING3)	31.1	0.904
Q12948	Forkhead box protein C1 (Forkhead-related protein FKHL7) (Forkhead-related transcription factor 3) (FREAC-3)	31.1	0.863
Q13464	Rho-associated protein kinase 1 (EC 2.7.11.1) (Renal carcinoma antigen NY-REN-35) (Rho-associated, coiled-coil-containing protein kinase 1) (Rho-associated, coiled-coil-containing protein kinase I) (ROCK-I) (p160 ROCK-1) (p160ROCK)	31.09	0.883
Q9Y448	Small kinetochore-associated protein (SKAP) (Kinetochore-localized astrin-binding protein) (Kinastrin) (Kinetochore-localized astrin/SPAG5-binding protein) (TRAF4-associated factor 1)	31.01	0.836
Q96LB3	Intraflagellar transport protein 74 homolog (Capillary morphogenesis gene 1 protein) (CMG-1) (Coiled-coil domain-containing protein 2)	31	0.856
P15514	Amphiregulin (AR) (Colorectum cell-derived growth factor) (CRDGF)	30.95	0.819
P19957	Elafin (Elastase-specific inhibitor) (ESI) (Peptidase inhibitor 3) (PI-3) (Protease inhibitor WAP3) (Skin-derived antileukoproteinase) (SKALP) (WAP four-disulfide core domain protein 14)	30.77	0.308
P13645	Keratin, type I cytoskeletal 10 (Cytokeratin-10) (CK-10) (Keratin-10) (K10)	30.48	0.754
O60437	Periplakin (190 kDa paraneoplastic pemphigus antigen) (195 kDa cornified envelope precursor protein)	30.3	0.904
O14896	Interferon regulatory factor 6 (IRF-6)	30.19	0.701
Q5D1E8	Endoribonuclease ZC3H12A (EC 3.1.-.-) (Monocyte chemotactic protein-induced protein 1) (MCP-induced protein 1) (MCPIP-1) (Regnase-1) (Reg1) (Zinc finger CCCH domain-containing protein 12A)	30.05	0.799
O60716	Catenin delta-1 (Cadherin-associated Src substrate) (CAS) (p120 catenin) (p120(ctn)) (p120(cas))	29.44	0.803
Q01196	Runt-related transcription factor 1 (Acute myeloid leukemia 1 protein) (Core-binding factor subunit alpha-2) (CBF-alpha-2) (Oncogene AML-1) (Polyomavirus enhancer-binding protein 2 alpha B subunit) (PEA2-alpha B) (PEBP2-alpha B) (SL3-3 enhancer factor 1 alpha B subunit) (SL3/AKV core-binding factor alpha B subunit)	29.14	0.786
Q5T749	Keratinocyte proline-rich protein (hKPRP)	29.02	0.823
O43734	E3 ubiquitin ligase TRAF3IP2 (EC 2.3.2.27) (Adapter protein CIKS) (Connection to IKK and SAPK/JNK) (E3 ubiquitin-protein ligase CIKS) (Nuclear factor NF-kappa-B activator 1) (ACT1) (TRAF3-interacting protein 2)	28.92	0.831
P47974	mRNA decay activator protein ZFP36L2 (Butyrate response factor 2) (EGF-response factor 2) (ERF-2) (TPA-induced sequence 11d) (Zinc finger protein 36, C3H1 type-like 2) (ZFP36-like 2)	28.74	0.841

Supplementary Table S3. Investigation of keratinocyte protein intrinsic disorder in proteome database.

Entry	Protein names	RAPID % Disorder	SLIDER IDR propensity score (0-1)
Q9H4B6	Protein salvador homolog 1 (45 kDa WW domain protein) (hWW45)	28.72	0.742
Q2M1P5	Kinesin-like protein KIF7	28.67	0.889
Q9P0W0	Interferon kappa (IFN-kappa)	28.5	0.597
Q9UK53	Inhibitor of growth protein 1	28.44	0.842
Q09472	Histone acetyltransferase p300 (p300 HAT) (EC 2.3.1.48) (E1A-associated protein p300) (Histone butyryltransferase p300) (EC 2.3.1.-) (Histone crotonyltransferase p300) (EC 2.3.1.-) (Protein 2-hydroxyisobutyryltransferase p300) (EC 2.3.1.-) (Protein lactyltransferas p300) (EC 2.3.1.-) (Protein propionyltransferase p300) (EC 2.3.1.-)	28.38	0.941
P43268	ETS translocation variant 4 (Adenovirus E1A enhancer-binding protein) (E1A-F) (Polyomavirus enhancer activator 3 homolog) (Protein PEA3)	28.31	0.595
Q96RE7	Nucleus accumbens-associated protein 1 (NAC-1) (BTB/POZ domain-containing protein 14B)	28.08	0.826
P06702	Protein S100-A9 (Calgranulin-B) (Calprotectin L1H subunit) (Leukocyte L1 complex heavy chain) (Migration inhibitory factor-related protein 14) (MRP-14) (p14) (S100 calcium-binding protein A9)	28.07	0.505
P31268	Homeobox protein Hox-A7 (Homeobox protein Hox 1.1) (Homeobox protein Hox-1A)	27.83	0.778
Q14512	Fibroblast growth factor-binding protein 1 (FGF-BP) (FGF-BP1) (FGF-binding protein 1) (FGFBP-1) (17 kDa heparin-binding growth factor-binding protein) (17 kDa HBGF-binding protein) (HBp17)	27.78	0.674
P01040	Cystatin-A (Cystatin-AS) (Stefin-A) [Cleaved into: Cystatin-A, N-terminally processed]	27.55	0.374
Q96AV8	Transcription factor E2F7 (E2F-7)	27.55	0.873
Q8IZP1	TBC1 domain family member 3 (Prostate cancer gene 17 protein) (Protein TRE17-alpha) (Rab GTPase-activating protein PRC17)	27.5	0.748
Q9BRP0	Transcription factor Ovo-like 2 (hOvo2) (Zinc finger protein 339)	26.91	0.764
O43852	Calumenin (Crocalbin) (IEF SSP 9302)	26.67	0.517
Q92817	Envoplakin (210 kDa cornified envelope precursor protein) (210 kDa paraneoplastic pemphigus antigen) (p210)	26.41	0.916
O75116	Rho-associated protein kinase 2 (EC 2.7.11.1) (Rho kinase 2) (Rho-associated, coiled-coil-containing protein kinase 2) (Rho-associated, coiled-coil-containing protein kinase II) (ROCK-II) (p164 ROCK-2)	26.22	0.895
Q6FHJ7	Secreted frizzled-related protein 4 (sFRP-4) (Frizzled protein, human endometrium) (FrpHE)	26.01	0.757
Q96MM3	Zinc finger protein 42 homolog (Zfp-42) (Reduced expression protein 1) (REX-1) (hREX-1) (Zinc finger protein 754)	25.81	0.717
Q8NC54	Keratinocyte-associated transmembrane protein 2	25.66	0.773

Supplementary Table S3. Investigation of keratinocyte protein intrinsic disorder in proteome database.

Entry	Protein names	RAPID % Disorder	SLIDER IDR propensity score (0-1)
Q7Z7E8	Ubiquitin-conjugating enzyme E2 Q1 (EC 2.3.2.23) (E2 ubiquitin-conjugating enzyme Q1) (Protein NICE-5) (Ubiquitin carrier protein Q1) (Ubiquitin-protein ligase Q1)	25.59	0.799
P49662	Caspase-4 (CASP-4) (EC 3.4.22.57) (ICE and Ced-3 homolog 2) (ICH-2) (ICE(rel)-II) (Mih1) (Protease TX) [Cleaved into: Caspase-4 subunit p10; Caspase-4 subunit p20]	25.46	0.642
O15055	Period circadian protein homolog 2 (hPER2) (Circadian clock protein PERIOD 2)	25.34	0.917
Q6IBS0	Twinfilin-2 (A6-related protein) (hA6RP) (Protein tyrosine kinase 9-like) (Twinfilin-1-like protein)	25.21	0.521
O95819	Mitogen-activated protein kinase kinase kinase kinase 4 (EC 2.7.11.1) (HPK/GCK-like kinase HGK) (MAPK/ERK kinase kinase kinase 4) (MEK kinase kinase 4) (MEKKK 4) (Nck-interacting kinase)	25.02	0.904
P06400	Retinoblastoma-associated protein (p105-Rb) (p110-RB1) (pRb) (Rb) (pp110)	25	0.856
P18031	Tyrosine-protein phosphatase non-receptor type 1 (EC 3.1.3.48) (Protein-tyrosine phosphatase 1B) (PTP-1B)	24.6	0.726
P02778	C-X-C motif chemokine 10 (10 kDa interferon gamma-induced protein) (Gamma-IP10) (IP-10) (Small-inducible cytokine B10) [Cleaved into: CXCL10(1-73)]	24.49	0.551
Q6DN90	IQ motif and SEC7 domain-containing protein 1 (ADP-ribosylation factors guanine nucleotide-exchange protein 100) (ADP-ribosylation factors guanine nucleotide-exchange protein 2) (Brefeldin-resistant Arf-GEF 2 protein) (BRAG2)	24.4	0.904
Q9H329	Band 4.1-like protein 4B (Erythrocyte membrane protein band 4.1-like 4B) (FERM-containing protein CG1) (Protein EHM2)	24.22	0.853
Q8N6P7	Interleukin-22 receptor subunit alpha-1 (IL-22 receptor subunit alpha-1) (IL-22R-alpha-1) (IL-22RA1) (Cytokine receptor class-II member 9) (Cytokine receptor family 2 member 9) (CRF2-9) (ZcytoR11)	24.22	0.595
O96020	G1/S-specific cyclin-E2	24.01	0.610
Q14118	Dystroglycan (Dystrophin-associated glycoprotein 1) [Cleaved into: Alpha-dystroglycan (Alpha-DG); Beta-dystroglycan (Beta-DG)]	23.46	0.810
Q6VUC0	Transcription factor AP-2-epsilon (AP2-epsilon) (Activating enhancer-binding protein 2-epsilon)	23.3	0.712
P60985	Keratinocyte differentiation-associated protein	23.23	0.463
O43623	Zinc finger protein SNAI2 (Neural crest transcription factor Slug) (Protein snail homolog 2)	23.13	0.666
Q6ZN30	Zinc finger protein basonuclin-2	23.02	0.897
Q6F5E8	Capping protein, Arp2/3 and myosin-I linker protein 2 (Capping protein regulator and myosin 1 linker 2) (F-actin-uncapping protein RLTPR) (Leucine-rich repeat-containing protein 16C) (RGD, leucine-rich repeat, tropomodulin and proline-rich-containing protein)	22.65	0.906
A1YPR0	Zinc finger and BTB domain-containing protein 7C (Affected by papillomavirus DNA integration in ME180 cells protein 1) (APM-1) (Zinc finger and BTB domain-containing protein 36) (Zinc finger protein 857C)	22.62	0.907

Supplementary Table S3. Investigation of keratinocyte protein intrinsic disorder in proteome database.

Entry	Protein names	RAPID % Disorder	SLIDER IDR propensity score (0-1)
P11274	Breakpoint cluster region protein (EC 2.7.11.1) (Renal carcinoma antigen NY-REN-26)	22.42	0.887
Q13610	Periodic tryptophan protein 1 homolog (Keratinocyte protein IEF SSP 9502)	22.36	0.757
Q8N2W9	E3 SUMO-protein ligase PIAS4 (EC 2.3.2.27) (PIASy) (Protein inhibitor of activated STAT protein 4) (Protein inhibitor of activated STAT protein gamma) (PIAS-gamma) (RING-type E3 ubiquitin transferase PIAS4)	22.35	0.803
Q01954	Zinc finger protein basonuclin-1	22.33	0.850
P27348	14-3-3 protein theta (14-3-3 protein T-cell) (14-3-3 protein tau) (Protein HS1)	22.04	0.670
Q9NZI5	Grainyhead-like protein 1 homolog (Mammalian grainyhead) (NH32) (Transcription factor CP2-like 2) (Transcription factor LBP-32)	21.84	0.662
Q05469	Hormone-sensitive lipase (HSL) (EC 3.1.1.79) (Monoacylglycerol lipase LIPE) (EC 3.1.1.23) (Retinyl ester hydrolase) (REH)	21.75	0.849
O00358	Forkhead box protein E1 (Forkhead box protein E2) (Forkhead-related protein FKHL15) (HFKH4) (HNF-3/fork head-like protein 5) (HFKL5) (Thyroid transcription factor 2) (TTF-2)	21.72	0.765
P10321	HLA class I histocompatibility antigen, C alpha chain (HLA-C) (HLA-Cw) (Human leukocyte antigen C)	21.58	0.729
Q96TC7	Regulator of microtubule dynamics protein 3 (RMD-3) (hRMD-3) (Cerebral protein 10) (Protein FAM82A2) (Protein FAM82C) (Protein tyrosine phosphatase-interacting protein 51) (TCPTP-interacting protein 51)	21.49	0.799
Q03001	Dystonin (230 kDa bullous pemphigoid antigen) (230/240 kDa bullous pemphigoid antigen) (Bullous pemphigoid antigen 1) (BPA) (Bullous pemphigoid antigen) (Dystonia musculorum protein) (Hemidesmosomal plaque protein)	21.4	0.928
P31946	14-3-3 protein beta/alpha (Protein 1054) (Protein kinase C inhibitor protein 1) (KCIP-1) [Cleaved into: 14-3-3 protein beta/alpha, N-terminally processed]	21.14	0.664
P35908	Keratin, type II cytoskeletal 2 epidermal (Cytokeratin-2e) (CK-2e) (Epithelial keratin-2e) (Keratin-2 epidermis) (Keratin-2e) (K2e) (Type-II keratin Kb2)	21.13	0.776
Q13835	Plakophilin-1 (Band 6 protein) (B6P)	21.02	0.714
P13693	Translationally-controlled tumor protein (TCTP) (Fortilin) (Histamine-releasing factor) (HRF) (p23)	20.93	0.474
P11229	Muscarinic acetylcholine receptor M1	20.87	0.760
Q15828	Cystatin-M (Cystatin-6) (Cystatin-E)	20.81	0.568
Q969D9	Thymic stromal lymphopoietin	20.75	0.381
Q9H3D4	Tumor protein 63 (p63) (Chronic ulcerative stomatitis protein) (CUSP) (Keratinocyte transcription factor KET) (Transformation-related protein 63) (TP63) (Tumor protein p73-like) (p73L) (p40) (p51)	20.44	0.794
Q08209	Serine/threonine-protein phosphatase 2B catalytic subunit alpha isoform (EC 3.1.3.16) (CAM-PRP catalytic subunit) (Calmodulin-dependent calcineurin A subunit alpha isoform)	20.35	0.656

Supplementary Table S3. Investigation of keratinocyte protein intrinsic disorder in proteome database.

Entry	Protein names	RAPID % Disorder	SLIDER IDR propensity score (0-1)
O95361	Tripartite motif-containing protein 16 (EC 2.3.2.27) (E3 ubiquitin-protein ligase TRIM16) (Estrogen-responsive B box protein)	20.21	0.782
Q9UGL9	Cysteine-rich C-terminal protein 1 (Protein NICE-1)	20.2	0.880
Q14188	Transcription factor Dp-2 (E2F dimerization partner 2)	20.18	0.796
A0A2R8Y7D0	Ubiquitin domain-containing protein TINCR (Placenta-specific protein 2) (Terminal differentiation-induced cornification regulator)	20	0.508
Q9H1E1	Ribonuclease 7 (RNase 7) (EC 3.1.27.-) (Skin-derived antimicrobial protein 2) (SAP-2)	19.87	0.594
P14866	Heterogeneous nuclear ribonucleoprotein L (hnRNP L)	19.86	0.784
P60484	Phosphatidylinositol 3,4,5-trisphosphate 3-phosphatase and dual-specificity protein phosphatase PTEN (EC 3.1.3.16) (EC 3.1.3.48) (EC 3.1.3.67) (Mutated in multiple advanced cancers 1) (Phosphatase and tensin homolog)	19.85	0.655
Q8N8W4	Omega-hydroxyceramide transacylase (EC 2.3.1.296) (Patatin-like phospholipase domain-containing protein 1)	19.74	0.783
Q9BXL6	Caspase recruitment domain-containing protein 14 (CARD-containing MAGUK protein 2) (Carma 2)	19.72	0.814
Q9BYX2	TBC1 domain family member 2A (Armus) (Prostate antigen recognized and identified by SEREX 1) (PARIS-1)	19.61	0.853
Q02750	Dual specificity mitogen-activated protein kinase kinase 1 (MAP kinase kinase 1) (MAPKK 1) (MKK1) (EC 2.7.12.2) (ERK activator kinase 1) (MAPK/ERK kinase 1) (MEK 1)	19.59	0.648
Q9H4L7	SWI/SNF-related matrix-associated actin-dependent regulator of chromatin subfamily A containing DEAD/H box 1 (EC 3.6.4.12) (ATP-dependent helicase 1) (hHEL1)	19.49	0.862
Q2M2I5	Keratin, type I cytoskeletal 24 (Cytokeratin-24) (CK-24) (Keratin-24) (K24) (Type I keratin-24)	19.43	0.802
Q6ISB3	Grainyhead-like protein 2 homolog (Brother of mammalian grainyhead) (Transcription factor CP2-like 3)	19.36	0.689
P21583	Kit ligand (Mast cell growth factor) (MGF) (Stem cell factor) (SCF) (c-Kit ligand) [Cleaved into: Soluble KIT ligand (sKITLG)]	19.05	0.644
P31944	Caspase-14 (CASP-14) (EC 3.4.22.-) [Cleaved into: Caspase-14 subunit p17, mature form; Caspase-14 subunit p10, mature form; Caspase-14 subunit p20, intermediate form; Caspase-14 subunit p8, intermediate form]	19.01	0.636
Q8TB72	Pumilio homolog 2 (Pumilio-2)	18.95	0.867
O14936	Peripheral plasma membrane protein CASK (hCASK) (EC 2.7.11.1) (Calcium/calmodulin-dependent serine protein kinase) (Protein lin-2 homolog)	18.9	0.710
Q99755	Phosphatidylinositol 4-phosphate 5-kinase type-1 alpha (PIP5K1-alpha) (PtdIns(4)P-5-kinase 1 alpha) (EC 2.7.1.68) (68 kDa type I phosphatidylinositol 4-phosphate 5-kinase alpha) (Phosphatidylinositol 4-phosphate 5-kinase type I alpha) (PIP5KIalpha)	18.86	0.688
O14763	Tumor necrosis factor receptor superfamily member 10B (Death receptor 5) (TNF-related apoptosis-inducing ligand receptor 2) (TRAIL receptor 2) (TRAIL-R2) (CD antigen CD262)	18.86	0.795

Supplementary Table S3. Investigation of keratinocyte protein intrinsic disorder in proteome database.

Entry	Protein names	RAPID % Disorder	SLIDER IDR propensity score (0-1)
P42772	Cyclin-dependent kinase 4 inhibitor B (Multiple tumor suppressor 2) (MTS-2) (p14-INK4b) (p15-INK4b) (p15INK4B)	18.84	0.436
Q9BX95	Sphingosine-1-phosphate phosphatase 1 (SPPase1) (Spp1) (hSPP1) (hSPPase1) (EC 3.1.3.-) (Sphingosine-1-phosphatase 1) (Sphingosine-1-phosphate phosphohydrolase 1) (SPP-1)	18.82	0.466
P21802	Fibroblast growth factor receptor 2 (FGFR-2) (EC 2.7.10.1) (K-sam) (KGFR) (Keratinocyte growth factor receptor) (CD antigen CD332)	18.76	0.695
Q9BYD5	Cornifelin	18.75	0.173
P61586	Transforming protein RhoA (EC 3.6.5.2) (Rho cDNA clone 12) (h12)	18.65	0.517
Q01546	Keratin, type II cytoskeletal 2 oral (Cytokeratin-2P) (CK-2P) (K2P) (Keratin-76) (K76) (Type-II keratin Kb9)	18.5	0.785
P47929	Galectin-7 (Gal-7) (HKL-14) (PI7) (p53-induced gene 1 protein)	18.38	0.277
Q99075	Proheparin-binding EGF-like growth factor [Cleaved into: Heparin-binding EGF-like growth factor (HB-EGF) (HBEGF) (Diphtheria toxin receptor) (DT-R)]	18.27	0.644
Q03181	Peroxisome proliferator-activated receptor delta (PPAR-delta) (NUCI) (Nuclear hormone receptor 1) (NUC1) (Nuclear receptor subfamily 1 group C member 2) (Peroxisome proliferator-activated receptor beta) (PPAR-beta)	18.14	0.746
Q6PD74	Alpha- and gamma-adaptin-binding protein p34	18.1	0.616
P01579	Interferon gamma (IFN-gamma) (Immune interferon)	18.07	0.454
P23284	Peptidyl-prolyl cis-trans isomerase B (PPIase B) (EC 5.2.1.8) (CYP-S1) (Cyclophilin B) (Rotamase B) (S-cyclophilin) (SCYLP)	18.06	0.356
Q16513	Serine/threonine-protein kinase N2 (EC 2.7.11.13) (PKN gamma) (Protein kinase C-like 2) (Protein-kinase C-related kinase 2)	17.99	0.810
P22223	Cadherin-3 (Placental cadherin) (P-cadherin)	17.85	0.706
P11473	Vitamin D3 receptor (VDR) (1,25-dihydroxyvitamin D3 receptor) (Nuclear receptor subfamily 1 group I member 1)	17.8	0.804
P32456	Guanylate-binding protein 2 (EC 3.6.5.-) (GTP-binding protein 2) (GBP-2) (HuGBP-2) (Guanine nucleotide-binding protein 2) (Interferon-induced guanylate-binding protein 2)	17.77	0.759
Q96FQ6	Protein S100-A16 (Aging-associated gene 13 protein) (Protein S100-F) (S100 calcium-binding protein A16)	17.48	0.369
Q04917	14-3-3 protein eta (Protein AS1)	17.48	0.608
Q12923	Tyrosine-protein phosphatase non-receptor type 13 (EC 3.1.3.48) (Fas-associated protein-tyrosine phosphatase 1) (FAP-1) (PTP-BAS) (Protein-tyrosine phosphatase 1E) (PTP-E1) (hPTPE1) (Protein-tyrosine phosphatase PTPL1)	17.42	0.870
Q99715	Collagen alpha-1(XII) chain	17.34	0.799

Supplementary Table S3. Investigation of keratinocyte protein intrinsic disorder in proteome database.

Entry	Protein names	RAPID % Disorder	SLIDER IDR propensity score (0-1)
Q9P0M4	Interleukin-17C (IL-17C) (Cytokine CX2)	17.26	0.516
Q96JQ0	Protocadherin-16 (Cadherin-19) (Cadherin-25) (Fibroblast cadherin-1) (Protein dachsous homolog 1)	17.19	0.803
P31949	Protein S100-A11 (Calgizzarin) (Metastatic lymph node gene 70 protein) (MLN 70) (Protein S100-C) (S100 calcium-binding protein A11) [Cleaved into: Protein S100-A11, N-terminally processed]	17.14	0.340
Q96FX8	p53 apoptosis effector related to PMP-22 (Keratinocyte-associated protein 1) (KCP-1) (P53-induced protein PIGPC1) (Transmembrane protein THW)	17.1	0.127
Q8WXH5	Suppressor of cytokine signaling 4 (SOCS-4) (Suppressor of cytokine signaling 7) (SOCS-7)	17.05	0.696
P30101	Protein disulfide-isomerase A3 (EC 5.3.4.1) (58 kDa glucose-regulated protein) (58 kDa microsomal protein) (p58) (Disulfide isomerase ER-60) (Endoplasmic reticulum resident protein 57) (ER protein 57) (ERp57) (Endoplasmic reticulum resident protein 60) (ER protein 60) (ERp60)	17.03	0.629
Q92831	Histone acetyltransferase KAT2B (EC 2.3.1.48) (Histone acetyltransferase PCAF) (Histone acetylase PCAF) (Lysine acetyltransferase 2B) (P300/CBP-associated factor) (P/CAF) (Spermidine acetyltransferase KAT2B) (EC 2.3.1.57)	16.83	0.810
O15264	Mitogen-activated protein kinase 13 (MAP kinase 13) (MAPK 13) (EC 2.7.11.24) (Mitogen-activated protein kinase p38 delta) (MAP kinase p38 delta) (Stress-activated protein kinase 4)	16.71	0.594
Q14671	Pumilio homolog 1 (HsPUM) (Pumilio-1)	16.69	0.875
Q05086	Ubiquitin-protein ligase E3A (EC 2.3.2.26) (E6AP ubiquitin-protein ligase) (HECT-type ubiquitin transferase E3A) (Human papillomavirus E6-associated protein) (Oncogenic protein-associated protein E6-AP) (Renal carcinoma antigen NY-REN-54)	16.57	0.806
P05231	Interleukin-6 (IL-6) (B-cell stimulatory factor 2) (BSF-2) (CTL differentiation factor) (CDF) (Hybridoma growth factor) (Interferon beta-2) (IFN-beta-2)	16.51	0.667
P60903	Protein S100-A10 (Calpactin I light chain) (Calpactin-1 light chain) (Cellular ligand of annexin II) (S100 calcium-binding protein A10) (p10 protein) (p11)	16.49	0.302
Q99816	Tumor susceptibility gene 101 protein (ESCRT-I complex subunit TSG101)	16.41	0.763
Q96FC9	ATP-dependent DNA helicase DDX11 (EC 3.6.4.12) (CHL1-related protein 1) (hCHLR1) (DEAD/H-box protein 11) (Keratinocyte growth factor-regulated gene 2 protein) (KRG-2)	16.39	0.804
P29034	Protein S100-A2 (CAN19) (Protein S-100L) (S100 calcium-binding protein A2)	16.33	0.386
Q08752	Peptidyl-prolyl cis-trans isomerase D (PPIase D) (EC 5.2.1.8) (40 kDa peptidyl-prolyl cis-trans isomerase) (Cyclophilin-40) (CYP-40) (Cyclophilin-related protein) (Rotamase D)	16.22	0.624
P31942	Heterogeneous nuclear ribonucleoprotein H3 (hnRNP H3) (Heterogeneous nuclear ribonucleoprotein 2H9) (hnRNP 2H9)	16.18	0.549
P45452	Collagenase 3 (EC 3.4.24.-) (Matrix metalloproteinase-13) (MMP-13)	16.14	0.496

Supplementary Table S3. Investigation of keratinocyte protein intrinsic disorder in proteome database.

Entry	Protein names	RAPID % Disorder	SLIDER IDR propensity score (0-1)
P05109	Protein S100-A8 (Calgranulin-A) (Calprotectin L1L subunit) (Cystic fibrosis antigen) (CFAG) (Leukocyte L1 complex light chain) (Migration inhibitory factor-related protein 8) (MRP-8) (p8) (S100 calcium-binding protein A8) (Urinary stone protein band A)	16.13	0.186
Q09028	Histone-binding protein RBBP4 (Chromatin assembly factor 1 subunit C) (CAF-1 subunit C) (Chromatin assembly factor I p48 subunit) (CAF-I 48 kDa subunit) (CAF-I p48) (Nucleosome-remodeling factor subunit RBAP48) (Retinoblastoma-binding protein 4) (RBBP-4) (Retinoblastoma-binding protein p48)	15.53	0.455
P08729	Keratin, type II cytoskeletal 7 (Cytokeratin-7) (CK-7) (Keratin-7) (K7) (Sarcolectin) (Type-II keratin Kb7)	15.35	0.837
Q9NPF7	Interleukin-23 subunit alpha (IL-23 subunit alpha) (IL-23-A) (Interleukin-23 subunit p19) (IL-23p19)	15.34	0.546
P15924	Desmoplakin (DP) (250/210 kDa paraneoplastic pemphigus antigen)	17.97	0.916
Q13510	Acid ceramidase (AC) (ACDase) (Acid CDase) (EC 3.5.1.23) (Acylsphingosine deacylase) (N-acylethanolamine hydrolase ASAH1) (EC 3.5.1.-) (N-acylsphingosine amidohydrolase) (Putative 32 kDa heart protein) (PHP32) [Cleaved into: Acid ceramidase subunit alpha; Acid ceramidase subunit beta]	0	0.387
Q9NZT1	Calmodulin-like protein 5 (Calmodulin-like skin protein)	28.77	0.534
O95832	Claudin-1 (Senescence-associated epithelial membrane protein)	10.9	0.272
P08861	Chymotrypsin-like elastase family member 3B (EC 3.4.21.70) (Elastase IIIB) (Elastase-3B) (Protease E)	2.96	0.336
P78545	ETS-related transcription factor Elf-3 (E74-like factor 3) (Epithelial-restricted with serine box) (Epithelium-restricted Ets protein ESX) (Epithelium-specific Ets transcription factor 1) (ESE-1)	29.92	0.729
P19883	Follistatin (FS) (Activin-binding protein)	11.34	0.642
P30793	GTP cyclohydrolase 1 (EC 3.5.4.16) (GTP cyclohydrolase I) (GTP-CH-I)	24.8	0.801
Q9C0K0	B-cell lymphoma/leukemia 11B (BCL-11B) (B-cell CLL/lymphoma 11B) (COUP-TF-interacting protein 2) (Radiation-induced tumor suppressor gene 1 protein) (hRit1)	28.52	0.911
P16070	CD44 antigen (CDw44) (Epican) (Extracellular matrix receptor III) (ECMR-III) (GP90 lymphocyte homing/adhesion receptor) (HUTCH-I) (Heparan sulfate proteoglycan) (Hermes antigen) (Hyaluronate receptor) (Phagocytic glycoprotein 1) (PGP-1) (Phagocytic glycoprotein I) (PGP-I) (CD antigen CD44)	28.3	0.794
Q86XK2	F-box only protein 11 (Protein arginine N-methyltransferase 9) (Vitiligo-associated protein 1) (VIT-1)	15.32	0.751
A6NND4	Olfactory receptor 2AT4 (Olfactory receptor OR11-265)	15.31	0.223
P08779	Keratin, type I cytoskeletal 16 (Cytokeratin-16) (CK-16) (Keratin-16) (K16)	15.22	0.768
P22607	Fibroblast growth factor receptor 3 (FGFR-3) (EC 2.7.10.1) (CD antigen CD333)	15.14	0.658

Supplementary Table S3. Investigation of keratinocyte protein intrinsic disorder in proteome database.

Entry	Protein names	RAPID % Disorder	SLIDER IDR propensity score (0-1)
Q9BWL3	Protein C1orf43 (Hepatitis C virus NS5A-transactivated protein 4) (HCV NS5A-transactivated protein 4) (Protein NICE-3) (S863-3)	15.02	0.567
O14625	C-X-C motif chemokine 11 (Beta-R1) (H174) (Interferon gamma-inducible protein 9) (IP-9) (Interferon-inducible T-cell alpha chemoattractant) (I-TAC) (Small-inducible cytokine B11)	14.89	0.296
P02533	Keratin, type I cytoskeletal 14 (Cytokeratin-14) (CK-14) (Keratin-14) (K14)	14.83	0.767
Q9H3M7	Thioredoxin-interacting protein (Thioredoxin-binding protein 2) (Vitamin D3 up-regulated protein 1)	14.83	0.570
Q14254	Flotillin-2 (Epidermal surface antigen) (ESA) (Membrane component chromosome 17 surface marker 1)	14.72	0.622
Q06330	Recombining binding protein suppressor of hairless (CBF-1) (J kappa-recombination signal-binding protein) (RBP-J kappa) (RBP-J) (RBP-JK) (Renal carcinoma antigen NY-REN-30)	14.6	0.769
P26599	Polypyrimidine tract-binding protein 1 (PTB) (57 kDa RNA-binding protein PPTB-1) (Heterogeneous nuclear ribonucleoprotein I) (hnRNP I)	14.5	0.721
P01135	Protransforming growth factor alpha [Cleaved into: Transforming growth factor alpha (TGF-alpha) (EGF-like TGF) (ETGF) (TGF type 1)]	14.37	0.303
P10599	Thioredoxin (Trx) (ATL-derived factor) (ADF) (Surface-associated sulphydryl protein) (SASP) (allergen Hom s Trx)	14.29	0.235
Q9HBJ0	Placenta-specific protein 1	14.15	0.431
Q9BQL6	Fermitin family homolog 1 (Kindlerin) (Kindlin syndrome protein) (Kindlin-1) (Unc-112-related protein 1)	13.88	0.671
Q99536	Synaptic vesicle membrane protein VAT-1 homolog (EC 1.-.-.-)	13.74	0.663
Q9NY93	Probable ATP-dependent RNA helicase DDX56 (EC 3.6.4.13) (ATP-dependent 61 kDa nucleolar RNA helicase) (DEAD box protein 21) (DEAD box protein 56)	13.71	0.757
Q9BPX6	Calcium uptake protein 1, mitochondrial (Atopy-related autoantigen CALC) (ara CALC) (Calcium-binding atopy-related autoantigen 1) (allergen Hom s 4)	13.66	0.631
Q9Y696	Chloride intracellular channel protein 4 (Intracellular chloride ion channel protein p64H1)	13.44	0.494
Q04695	Keratin, type I cytoskeletal 17 (39.1) (Cytokeratin-17) (CK-17) (Keratin-17) (K17)	13.43	0.768
Q9UIV8	Serpin B13 (HaCaT UV-repressible serpin) (Hurpin) (Headpin) (Peptidase inhibitor 13) (PI-13) (Proteinase inhibitor 13)	13.3	0.612
O95274	Ly6/PLAUR domain-containing protein 3 (GPI-anchored metastasis-associated protein C4.4A homolog) (Matrigel-induced gene C4 protein) (MIG-C4)	13.29	0.694
Q6ZWJ1	Syntaxin-binding protein 4 (Syntaxin 4-interacting protein) (STX4-interacting protein) (Synip)	13.2	0.650
Q9ULD6	Protein inturned (Inturned planar cell polarity effector homolog) (PDZ domain-containing protein 6)	13.16	0.790

Supplementary Table S3. Investigation of keratinocyte protein intrinsic disorder in proteome database.

Entry	Protein names	RAPID % Disorder	SLIDER IDR propensity score (0-1)
P18054	Polyunsaturated fatty acid lipoxygenase ALOX12 (EC 1.13.11.-) (Arachidonate (12S)-lipoxygenase) (12S-LOX) (12S-lipoxygenase) (EC 1.13.11.31) (Arachidonate (15S)-lipoxygenase) (EC 1.13.11.33) (Linoleate (13S)-lipoxygenase) (Lipoxin synthase 12-LO) (EC 3.3.2.-) (Platelet-type lipoxygenase 12)	13.12	0.604
Q96EY1	DnaJ homolog subfamily A member 3, mitochondrial (DnaJ protein Tid-1) (hTid-1) (Hepatocellular carcinoma-associated antigen 57) (Tumorous imaginal discs protein Tid56 homolog)	13.12	0.722
Q92874	Deoxyribonuclease-1-like 2 (EC 3.1.21.-) (DNase I homolog protein DHP1) (Deoxyribonuclease I-like 2) (DNase I-like 2)	13.04	0.485
P22735	Protein-glutamine gamma-glutamyltransferase K (EC 2.3.2.13) (Epidermal TGase) (Transglutaminase K) (TG(K)) (TGK) (TGase K) (Transglutaminase-1) (TGase-1)	12.97	0.713
P19012	Keratin, type I cytoskeletal 15 (Cytokeratin-15) (CK-15) (Keratin-15) (K15)	12.94	0.739
Q92696	Geranylgeranyl transferase type-2 subunit alpha (EC 2.5.1.60) (Geranylgeranyl transferase type II subunit alpha) (Rab geranyl-geranyltransferase subunit alpha) (Rab GG transferase alpha) (Rab GGTase alpha) (Rab geranylgeranyltransferase subunit alpha)	12.87	0.594
Q15119	[Pyruvate dehydrogenase (acetyl-transferring)] kinase isozyme 2, mitochondrial (EC 2.7.11.2) (Pyruvate dehydrogenase kinase isoform 2) (PDH kinase 2) (PDKII)	12.78	0.473
P35237	Serpin B6 (Cytoplasmic antiproteinase) (CAP) (Peptidase inhibitor 6) (PI-6) (Placental thrombin inhibitor)	12.77	0.605
P08727	Keratin, type I cytoskeletal 19 (Cytokeratin-19) (CK-19) (Keratin-19) (K19)	12.75	0.730
Q08345	Epithelial discoidin domain-containing receptor 1 (Epithelial discoidin domain receptor 1) (EC 2.7.10.1) (CD167 antigen-like family member A) (Cell adhesion kinase) (Discoidin receptor tyrosine kinase) (HGK2) (Mammary carcinoma kinase 10) (MCK-10) (Protein-tyrosine kinase 3A) (Protein-tyrosine kinase RTK-6) (TRK E) (Tyrosine kinase DDR) (Tyrosine-protein kinase CAK) (CD antigen CD167a)	12.71	0.757
P08912	Muscarinic acetylcholine receptor M5	12.59	0.688
Q01469	Fatty acid-binding protein 5 (Epidermal-type fatty acid-binding protein) (E-FABP) (Fatty acid-binding protein, epidermal) (Psoriasis-associated fatty acid-binding protein homolog) (PA-FABP)	12.59	0.350
Q15323	Keratin, type I cuticular Ha1 (Hair keratin, type I Ha1) (Keratin-31) (K31)	12.5	0.704
P05120	Plasminogen activator inhibitor 2 (PAI-2) (Monocyte Arg-serpin) (Placental plasminogen activator inhibitor) (Serpin B2) (Urokinase inhibitor)	12.29	0.567
Q9NZH7	Interleukin-36 beta (FIL1 eta) (Interleukin-1 eta) (IL-1 eta) (Interleukin-1 family member 8) (IL-1F8) (Interleukin-1 homolog 2) (IL-1H2)	12.2	0.373
Q8TDN7	Alkaline ceramidase 1 (AlkCDase 1) (Alkaline CDase 1) (EC 3.5.1.-) (EC 3.5.1.23) (Acylsphingosine deacylase 3) (N-acylsphingosine amidohydrolase 3)	12.12	0.109
Q8N474	Secreted frizzled-related protein 1 (FRP-1) (sFRP-1) (Secreted apoptosis-related protein 2) (SARP-2)	12.1	0.721
Q7L5A8	Fatty acid 2-hydroxylase (EC 1.14.18.-) (Fatty acid alpha-hydroxylase) (Fatty acid hydroxylase domain-containing protein 1)	12.1	0.430

Supplementary Table S3. Investigation of keratinocyte protein intrinsic disorder in proteome database.

Entry	Protein names	RAPID % Disorder	SLIDER IDR propensity score (0-1)
Q9UH17	DNA dC->dU-editing enzyme APOBEC-3B (A3B) (EC 3.5.4.38) (Phorbolin-1-related protein) (Phorbolin-2/3)	12.04	0.214
Q13751	Laminin subunit beta-3 (Epiligrin subunit bata) (Kalinin B1 chain) (Kalinin subunit beta) (Laminin B1k chain) (Laminin-5 subunit beta) (Nicein subunit beta)	12.03	0.815
Q8IU89	Ceramide synthase 3 (CerS3) (EC 2.3.1.-) (Dihydroceramide synthase 3) (LAG1 longevity assurance homolog 3) (Sphingosine N-acyltransferase CERS3) (EC 2.3.1.24)	12.01	0.479
Q01432	AMP deaminase 3 (EC 3.5.4.6) (AMP deaminase isoform E) (Erythrocyte AMP deaminase)	11.99	0.769
P31146	Coronin-1A (Coronin-like protein A) (Clipin-A) (Coronin-like protein p57) (Tryptophan aspartate-containing coat protein) (TACO)	11.93	0.540
P19013	Keratin, type II cytoskeletal 4 (Cytokeratin-4) (CK-4) (Keratin-4) (K4) (Type-II keratin Kb4)	11.92	0.761
P21781	Fibroblast growth factor 7 (FGF-7) (Heparin-binding growth factor 7) (HBGF-7) (Keratinocyte growth factor)	11.86	0.389
P63241	Eukaryotic translation initiation factor 5A-1 (eIF-5A-1) (eIF-5A1) (Eukaryotic initiation factor 5A isoform 1) (eIF-5A) (Rev-binding factor) (eIF-4D)	11.69	0.458
P13647	Keratin, type II cytoskeletal 5 (58 kDa cytokeratin) (Cytokeratin-5) (CK-5) (Keratin-5) (K5) (Type-II keratin Kb5)	11.69	0.792
Q8N565	Melanoregulin (Dilute suppressor protein homolog)	11.68	0.654
P55000	Secreted Ly-6/uPAR-related protein 1 (SLURP-1) (ARS component B) (ARS(component B)-81/S) (Anti-neoplastic urinary protein) (ANUP)	11.65	0.288
P84022	Mothers against decapentaplegic homolog 3 (MAD homolog 3) (Mad3) (Mothers against DPP homolog 3) (hMAD-3) (JV15-2) (SMAD family member 3) (SMAD 3) (Smad3) (hSMAD3)	11.53	0.641
P60953	Cell division control protein 42 homolog (EC 3.6.5.2) (G25K GTP-binding protein)	11.52	0.372
Q6UW15	Regenerating islet-derived protein 3-gamma (REG-3-gamma) (Pancreatitis-associated protein 1B) (PAP-1B) (Pancreatitis-associated protein IB) (PAP IB) (Regenerating islet-derived protein III-gamma) (REG III) (Reg III-gamma) [Cleaved into: Regenerating islet-derived protein 3-gamma 16.5 kDa form; Regenerating islet-derived protein 3-gamma 15 kDa form]	11.43	0.377
Q9HBA0	Transient receptor potential cation channel subfamily V member 4 (TrpV4) (Osm-9-like TRP channel 4) (OTRPC4) (Transient receptor potential protein 12) (TRP12) (Vanilloid receptor-like channel 2) (Vanilloid receptor-like protein 2) (VRL-2) (Vanilloid receptor-related osmotically-activated channel) (VR-OAC)	11.37	0.657
Q9C000	NACHT, LRR and PYD domains-containing protein 1 (EC 3.4.-.-) (EC 3.6.4.-) (Caspase recruitment domain-containing protein 7) (Death effector filament-forming ced-4-like apoptosis protein) (Nucleotide-binding domain and caspase recruitment domain) [Cleaved into: NACHT, LRR and PYD domains-containing protein 1, C-terminus (NLRP1-CT); NACHT, LRR and PYD domains-containing protein 1, N-terminus (NLRP1-NT)]	11.34	0.743
P06396	Gelsolin (AGEL) (Actin-depolymerizing factor) (ADF) (Brevin)	11.25	0.679
O75369	Filamin-B (FLN-B) (ABP-278) (ABP-280 homolog) (Actin-binding-like protein) (Beta-filamin) (Filamin homolog 1) (Fh1) (Filamin-3) (Thyroid autoantigen) (Truncated actin-binding protein) (Truncated ABP)	11.18	0.673

Supplementary Table S3. Investigation of keratinocyte protein intrinsic disorder in proteome database.

Entry	Protein names	RAPID % Disorder	SLIDER IDR propensity score (0-1)
O76013	Keratin, type I cuticular Ha6 (Hair keratin, type I Ha6) (Keratin-36) (K36)	11.13	0.658
P40933	Interleukin-15 (IL-15)	11.11	0.360
Q99497	Parkinson disease protein 7 (Maillard deglycase) (Oncogene DJ1) (Parkinsonism-associated deglycase) (Protein DJ-1) (DJ-1) (Protein/nucleic acid deglycase DJ-1) (EC 3.1.2.-) (EC 3.5.1.-) (EC 3.5.1.124)	11.11	0.377
O15520	Fibroblast growth factor 10 (FGF-10) (Keratinocyte growth factor 2)	11.06	0.517
P11142	Heat shock cognate 71 kDa protein (EC 3.6.4.10) (Heat shock 70 kDa protein 8) (Lipopolysaccharide-associated protein 1) (LAP-1) (LPS-associated protein 1)	10.99	0.741
P05230	Fibroblast growth factor 1 (FGF-1) (Acidic fibroblast growth factor) (aFGF) (Endothelial cell growth factor) (ECGF) (Heparin-binding growth factor 1) (HBGF-1)	10.97	0.425
Q8NFR9	Interleukin-17 receptor E (IL-17 receptor E) (IL-17RE)	10.94	0.664
Q6QHC5	Sphingolipid delta(4)-desaturase/C4-monooxygenase DES2 (EC 1.14.18.5) (EC 1.14.19.17) (Degenerative spermatocyte homolog 2) (Sphingolipid 4-desaturase) (Sphingolipid C4-monooxygenase)	10.84	0.225
Q15139	Serine/threonine-protein kinase D1 (EC 2.7.11.13) (Protein kinase C mu type) (Protein kinase D) (nPKC-D1) (nPKC-mu)	10.75	0.818
P30041	Peroxiredoxin-6 (EC 1.11.1.27) (1-Cys peroxiredoxin) (1-Cys PRX) (24 kDa protein) (Acidic calcium-independent phospholipase A2) (aiPLA2) (EC 3.1.1.4) (Antioxidant protein 2) (Glutathione-dependent peroxiredoxin) (Liver 2D page spot 40) (Lysophosphatidylcholine acyltransferase 5) (LPC acyltransferase 5) (LPCAT-5) (Lyso-PC acyltransferase 5) (EC 2.3.1.23) (Non-selenium glutathione peroxidase) (NSGPx) (Red blood cells page spot 12)	10.71	0.385
P13646	Keratin, type I cytoskeletal 13 (Cytokeratin-13) (CK-13) (Keratin-13) (K13)	10.7	0.793
P04083	Annexin A1 (Annexin I) (Annexin-1) (Calpactin II) (Calpactin-2) (Chromobindin-9) (Lipocortin I) (Phospholipase A2 inhibitory protein) (p35)	10.69	0.584
P30740	Leukocyte elastase inhibitor (LEI) (Monocyte/neutrophil elastase inhibitor) (EI) (M/NEI) (Peptidase inhibitor 2) (PI-2) (Serpin B1)	10.55	0.619
P02538	Keratin, type II cytoskeletal 6A (Cytokeratin-6A) (CK-6A) (Cytokeratin-6D) (CK-6D) (Keratin-6A) (K6A) (Type-II keratin Kb6) (allergen Hom s 5)	10.46	0.762
P16050	Polyunsaturated fatty acid lipoxygenase ALOX15 (12/15-lipoxygenase) (Arachidonate 12-lipoxygenase, leukocyte-type) (12-LOX) (EC 1.13.11.31) (Arachidonate 15-lipoxygenase) (15-LOX) (15-LOX-1) (EC 1.13.11.33) (Arachidonate omega-6 lipoxygenase) (Hepoxilin A3 synthase Alox15) (EC 1.13.11.-) (Linoleate 13S-lipoxygenase) (EC 1.13.11.12)	10.42	0.544
Q5T750	Skin-specific protein 32	10.4	0.561
P28066	Proteasome subunit alpha type-5 (Macropain zeta chain) (Multicatalytic endopeptidase complex zeta chain) (Proteasome zeta chain)	10.37	0.414
Q9H5V8	CUB domain-containing protein 1 (Membrane glycoprotein gp140) (Subtractive immunization M plus HEp3-associated 135 kDa protein) (SIMA135) (Transmembrane and associated with src kinases) (CD antigen CD318)	10.29	0.678

Supplementary Table S3. Investigation of keratinocyte protein intrinsic disorder in proteome database.

Entry	Protein names	RAPID % Disorder	SLIDER IDR propensity score (0-1)
P04259	Keratin, type II cytoskeletal 6B (Cytokeratin-6B) (CK-6B) (Keratin-6B) (K6B) (Type-II keratin Kb10)	10.28	0.776
Q9UNE0	Tumor necrosis factor receptor superfamily member EDAR (Anhidrotic ectodysplasin receptor 1) (Downless homolog) (EDA-A1 receptor) (Ectodermal dysplasia receptor) (Ectodysplasin-A receptor)	10.27	0.750
Q13049	E3 ubiquitin-protein ligase TRIM32 (EC 2.3.2.27) (72 kDa Tat-interacting protein) (RING-type E3 ubiquitin transferase TRIM32) (Tripartite motif-containing protein 32) (Zinc finger protein HT2A)	10.26	0.694
Q9NPG1	Frizzled-3 (Fz-3) (hFz3)	10.21	0.518
Q9Y6W6	Dual specificity protein phosphatase 10 (EC 3.1.3.16) (EC 3.1.3.48) (Mitogen-activated protein kinase phosphatase 5) (MAP kinase phosphatase 5) (MKP-5)	10.17	0.718
O76027	Annexin A9 (Annexin XXXI) (Annexin-31) (Annexin-9) (Pemphaxin)	10.14	0.491
O75342	Arachidonate 12-lipoxygenase, 12R-type (12R-LOX) (12R-lipoxygenase) (EC 1.13.11.-) (Epidermis-type lipoxygenase 12)	10.13	0.508
P31943	Heterogeneous nuclear ribonucleoprotein H (hnRNP H) [Cleaved into: Heterogeneous nuclear ribonucleoprotein H, N-terminally processed]	10.02	0.590
P58107	Epiplakin (450 kDa epidermal antigen)	10.02	0.846
Q53RY4	Keratinocyte-associated protein 3 (KCP-3)	10	0.552
P68363	Tubulin alpha-1B chain (Alpha-tubulin ubiquitous) (Tubulin K-alpha-1) (Tubulin alpha-ubiquitous chain) [Cleaved into: Detyrosinated tubulin alpha-1B chain]	9.98	0.507
Q99828	Calcium and integrin-binding protein 1 (CIB) (Calcium- and integrin-binding protein) (CIBP) (Calmyrin) (DNA-PKcs-interacting protein) (Kinase-interacting protein) (KIP) (SNK-interacting protein 2-28) (SIP2-28)	9.95	0.616
P07947	Tyrosine-protein kinase Yes (EC 2.7.10.2) (Proto-oncogene c-Yes) (p61-Yes)	9.94	0.554
Q9BY79	Membrane frizzled-related protein (Membrane-type frizzled-related protein)	9.84	0.626
Q969P0	Immunoglobulin superfamily member 8 (IgSF8) (CD81 partner 3) (Glu-Trp-Ile EWI motif-containing protein 2) (EWI-2) (Keratinocytes-associated transmembrane protein 4) (KCT-4) (LIR-D1) (Prostaglandin regulatory-like protein) (PGRL) (CD antigen CD316)	9.79	0.506
Q9UKW4	Guanine nucleotide exchange factor VAV3 (VAV-3)	9.68	0.698
Q13753	Laminin subunit gamma-2 (Cell-scattering factor 140 kDa subunit) (CSF 140 kDa subunit) (Epiligrin subunit gamma) (Kalinin subunit gamma) (Kalinin/nicein/epiligrin 100 kDa subunit) (Ladsin 140 kDa subunit) (Laminin B2t chain) (Laminin-5 subunit gamma) (Large adhesive scatter factor 140 kDa subunit) (Nicein subunit gamma)	9.64	0.796
O15296	Polyunsaturated fatty acid lipoxygenase ALOX15B (15-lipoxygenase 2) (15-LOX-2) (Arachidonate 15-lipoxygenase B) (15-LOX-B) (EC 1.13.11.33) (Arachidonate 15-lipoxygenase type II) (Linoleate 13-lipoxygenase 15-LOb) (EC 1.13.11.-)	9.62	0.527

Supplementary Table S3. Investigation of keratinocyte protein intrinsic disorder in proteome database.

Entry	Protein names	RAPID % Disorder	SLIDER IDR propensity score (0-1)
P31941	DNA dC->dU-editing enzyme APOBEC-3A (A3A) (EC 3.5.4.38) (Phorbolin-1)	9.55	0.254
P09936	Ubiquitin carboxyl-terminal hydrolase isozyme L1 (UCH-L1) (EC 3.4.19.12) (Neuron cytoplasmic protein 9.5) (PGP 9.5) (PGP9.5) (Ubiquitin thioesterase L1)	9.42	0.508
Q16739	Ceramide glucosyltransferase (EC 2.4.1.80) (GLCT-1) (Glucosylceramide synthase) (GCS) (UDP-glucose ceramide glucosyltransferase) (UDP-glucose:N-acylsphingosine D-glucosyltransferase)	9.39	0.283
P42345	Serine/threonine-protein kinase mTOR (EC 2.7.11.1) (FK506-binding protein 12-rapamycin complex-associated protein 1) (FKBP12-rapamycin complex-associated protein) (Mammalian target of rapamycin) (mTOR) (Mechanistic target of rapamycin) (Rapamycin and FKBP12 target 1) (Rapamycin target protein 1)	9.34	0.809
Q99683	Mitogen-activated protein kinase kinase kinase 5 (EC 2.7.11.25) (Apoptosis signal-regulating kinase 1) (ASK-1) (MAPK/ERK kinase kinase 5) (MEK kinase 5) (MEKK 5)	9.24	0.772
Q8NET8	Transient receptor potential cation channel subfamily V member 3 (TrpV3) (Vanilloid receptor-like 3) (VRL-3)	9.24	0.709
P37088	Amiloride-sensitive sodium channel subunit alpha (Alpha-NaCH) (Epithelial Na(+) channel subunit alpha) (Alpha-ENaC) (ENaCA) (Nonvoltage-gated sodium channel 1 subunit alpha) (SCNEA)	9.12	0.703
O15528	25-hydroxyvitamin D-1 alpha hydroxylase, mitochondrial (EC 1.14.15.18) (25-OHD-1 alpha-hydroxylase) (25-hydroxyvitamin D(3) 1-alpha-hydroxylase) (VD3 1A hydroxylase) (Calcidiol 1-monooxygenase) (Cytochrome P450 subfamily XXVIIB polypeptide 1) (Cytochrome P450C1 alpha) (Cytochrome P450VD1-alpha) (Cytochrome p450 27B1)	9.06	0.589
P01375	Tumor necrosis factor (Cachectin) (TNF-alpha) (Tumor necrosis factor ligand superfamily member 2) (TNF-a) [Cleaved into: Tumor necrosis factor, membrane form (N-terminal fragment) (NTF); Intracellular domain 1 (ICD1); Intracellular domain 2 (ICD2); C-domain 1; C-domain 2; Tumor necrosis factor, soluble form]	9.01	0.402
P01584	Interleukin-1 beta (IL-1 beta) (Catabolin)	8.92	0.570
Q9H190	Syntenin-2 (Similar to TACIP18) (SITAC) (Syndecan-binding protein 2)	8.9	0.523
Q9NZH8	Interleukin-36 gamma (IL-1-related protein 2) (IL-1RP2) (Interleukin-1 epsilon) (IL-1 epsilon) (Interleukin-1 family member 9) (IL-1F9) (Interleukin-1 homolog 1) (IL-1H1)	8.88	0.364
O60259	Kallikrein-8 (hK8) (EC 3.4.21.118) (Neuropsin) (NP) (Ovasin) (Serine protease 19) (Serine protease TADG-14) (Tumor-associated differentially expressed gene 14 protein)	8.85	0.516
Q96FA3	E3 ubiquitin-protein ligase pellino homolog 1 (Pellino-1) (EC 2.3.2.27) (Pellino-related intracellular-signaling molecule) (RING-type E3 ubiquitin transferase pellino homolog 1)	8.85	0.498
Q8TAT5	Endonuclease 8-like 3 (EC 3.2.2.-) (EC 4.2.99.18) (DNA glycosylase FPG2) (DNA glycosylase/AP lyase Neil3) (Endonuclease VIII-like 3) (Nei-like protein 3)	8.76	0.602
P01137	Transforming growth factor beta-1 proprotein [Cleaved into: Latency-associated peptide (LAP); Transforming growth factor beta-1 (TGF-beta-1)]	8.72	0.633
O43820	Hyaluronidase-3 (Hyal-3) (EC 3.2.1.35) (Hyaluronoglucosaminidase-3) (Lung carcinoma protein 3) (LuCa-3)	8.63	0.403

Supplementary Table S3. Investigation of keratinocyte protein intrinsic disorder in proteome database.

Entry	Protein names	RAPID % Disorder	SLIDER IDR propensity score (0-1)
Q8TAF3	WD repeat-containing protein 48 (USP1-associated factor 1) (WD repeat endosomal protein) (p80)	8.57	0.713
P22392	Nucleoside diphosphate kinase B (NDK B) (NDP kinase B) (EC 2.7.4.6) (C-myc purine-binding transcription factor PUF) (Histidine protein kinase NDKB) (EC 2.7.13.3) (nm23-H2)	8.55	0.260
Q9UL19	Phospholipase A and acyltransferase 4 (EC 2.3.1.-) (EC 3.1.1.32) (EC 3.1.1.4) (HRAS-like suppressor 4) (HRSL4) (RAR-responsive protein TIG3) (Retinoic acid receptor responder protein 3) (Retinoid-inducible gene 1 protein) (Tazarotene-induced gene 3 protein)	8.54	0.405
P23381	Tryptophan--tRNA ligase, cytoplasmic (EC 6.1.1.2) (Interferon-induced protein 53) (IFP53) (Tryptophanyl-tRNA synthetase) (TrpRS) (hWRS) [Cleaved into: T1-TrpRS; T2-TrpRS]	8.49	0.541
P42330	Aldo-keto reductase family 1 member C3 (EC 1.1.1.-) (EC 1.1.1.210) (EC 1.1.1.53) (EC 1.1.1.62) (17-beta-hydroxysteroid dehydrogenase type 5) (17-beta-HSD 5) (3-alpha-HSD type II, brain) (3-alpha-hydroxysteroid dehydrogenase type 2) (3-alpha-HSD type 2) (EC 1.1.1.357) (Chlordecone reductase homolog HAKRb) (Dihydrodiol dehydrogenase 3) (DD-3) (DD3) (Dihydrodiol dehydrogenase type I) (HA1753) (Prostaglandin F synthase) (PGFS) (EC 1.1.1.188) (Testosterone 17-beta-dehydrogenase 5) (EC 1.1.1.239) (EC 1.1.1.64)	8.36	0.385
Q9UGM1	Neuronal acetylcholine receptor subunit alpha-9 (Nicotinic acetylcholine receptor subunit alpha-9) (NACHR alpha-9)	8.35	0.440
Q13618	Cullin-3 (CUL-3)	8.33	0.744
O14944	Proepiregulin [Cleaved into: Epiregulin (EPR)]	8.28	0.405
Q7Z403	Transmembrane channel-like protein 6 (Epidermodysplasia verruciformis protein 1) (Protein LAK-4)	8.2	0.632
Q02413	Desmoglein-1 (Cadherin family member 4) (Desmosomal glycoprotein 1) (DG1) (DGI) (Pemphigus foliaceus antigen)	8.1	0.754
P10155	60 kDa SS-A/Ro ribonucleoprotein (60 kDa Ro protein) (60 kDa ribonucleoprotein Ro) (Ro60) (RoRNP) (Ro 60 kDa autoantigen) (Ro60 autoantigen) (Sjoegren syndrome antigen A2) (Sjoegren syndrome type A antigen) (SS-A) (TROVE domain family member 2)	7.99	0.604
Q8NI37	Protein phosphatase PTC7 homolog (EC 3.1.3.16) (T-cell activation protein phosphatase 2C) (TA-PP2C) (T-cell activation protein phosphatase 2C-like)	7.89	0.475
P07711	Procathepsin L (EC 3.4.22.15) (Cathepsin L1) (Major excreted protein) (MEP) [Cleaved into: Cathepsin L; Cathepsin L heavy chain; Cathepsin L light chain]	7.81	0.403
Q9UBH0	Interleukin-36 receptor antagonist protein (IL-36Ra) (FIL1 delta) (IL-1-related protein 3) (IL-1RP3) (Interleukin-1 HY1) (IL-1HY1) (Interleukin-1 delta) (IL-1 delta) (Interleukin-1 family member 5) (IL-1F5) (Interleukin-1 receptor antagonist homolog 1) (IL-1ra homolog 1) (Interleukin-1-like protein 1) (IL-1L1)	7.74	0.232
Q16828	Dual specificity protein phosphatase 6 (EC 3.1.3.16) (EC 3.1.3.48) (Dual specificity protein phosphatase PYST1) (Mitogen-activated protein kinase phosphatase 3) (MAP kinase phosphatase 3) (MKP-3)	7.61	0.674
P30626	Sorcin (22 kDa protein) (CP-22) (CP22) (V19)	7.58	0.332
P37173	TGF-beta receptor type-2 (TGFR-2) (EC 2.7.11.30) (TGF-beta type II receptor) (Transforming growth factor-beta receptor type II) (TGF-beta receptor type II) (TbetaR-II)	7.58	0.484

Supplementary Table S3. Investigation of keratinocyte protein intrinsic disorder in proteome database.

Entry	Protein names	RAPID % Disorder	SLIDER IDR propensity score (0-1)
Q29983	MHC class I polypeptide-related sequence A (MIC-A)	7.57	0.531
P16144	Integrin beta-4 (GP150) (CD antigen CD104)	7.57	0.828
Q04828	Aldo-keto reductase family 1 member C1 (EC 1.1.1.-) (EC 1.1.1.112) (EC 1.1.1.209) (EC 1.1.1.210) (EC 1.1.1.357) (EC 1.1.1.51) (EC 1.1.1.53) (EC 1.1.1.62) (EC 1.3.1.20) (20-alpha-hydroxysteroid dehydrogenase) (20-alpha-HSD) (EC 1.1.1.149) (Chlordecone reductase homolog HAKRC) (Dihydrodiol dehydrogenase 1) (DD1) (High-affinity hepatic bile acid-binding protein) (HBAB)	7.43	0.357
Q9BSR8	Protein YIPF4 (YIP1 family member 4)	7.38	0.244
P22455	Fibroblast growth factor receptor 4 (FGFR-4) (EC 2.7.10.1) (CD antigen CD334)	7.36	0.753
Q04721	Neurogenic locus notch homolog protein 2 (Notch 2) (hN2) [Cleaved into: Notch 2 extracellular truncation (N2ECD); Notch 2 intracellular domain (N2ICD)]	7.32	0.782
P49720	Proteasome subunit beta type-3 (Proteasome chain 13) (Proteasome component C10-II) (Proteasome theta chain)	7.32	0.339
Q9NY84	Vascular non-inflammatory molecule 3 (Vanin-3) (EC 3.5.1.92)	7.3	0.329
Q14680	Maternal embryonic leucine zipper kinase (hMELK) (EC 2.7.11.1) (Protein kinase Eg3) (pEg3 kinase) (Protein kinase PK38) (hPK38) (Tyrosine-protein kinase MELK) (EC 2.7.10.2)	7.22	0.644
Q9UKW6	ETS-related transcription factor Elf-5 (E74-like factor 5) (Epithelium-restricted ESE-1-related Ets factor) (Epithelium-specific Ets transcription factor 2) (ESE-2)	7.17	0.367
Q13635	Protein patched homolog 1 (PTC) (PTC1)	7.12	0.736
Q13740	CD166 antigen (Activated leukocyte cell adhesion molecule) (CD antigen CD166)	7.03	0.627
P49721	Proteasome subunit beta type-2 (Macropain subunit C7-I) (Multicatalytic endopeptidase complex subunit C7-I) (Proteasome component C7-I)	6.97	0.390
P36544	Neuronal acetylcholine receptor subunit alpha-7	6.97	0.428
Q8NI17	Interleukin-31 receptor subunit alpha (IL-31 receptor subunit alpha) (IL-31R subunit alpha) (IL-31R-alpha) (IL-31RA) (Cytokine receptor-like 3) (GLM-R) (hGLM-R) (Gp130-like monocyte receptor) (Gp130-like receptor) (ZcytoR17)	6.97	0.659
O43909	Exostosin-like 3 (EC 2.4.1.223) (EXT-related protein 1) (Glucuronyl-galactosyl-proteoglycan 4-alpha-N-acetylglucosaminyltransferase) (Hereditary multiple exostoses gene isolog) (Multiple exostosis-like protein 3) (Putative tumor suppressor protein EXTL3)	6.96	0.628
P14780	Matrix metalloproteinase-9 (MMP-9) (EC 3.4.24.35) (92 kDa gelatinase) (92 kDa type IV collagenase) (Gelatinase B) (GELB) [Cleaved into: 67 kDa matrix metalloproteinase-9; 82 kDa matrix metalloproteinase-9]	6.93	0.632
Q06141	Regenerating islet-derived protein 3-alpha (REG-3-alpha) (Hepatointestinal pancreatic protein) (HIP/PAP) (Human proislet peptide) (Pancreatitis-associated protein 1) (Regenerating islet-derived protein III-alpha) (Reg III-alpha) [Cleaved into: Regenerating islet-derived protein 3-alpha 16.5 kDa form; Regenerating islet-derived protein 3-alpha 15 kDa form]	6.86	0.428

Supplementary Table S3. Investigation of keratinocyte protein intrinsic disorder in proteome database.

Entry	Protein names	RAPID % Disorder	SLIDER IDR propensity score (0-1)
P42574	Caspase-3 (CASP-3) (EC 3.4.22.56) (Apopain) (Cysteine protease CPP32) (CPP-32) (Protein Yama) (SREBP cleavage activity 1) (SCA-1) [Cleaved into: Caspase-3 subunit p17; Caspase-3 subunit p12]	6.86	0.432
Q6UQ28	Placenta-expressed transcript 1 protein	6.76	0.339
P05121	Plasminogen activator inhibitor 1 (PAI) (PAI-1) (Endothelial plasminogen activator inhibitor) (Serpin E1)	6.72	0.469
Q14956	Transmembrane glycoprotein NMB (Hematopoietic growth factor inducible neurokinin-1 type)	6.64	0.562
P22413	Ectonucleotide pyrophosphatase/phosphodiesterase family member 1 (E-NPP 1) (Membrane component chromosome 6 surface marker 1) (Phosphodiesterase I/nucleotide pyrophosphatase 1) (Plasma-cell membrane glycoprotein PC-1) [Cleaved into: Ectonucleotide pyrophosphatase/phosphodiesterase family member 1, secreted form] [Includes: Alkaline phosphodiesterase I (EC 3.1.4.1); Nucleotide pyrophosphatase (NPPase) (EC 3.6.1.9) (Nucleotide diphosphatase)]	6.59	0.706
O75326	Semaphorin-7A (CDw108) (JMH blood group antigen) (John-Milton-Hargen human blood group Ag) (Semaphorin-K1) (Sema K1) (Semaphorin-L) (Sema L) (CD antigen CD108)	6.46	0.691
Q8NER1	Transient receptor potential cation channel subfamily V member 1 (TrpV1) (Capsaicin receptor) (Osm-9-like TRP channel 1) (OTRPC1) (Vanilloid receptor 1)	6.44	0.602
Q16787	Laminin subunit alpha-3 (Epiligrin 170 kDa subunit) (E170) (Epiligrin subunit alpha) (Kalinin subunit alpha) (Laminin-5 subunit alpha) (Laminin-6 subunit alpha) (Laminin-7 subunit alpha) (Nicein subunit alpha)	6.33	0.798
P29279	CCN family member 2 (Cellular communication network factor 2) (Connective tissue growth factor) (Hypertrophic chondrocyte-specific protein 24) (Insulin-like growth factor-binding protein 8) (IBP-8) (IGF-binding protein 8) (IGFBP-8)	6.3	0.487
Q9UJQ1	Lysosome-associated membrane glycoprotein 5 (Brain and dendritic cell-associated LAMP) (Brain-associated LAMP-like protein) (BAD-LAMP) (Lysosome-associated membrane protein 5) (LAMP-5)	6.07	0.454
P32119	Peroxiredoxin-2 (EC 1.11.1.24) (Natural killer cell-enhancing factor B) (NKEF-B) (PRP) (Thiol-specific antioxidant protein) (TSA) (Thioredoxin peroxidase 1) (Thioredoxin-dependent peroxide reductase 1) (Thioredoxin-dependent peroxiredoxin 2)	6.06	0.256
Q15262	Receptor-type tyrosine-protein phosphatase kappa (Protein-tyrosine phosphatase kappa) (R-PTP-kappa) (EC 3.1.3.48)	5.7	0.659
Q04912	Macrophage-stimulating protein receptor (MSP receptor) (EC 2.7.10.1) (CDw136) (Protein-tyrosine kinase 8) (p185-Ron) (CD antigen CD136) [Cleaved into: Macrophage-stimulating protein receptor alpha chain; Macrophage-stimulating protein receptor beta chain]	5.64	0.646
Q08380	Galectin-3-binding protein (Basement membrane autoantigen p105) (Lectin galactoside-binding soluble 3-binding protein) (Mac-2-binding protein) (MAC2BP) (Mac-2 BP) (Tumor-associated antigen 90K)	5.64	0.498
P07384	Calpain-1 catalytic subunit (EC 3.4.22.52) (Calcium-activated neutral proteinase 1) (CANP 1) (Calpain mu-type) (Calpain-1 large subunit) (Cell proliferation-inducing gene 30 protein) (Micromolar-calpain) (muCANP)	5.6	0.514
P46531	Neurogenic locus notch homolog protein 1 (Notch 1) (hN1) (Translocation-associated notch protein TAN-1) [Cleaved into: Notch 1 extracellular truncation (NEXT); Notch 1 intracellular domain (NICD)]	5.56	0.817
Q9NZQ7	Programmed cell death 1 ligand 1 (PD-L1) (PDCD1 ligand 1) (Programmed death ligand 1) (hPD-L1) (B7 homolog 1) (B7-H1) (CD antigen CD274)	5.52	0.405

Supplementary Table S3. Investigation of keratinocyte protein intrinsic disorder in proteome database.

Entry	Protein names	RAPID % Disorder	SLIDER IDR propensity score (0-1)
Q9NSB2	Keratin, type II cuticular Hb4 (Keratin-84) (K84) (Type II hair keratin Hb4) (Type-II keratin Kb24)	5.5	0.733
P29460	Interleukin-12 subunit beta (IL-12B) (Cytotoxic lymphocyte maturation factor 40 kDa subunit) (CLMF p40) (IL-12 subunit p40) (NK cell stimulatory factor chain 2) (NKSF2)	5.49	0.526
Q92482	Aquaporin-3 (AQP-3) (Aquaglyceroporin-3)	5.48	0.144
Q5VYY2	Lipase member M (EC 3.1.1.-) (Lipase-like abhydrolase domain-containing protein 3)	5.44	0.360
P31939	Bifunctional purine biosynthesis protein ATIC (AICAR transformylase/inosine monophosphate cyclohydrolase) (ATIC) [Cleaved into: Bifunctional purine biosynthesis protein ATIC, N-terminally processed] [Includes: Phosphoribosylaminoimidazolecarboxamide formyltransferase (EC 2.1.2.3) (5-aminoimidazole-4-carboxamide ribonucleotide formyltransferase) (AICAR formyltransferase) (AICAR transformylase); Inosine 5'-monophosphate cyclohydrolase (IMP cyclohydrolase) (EC 3.5.4.10) (IMP synthase) (Inosinicase)]	5.41	0.473
O75354	Ectonucleoside triphosphate diphosphohydrolase 6 (NTPDase 6) (EC 3.6.1.6) (CD39 antigen-like 2)	5.37	0.493
P15529	Membrane cofactor protein (TLX) (Trophoblast leukocyte common antigen) (CD antigen CD46)	5.36	0.546
Q0D2K0	Magnesium transporter NIPA4 (Ichthyin) (NIPA-like protein 4) (Non-imprinted in Prader-Willi/Angelman syndrome region protein 4)	5.36	0.463
Q8IZY2	Phospholipid-transporting ATPase ABCA7 (EC 7.6.2.1) (ABCA-SSN) (ATP-binding cassette sub-family A member 7) (Autoantigen SS-N) (Macrophage ABC transporter)	5.31	0.744
Q13443	Disintegrin and metalloproteinase domain-containing protein 9 (ADAM 9) (EC 3.4.24.-) (Cellular disintegrin-related protein) (Meltrin-gamma) (Metalloprotease/disintegrin/cysteine-rich protein 9) (Myeloma cell metalloproteinase)	5.25	0.669
Q8IU68	Transmembrane channel-like protein 8 (Epidermodysplasia verruciformis protein 2)	5.23	0.635
P63000	Ras-related C3 botulinum toxin substrate 1 (EC 3.6.5.2) (Cell migration-inducing gene 5 protein) (Ras-like protein TC25) (p21-Rac1)	5.21	0.344
Q6UW88	Epigen (Epithelial mitogen) (EPG)	5.19	0.242
P78504	Protein jagged-1 (Jagged1) (hJ1) (CD antigen CD339)	5.17	0.652
P32297	Neuronal acetylcholine receptor subunit alpha-3	5.15	0.525
O43548	Protein-glutamine gamma-glutamyltransferase 5 (EC 2.3.2.13) (Transglutaminase X) (TG(X)) (TGX) (TGase X) (Transglutaminase-5) (TGase-5)	5.14	0.486
P49862	Kallikrein-7 (hK7) (EC 3.4.21.117) (Serine protease 6) (Stratum corneum chymotryptic enzyme) (hSCCE)	5.14	0.515
Q9NYY1	Interleukin-20 (IL-20) (Cytokine Zcyto10)	5.11	0.482

Supplementary Table S3. Investigation of keratinocyte protein intrinsic disorder in proteome database.

Entry	Protein names	RAPID % Disorder	SLIDER IDR propensity score (0-1)
Q9UHA7	Interleukin-36 alpha (FIL1 epsilon) (Interleukin-1 epsilon) (IL-1 epsilon) (Interleukin-1 family member 6) (IL-1F6)	5.06	0.397
Q9UDY8	Mucosa-associated lymphoid tissue lymphoma translocation protein 1 (EC 3.4.22.-) (MALT lymphoma-associated translocation) (Paracaspase)	4.98	0.696
Q9ULW8	Protein-arginine deiminase type-3 (EC 3.5.3.15) (Peptidylarginine deiminase III) (Protein-arginine deiminase type III)	4.97	0.379
Q76B58	BMP/retinoic acid-inducible neural-specific protein 3 (DBCCR1-like protein 1)	4.96	0.566
P31150	Rab GDP dissociation inhibitor alpha (Rab GDI alpha) (Guanosine diphosphate dissociation inhibitor 1) (GDI-1) (Oligophrenin-2) (Protein XAP-4)	4.92	0.520
P41180	Extracellular calcium-sensing receptor (CaR) (CaSR) (hCasR) (Parathyroid cell calcium-sensing receptor 1) (PCaR1)	4.82	0.714
Q9H239	Matrix metalloproteinase-28 (MMP-28) (EC 3.4.24.-) (Epilysin)	4.81	0.552
O60911	Cathepsin L2 (EC 3.4.22.43) (Cathepsin U) (Cathepsin V)	4.79	0.383
P00533	Epidermal growth factor receptor (EC 2.7.10.1) (Proto-oncogene c-ErbB-1) (Receptor tyrosine-protein kinase erbB-1)	4.79	0.732
P36897	TGF-beta receptor type-1 (TGFR-1) (EC 2.7.11.30) (Activin A receptor type II-like protein kinase of 53kD) (Activin receptor-like kinase 5) (ALK-5) (ALK5) (Serine/threonine-protein kinase receptor R4) (SKR4) (TGF-beta type I receptor) (Transforming growth factor-beta receptor type I) (TGF-beta receptor type I) (TbetaR-I)	4.77	0.601
Q9NRP0	Oligosaccharyltransferase complex subunit OSTC (Hydrophobic protein HSF-28)	4.7	0.124
Q14210	Lymphocyte antigen 6D (Ly-6D) (E48 antigen)	4.69	0.527
P28070	Proteasome subunit beta type-4 (26 kDa prosomal protein) (HsBPROS26) (PROS-26) (Macropain beta chain) (Multicatalytic endopeptidase complex beta chain) (Proteasome beta chain) (Proteasome chain 3) (HsN3)	4.55	0.340
P55085	Proteinase-activated receptor 2 (PAR-2) (Coagulation factor II receptor-like 1) (G-protein coupled receptor 11) (Thrombin receptor-like 1) [Cleaved into: Proteinase-activated receptor 2, alternate cleaved 1; Proteinase-activated receptor 2, alternate cleaved 2]	4.53	0.309
Q9UBV4	Protein Wnt-16	4.38	0.566
Q86SJ6	Desmoglein-4 (Cadherin family member 13)	4.33	0.671
P35030	Trypsin-3 (EC 3.4.21.4) (Brain trypsinogen) (Mesotrypsin) (Mesotrypsinogen) (Serine protease 3) (Serine protease 4) (Trypsin III) (Trypsin IV)	4.28	0.387
Q16563	Synaptophysin-like protein 1 (Pantophysin)	4.25	0.156
P0DP57	Secreted Ly-6/uPAR domain-containing protein 2 (Secreted LY6/PLAUR domain-containing protein 2) (Secreted Ly-6/uPAR-related protein 2) (SLURP-2)	4.12	0.229
Q9Y2J8	Protein-arginine deiminase type-2 (EC 3.5.3.15) (PAD-H19) (Peptidylarginine deiminase II) (Protein-arginine deiminase type II)	4.06	0.416

Supplementary Table S3. Investigation of keratinocyte protein intrinsic disorder in proteome database.

Entry	Protein names	RAPID % Disorder	SLIDER IDR propensity score (0-1)
Q8WTS1	1-acylglycerol-3-phosphate O-acyltransferase ABHD5 (EC 2.3.1.51) (Abhydrolase domain-containing protein 5) (Lipid droplet-binding protein CGI-58)	4.01	0.455
P62330	ADP-ribosylation factor 6	4	0.303
P16234	Platelet-derived growth factor receptor alpha (PDGF-R-alpha) (PDGFR-alpha) (EC 2.7.10.1) (Alpha platelet-derived growth factor receptor) (Alpha-type platelet-derived growth factor receptor) (CD140 antigen-like family member A) (CD140a antigen) (Platelet-derived growth factor alpha receptor) (Platelet-derived growth factor receptor 2) (PDGFR-2) (CD antigen CD140a)	3.95	0.704
D3W0D1	Killer cell lectin-like receptor subfamily F member 2 (Lectin-like receptor F2) (Activating coreceptor NKp65)	3.86	0.166
P24723	Protein kinase C eta type (EC 2.7.11.13) (PKC-L) (nPKC-eta)	3.81	0.513
P55290	Cadherin-13 (Heart cadherin) (H-cadherin) (P105) (Truncated cadherin) (T-cad) (T-cadherin)	3.79	0.545
A0AV02	Solute carrier family 12 member 8 (Cation-chloride cotransporter 9)	3.78	0.586
Q9BPW9	Dehydrogenase/reductase SDR family member 9 (EC 1.1.1.209) (EC 1.1.1.53) (3-alpha hydroxysteroid dehydrogenase) (3-alpha-HSD) (NADP-dependent retinol dehydrogenase/reductase) (RDH-E2) (RDHL) (Retinol dehydrogenase 15) (EC 1.1.1.105) (Short chain dehydrogenase/reductase family 9C member 4) (Short-chain dehydrogenase/reductase retSDR8) (Tracheobronchial epithelial cell-specific retinol dehydrogenase) (RDH-TBE)	3.76	0.310
P05106	Integrin beta-3 (Platelet membrane glycoprotein IIIa) (GPIIIa) (CD antigen CD61)	3.68	0.670
P43235	Cathepsin K (EC 3.4.22.38) (Cathepsin O) (Cathepsin O2) (Cathepsin X)	3.65	0.396
Q8TD43	Transient receptor potential cation channel subfamily M member 4 (hTRPM4) (Calcium-activated non-selective cation channel 1) (Long transient receptor potential channel 4) (LTrpC-4) (LTrpC4) (Melastatin-4)	3.62	0.661
Q99650	Oncostatin-M-specific receptor subunit beta (Interleukin-31 receptor subunit beta) (IL-31 receptor subunit beta) (IL-31R subunit beta) (IL-31R-beta) (IL-31RB)	3.58	0.530
Q2M385	Macrophage-expressed gene 1 protein (Macrophage gene 1 protein) (Mpg-1) (Perforin-2) (P-2)	3.21	0.555
P29317	Ephrin type-A receptor 2 (EC 2.7.10.1) (Epithelial cell kinase) (Tyrosine-protein kinase receptor ECK)	3.18	0.698
P41221	Protein Wnt-5a	3.16	0.426
O75452	Retinol dehydrogenase 16 (EC 1.1.1.105) (EC 1.1.1.209) (EC 1.1.1.315) (EC 1.1.1.53) (Human epidermal retinol dehydrogenase) (hRDH-E) (Microsomal NAD(+)-dependent retinol dehydrogenase 4) (RoDH-4) (Short chain dehydrogenase/reductase family 9C member 8) (Sterol/retinol dehydrogenase)	3.15	0.347
P18564	Integrin beta-6	3.05	0.624
Q5VXJ0	Lipase member K (EC 3.1.1.-) (Lipase-like abhydrolase domain-containing protein 2)	3.01	0.325

Supplementary Table S3. Investigation of keratinocyte protein intrinsic disorder in proteome database.

Entry	Protein names	RAPID % Disorder	SLIDER IDR propensity score (0-1)
Q6TFL4	Kelch-like protein 24 (Kainate receptor-interacting protein for GluR6) (KRIP6) (Protein DRE1)	3	0.407
P81534	Beta-defensin 103 (Beta-defensin 3) (BD-3) (DEFB-3) (HBD3) (hBD-3) (Defensin, beta 103) (Defensin-like protein)	2.99	0.226
Q8N6L1	Keratinocyte-associated protein 2 (KCP-2) (Dolichyl-diphosphooligosaccharide--protein glycosyltransferase subunit KCP2) (Oligosaccharyl transferase subunit KCP2)	2.94	0.189
Q92819	Hyaluronan synthase 2 (EC 2.4.1.212) (Hyaluronate synthase 2) (Hyaluronic acid synthase 2) (HA synthase 2)	2.9	0.315
Q08188	Protein-glutamine gamma-glutamyltransferase E (EC 2.3.2.13) (Transglutaminase E) (TG(E)) (TGE) (TGase E) (Transglutaminase-3) (TGase-3) [Cleaved into: Protein-glutamine gamma-glutamyltransferase E 50 kDa catalytic chain; Protein-glutamine gamma-glutamyltransferase E 27 kDa non-catalytic chain]	2.89	0.576
Q14533	Keratin, type II cuticular Hb1 (Hair keratin K2.9) (Keratin, hair, basic, 1) (Keratin-81) (K81) (Metastatic lymph node 137 gene protein) (MLN 137) (Type II hair keratin Hb1) (Type-II keratin Kb21) (ghHKb1) (ghHb1)	2.77	0.700
Q9NYQ8	Protocadherin Fat 2 (hFat2) (Cadherin family member 8) (Multiple epidermal growth factor-like domains protein 1) (Multiple EGF-like domains protein 1)	2.69	0.679
P23229	Integrin alpha-6 (CD49 antigen-like family member F) (VLA-6) (CD antigen CD49f) [Cleaved into: Integrin alpha-6 heavy chain; Integrin alpha-6 light chain; Processed integrin alpha-6 (Alpha6p)]	2.57	0.643
O00206	Toll-like receptor 4 (EC 3.2.2.6) (hToll) (CD antigen CD284)	2.5	0.444
O60931	Cystinosin	2.45	0.145
Q9ULC6	Protein-arginine deiminase type-1 (EC 3.5.3.15) (Peptidylarginine deiminase I) (Protein-arginine deiminase type I)	2.41	0.533
Q9UNI1	Chymotrypsin-like elastase family member 1 (EC 3.4.21.36) (Elastase-1) (Pancreatic elastase 1)	2.33	0.242
Q9H1Y3	Opsin-3 (Encephalopsin) (Panopsin)	2.24	0.387
Q14574	Desmocollin-3 (Cadherin family member 3) (Desmocollin-4) (HT-CP)	2.23	0.661
Q02487	Desmocollin-2 (Cadherin family member 2) (Desmocollin-3) (Desmosomal glycoprotein II) (Desmosomal glycoprotein III)	2.22	0.724
Q9H9P8	L-2-hydroxyglutarate dehydrogenase, mitochondrial (EC 1.1.99.2) (Duranin)	2.16	0.471
Q9HB29	Interleukin-1 receptor-like 2 (EC 3.2.2.6) (IL-36 receptor) (IL-36R) (Interleukin-1 receptor-related protein 2) (IL-1Rrp2) (IL1R-rp2)	2.09	0.390
Q6YHK3	CD109 antigen (150 kDa TGF-beta-1-binding protein) (C3 and PZP-like alpha-2-macroglobulin domain-containing protein 7) (Platelet-specific Gov antigen) (p180) (r150) (CD antigen CD109)	2.01	0.582
P15509	Granulocyte-macrophage colony-stimulating factor receptor subunit alpha (GM-CSF-R-alpha) (GMCSFR-alpha) (GMR-alpha) (CDw116) (CD antigen CD116)	2	0.490
A8K2U0	Alpha-2-macroglobulin-like protein 1 (C3 and PZP-like alpha-2-macroglobulin domain-containing protein 9)	1.99	0.618

Supplementary Table S3. Investigation of keratinocyte protein intrinsic disorder in proteome database.

Entry	Protein names	RAPID % Disorder	SLIDER IDR propensity score (0-1)
P98194	Calcium-transporting ATPase type 2C member 1 (ATPase 2C1) (EC 7.2.2.10) (ATP-dependent Ca(2+) pump PMR1) (Ca(2+)/Mn(2+)-ATPase 2C1) (Secretory pathway Ca(2+)-transporting ATPase type 1) (SPCA1)	1.85	0.587
Q6UVW9	C-type lectin domain family 2 member A (Keratinocyte-associated C-type lectin) (KACL) (Proliferation-induced lymphocyte-associated receptor) (PILAR)	1.72	0.238
Q8N3Y7	Epidermal retinol dehydrogenase 2 (EPHD-2) (RDH-E2) (EC 1.1.1.105) (Retinal short-chain dehydrogenase reductase 2) (retSDR2) (Short-chain dehydrogenase/reductase family 16C member 5)	1.62	0.410
P08581	Hepatocyte growth factor receptor (HGF receptor) (EC 2.7.10.1) (HGF/SF receptor) (Proto-oncogene c-Met) (Scatter factor receptor) (SF receptor) (Tyrosine-protein kinase Met)	1.22	0.580
Q9Y5L3	Ectonucleoside triphosphate diphosphohydrolase 2 (NTPDase 2) (EC 3.6.1.-) (CD39 antigen-like 1) (Ecto-ATP diphosphohydrolase 2) (Ecto-ATPDase 2) (Ecto-ATPase 2)	1.21	0.481
Q9Y4K0	Lysyl oxidase homolog 2 (EC 1.4.3.13) (Lysyl oxidase-like protein 2) (Lysyl oxidase-related protein 2) (Lysyl oxidase-related protein WS9-14)	1.16	0.521
P05154	Plasma serine protease inhibitor (Acrosomal serine protease inhibitor) (Plasminogen activator inhibitor 3) (PAI-3) (PAI3) (Protein C inhibitor) (PCI) (Serpin A5)	0.99	0.550
Q9Y5Y6	Suppressor of tumorigenicity 14 protein (EC 3.4.21.109) (Matriptase) (Membrane-type serine protease 1) (MT-SP1) (Prostamin) (Serine protease 14) (Serine protease TADG-15) (Tumor-associated differentially-expressed gene 15 protein)	0.82	0.556
P05156	Complement factor I (EC 3.4.21.45) (C3B/C4B inactivator) [Cleaved into: Complement factor I heavy chain; Complement factor I light chain]	0.69	0.464
O75355	Ectonucleoside triphosphate diphosphohydrolase 3 (NTPDase 3) (EC 3.6.1.5) (CD39 antigen-like 3) (Ecto-ATP diphosphohydrolase 3) (Ecto-ATPDase 3) (Ecto-ATPase 3) (Ecto-apyrase 3) (HB6)	0.57	0.417
Q86UK0	Glucosylceramide transporter ABCA12 (EC 7.6.2.1) (ATP-binding cassette sub-family A member 12) (ATP-binding cassette transporter 12) (ATP-binding cassette 12)	0.54	0.655
Q5VXI9	Lipase member N (EC 3.1.1.-) (Lipase-like abhydrolase domain-containing protein 4)	0	0.376
P08217	Chymotrypsin-like elastase family member 2A (EC 3.4.21.71) (Elastase-2A)	0	0.223
